# Inter‐tissue relationships of gene expression in liver, muscle and adipose tissue of children with end‐stage chronic liver disease

**DOI:** 10.1002/jpn3.70498

**Published:** 2026-07-06

**Authors:** Eirini Kyrana, Cheng Zhang, Han Jin, Siamak Salehi, Jonathan C. K. Wells, Maesha Deheragoda, Ragai R. Mitry, Anil Dhawan

**Affiliations:** ^1^ Roger Williams Institute of Liver Studies King's College London London UK; ^2^ Paediatric Liver, GI and Nutrition Centre and MowatLabs King's College Hospital London UK; ^3^ Central Laboratory Tianjin Medical University General Hospital Tianjin China; ^4^ Department of Pathology Maastricht University Medical Centre+ Maastricht the Netherlands; ^5^ Childhood Nutrition Research Centre Great Ormond Street Institute of Child Health London UK

**Keywords:** liver transplant, microarray, sarcopenia

## Abstract

**Objectives:**

End‐stage chronic liver disease in children is associated with sarcopenia and aberrant adipose tissue mass. We investigated correlations between liver pathology‐associated gene pathways (fibrosis, inflammation and steatosis) and metabolic genes in muscle and adipose tissue.

**Methods:**

Liver, rectus abdominis muscle and subcutaneous adipose tissue were collected during liver transplant for microarray gene expression analysis. Patients underwent pre‐transplant indirect calorimetry, anthropometry and laboratory assessments. Weighted gene co‐expression network analysis identified highly correlated gene modules within each tissue and explored inter‐tissue correlations.

**Results:**

Nine patients were studied, three male:six female, age 7 months to 17 years. Liver gene clusters associated with fibrosis and ribosome function/protein secretion negatively correlated with muscle mitochondrial function genes and positively correlated with adipose tissue mitochondrial function genes. Notable correlations included a negative correlation between muscle growth hormone receptor (GHR) and liver ARID5B, MFGE8 and YWHAZ, and a positive correlation between adipose AKT1, ADG5, and SRM and liver RRAGA, YES1, EIF3M and COX3A. Liver inflammation‐associated genes (vimentin, TIMP2, CXCL6 and endothelin‐1) negatively correlated with adipose genes improving insulin sensitivity (THRSP) and fibrosis‐related genes (KRT36, DMTN). Liver steatosis genes (ADRA2B) negatively correlated with adipose genes involved in adipogenesis (FGF10) and thyroid hormone metabolism (NHLH1).

**Conclusions:**

Genes related to liver fibrosis and protein secretion negatively correlated with muscle and adipose tissue metabolism/proliferation genes. Liver inflammation and steatosis gene clusters were associated with muscle and adipose metabolism genes. This pilot study highlights important inter‐tissue gene correlations warranting further investigation in paediatric end‐stage chronic liver disease.

## Abstract (highlights)

**What is Known:**

- Children with end‐stage chronic liver disease suffer from sarcopenia and poor growth.
- Presence of sarcopenia and poor growth are associated with worse outcomes.

**What is New:**

- Liver fibrosis has a negative correlation with genes relating to mitochondrial function in muscle.
- Liver fibrosis has a negative correlation with the growth hormone receptor in muscle.
- Genes related to liver ribosome function and proliferation positively correlated with genes implicated in brown adipose tissue development, more efficient lipolysis and energy production.

## Graphical abstract

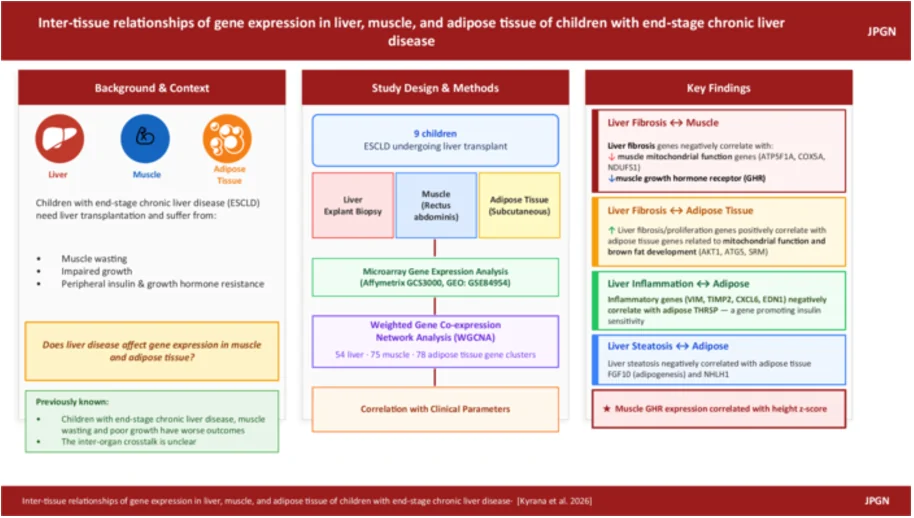

## 1 INTRODUCTION

Children with end‐stage chronic liver disease (ESCLD) require liver transplantation. ESCLD is associated with malnutrition, weight loss, sarcopenia, fluid retention, anaemia and metabolic disturbances. The diseased liver has reduced glycogen storage capacity, leading to early‐onset gluconeogenesis and accelerated starvation, reflected in a low respiratory quotient (RQ), indicating increased lipid oxidation.^1^, ^2^, ^3^, ^4^ These children experience poor growth due to malnutrition, malabsorption and metabolic changes, including peripheral insulin and growth hormone resistance, anorexia, muscle wasting and lipolysis.^1^, ^5^, ^6^, ^7^, ^8^, ^9^

These effects may result from disease progression or attempts at liver regeneration. Studies link liver fibrosis to sarcopenia.^10^, ^11^, ^12^ Hepatic stellate cell activation requires considerable energy, relying on glycolysis, glutaminolysis and de novo lipogenesis.^13^, ^14^, ^15^, ^16^, ^17^ Liver regeneration involves cytokines, growth factors and metabolic signalling, with substrate potentially provided by muscle or adipose tissue.^18^ In partial hepatectomy models, hepatic de novo lipogenesis initially supplies fatty acids for triglyceride synthesis, followed by uptake of lipolytic products from peripheral tissues.^19^

This pilot study investigated correlations between liver, muscle and adipose tissue gene expression in children undergoing liver transplantation, linking these to clinical parameters, including growth, RQ, blood investigations and liver explant scoring. The aim was to identify genes or pathways informing how liver disease influences muscle and adipose metabolism in children.

## 2 METHODS

### 2.1 Ethics statement

The study had the relevant regulatory and ethical approval: NRES Committee London—Central (11/LO/1146).

### 2.2 Sample collection and microarray

Children between the ages of 6 months and 18 years with ESCLD listed for liver transplant were prospectively enrolled on the study between January 2012 and June 2016. Children undergoing liver transplantation because of liver cancer or acute liver failure were excluded. All patients had consented to participate. All patients had their basic laboratory parameters and basic anthropometry recorded. Weight, height and body mass index were converted to standard deviation scores (SDS) using UK World Health Organisation reference data. The patients with ESCLD also had indirect calorimetry using the Deltatrac II metabolic monitor (Datex‐Ohmeda) and resting energy expenditure (REE) and RQ were recorded. RQ is the ratio of VCO_2_ and VO_2_. RQ varies from 0.67 (ketone body metabolism in the fasting state) to 1.3, reflecting lipogenesis from glucose or hyperventilation. RQ below 0.67 has been reported in patients with liver disease.^20^

Liver, muscle (abdominis rectus) and subcutaneous adipose tissue were collected at the time of liver transplant for the patients; samples were processed for gene expression by microarray analysis. Isolation of the total RNA, formation of cDNA and analysis of gene expression by microarray were done at the Genomics Centre at King's College London. The microarray was performed using the Affymetrix GCS3000 system with GeneChip arrays. The microarray data are minimal information about a microarray experiment (MIAME) compliant and have been deposited in the gene expression omnibus repository (accession number GSE84954).

Liver explants were graded for fibrosis (modified Ishak score), inflammation (modified Ishak score) and steatosis (grade 0–3). The histological grading was performed by a single‐blind histopathologist.

### 2.3 Co‐expression network analysis

Weighted gene co‐expression network analysis (WGCNA) was performed to identify gene modules that are highly correlated within each tissue type and to explore inter‐tissue correlations. Gene co‐expression networks were constructed separately for liver, muscle and adipose tissue samples using the WGCNA package in R (v1.70).^21^ For each tissue type, a signed adjacency matrix was generated using a soft‐thresholding power determined by a scale‐free topology model fit. The soft‐thresholding powers used were 18 for liver, 8 for muscle and 38 for adipose tissue, respectively, based on the scale‐free topology criterion.

The adjacency matrices were transformed into topological overlap matrices (TOM) to minimise the effects of noise and spurious associations. A hierarchical clustering tree (dendrogram) of genes was constructed using 1‐TOM as a distance measure, and gene modules were identified using the dynamic tree cut algorithm with a minimum module size of 30 genes. Modules with similar expression profiles were merged based on a dissimilarity threshold of 0.3 for the module eigengenes (MEs). For each gene module, the ME was calculated as the first principal component, representing the overall expression profile of the module.

Gene centrality analysis was performed to identify hub genes within each module using two metrics: (1) the correlation between gene expression and ME, and (2) the connectivity degree within the module. Genes with high values in both metrics were considered hub genes and potential key regulators within the module. Gene ontology (GO) enrichment analysis was conducted to characterise the biological functions of each module, using the clusterProfiler package (v4.0) with a Benjamini–Hochberg adjusted *p*‐value threshold of 0.05.

### 2.4 Statistical analysis

To investigate inter‐tissue relationships, we calculated Spearman correlations between MEs across different tissues. Additionally, Jaccard indices were calculated to assess the gene‐level overlap between modules from different tissues. These approaches allowed us to identify coordinated gene expression patterns across liver, muscle and adipose tissue in children with ESCLD. After applying Benjamini–Hochberg correction for multiple testing across all tests, only one association survived at false discovery rate (FDR) < 0.05. While formal multiple testing correction reduced the number of statistically significant findings, the ranking of associations was preserved. The Spearman correlation between raw and BH‐adjusted *p*‐value rankings is 0.996, confirming that the relative importance of findings was unchanged (Supporting Information S1: Table Methods). The key modules highlighted in our manuscript remain the top‐ranked associations regardless of whether multiple testing correction is applied. Claude AI and Grammarly were used in order to help with brainstorming and language editing.

## 3 RESULTS

Nine patients had samples collected (three male:six female), with ages ranging from 7 months to 17 years (median 3.1 years). Their diagnoses were biliary atresia (six patients), and one each of: alagille syndrome, alpha‐1‐antitrypsin deficiency and primary sclerosing cholangiopathy. All patients were having a liver transplant because of ESCLD. Median weight SDS was −1 (range −1.79 to −0.68), height SDS −1.31 (range −3.4 to −0.01) and MUAC SDS −2.2 (−5.29 to −0.85). From indirect calorimetry, RQ median 0.79 (range 0.72 to 1).

The liver tissue from two of the patients, one with neonatal sclerosing cholangitis and one with biliary atresia, was too small to yield enough RNA, but histology was still available.

### 3.1 WGCNA reveals tissue‐specific gene modules and their clinical associations

WGCNA identified 54 gene modules in liver tissue, 75 in muscle tissue and 78 in adipose tissue, each representing genes with similar expression patterns. GO enrichment analysis revealed their functional associations. We correlated MEs with clinical parameters, including histological parameters of fibrosis, steatosis and inflammation; metabolic indicators such as REE and RQ; and liver function markers, including bilirubin, albumin and urea. Our analysis (Supporting Information S1: Figure S1) revealed several robust correlations (| *r* | ≥ 0.7) between tissue‐specific modules and these clinical characteristics.

In liver tissue, modules such as Liver‐1, Liver‐2 and Liver‐7 demonstrated strong positive correlations with fibrosis scores, underscoring their involvement in extracellular matrix organisation, cell adhesion and proliferative processes. In muscle tissue, modules including Muscle‐57, Muscle‐38 and Muscle‐59 were significantly associated with metabolic parameters, suggesting metabolic disturbances may impact gene networks related to mitochondrial function and cellular respiration. Similarly, adipose tissue modules such as Fat‐1, Fat‐43 and Fat‐75 exhibited strong correlations with indicators of energy metabolism and liver function, implying a coordinated response between adipose tissue metabolic regulation and the systemic effects of liver disease. Table 1 presents the main gene clusters we will be examining and their significant associations with clinical parameters. Supporting Information S1: Figure S1 has a more comprehensive depiction of gene clusters and associations with clinical parameters.

**Table 1:** Basic demographics and anthropometry; correlations of tissue gene clusters with clinical parameters and correlations between tissue gene clusters.

1. Basic demographics and anthropometry							
Diagnosis	Age (years)	Sex	Wt_z	Ht_z	MUAC_z	RQ	Comments
BA	0.7	F	−0.73	−0.01	−2.37	0.82	NG feeds
Alagille syndrome	5.6	M	−1.44	−1.31	−0.85	1	PEG feeds
BA	1.1	M	0.68	−1.04	−2.2	0.72	Dietician input
A1AT	3.1	F	−1.29	−1.81	−2.2	0.83	Dietician input
BA	1.1	F	−1	−1.31	−1.72	0.81	Dietician input
BA	10.8	F	−0.15	−1.14	−0.93	0.78	Dietician input
BA	1.9	F	−1.79	−3.4	−5.29	0.74	NG feeds
PSC	17.7	M	−1.2	−2.24	−2.81	0.72	Dietician input
BA	9	F	−0.86	−0.83	−1.61	0.79	Dietician input
**2. Correlations of gene clusters with key clinical parameters**							
	**Clinical trait correlations (|*r*** | **≥ 0.7, *p*** < **0.05)**						
**Liver fibrosis**							
Liver‐1	Modified ishak fibrosis (+0.87); RQ (−0.77); albumin (−0.75); urea (−0.94)						
Liver‐2	Modified ishak fibrosis (+0.79); ALT (−0.92); RQ (−0.88); Eo (−0.86); GGT (−0.85)						
Muscle‐35	Modified ishak fibrosis (−0.85); RQ (+0.82); albumin (+0.73)						
Muscle‐5	None significant						
Fat‐1	Modified ishak fibrosis (+0.97); RQ (−0.94); urea (−0.83); Eo (−0.81)						
**Liver inflammation**							
Fat‐39	Modified ishak inflammation (−0.76)						
Liver‐4	Neu (+0.70); Mo (+0.72)						
**Liver steatosis**							
Liver‐6	Liver steatosis (+0.69); WCC (−0.7); Mo (−0.74); PLT (−0.75)						
Fat‐5	Liver steatosis (−0.86); BMI *z*‐score (−0.71), WCC (+0.81); Neu (+0.74); Ly (+0.87); PLT (+0.87); AST (+0.84), GGT (+0.74); ALP (+0.75).						
**3. Correlations between key gene clusters**							
**Correlation**							
Liver‐1	Muscle‐5	−0.67*					
Liver‐1	Muscle‐35	−0.78*					
Liver‐1	Fat‐1	0.82*					
Liver‐2	Muscle‐35	−0.75*					
Liver‐2	Fat‐1	0.85*					
Liver‐4	Fat‐39	−0.67*					
Liver‐6	Fat‐5	−0.75*					

*Note*: The first panel shows the diagnoses of the patients (BA, AS; A1AT and PSC), their age in years, sex (M and F), Wt_z, Ht_z, MUAC_z; and RQ as measured by indirect calorimetry. All patients were receiving dietitian input. Two of the younger ones with biliary atresia were receiving NG feeds, and the child with AS was on PEG feeds. None of the children were on parenteral nutrition and none were on steroids. The second panel shows the correlations between the main gene clusters discussed in the various sections of the manuscript and the clinical parameters. Liver tissue‐1, Liver tissue‐2, Muscle tissue‐35, Muscle tissue‐5 and Fat tissue‐1 in the Liver fibrosis section. Liver tissue‐4 and Fat tissue‐39 in the Liver Inflammation section. Liver tissue‐5 and Fat tissue‐49 in the Liver steatosis section. The third panel depicts the correlation between the gene clusters (Spearman correlation; *p < 0.05). Abbreviations: A1AT, alpha‐1‐antitrypsin deficiency; ALP, alkaline phosphatase; ALT, alanine aminotransferase; AS, alagille syndrome; AST, aspartate aminotransferase; Ba, basophils; BA, biliary atresia; BMI, body mass index; Eo, eosinophils; F, female; GGT, gamma‐glutamyl transferase; Ht_z, height *z*‐score; Ly, lymphocytes; M, male; MO, monocytes; MUAC_z, mid upper arm circumference *z*‐score; Neu, neutrophils; NG, nasogastric; PEG, gastrostomy; PLT, platelets; PSC, primary sclerosing cholangitis; RQ, respiratory quotient; WCC, white cell count; Wt_z, weight *z*‐score.

#### 3.1.1 Liver fibrosis

As seen in Table 1, gene clusters Liver‐1 and Liver‐2 showed strong positive correlations with the liver fibrosis score and strong negative correlations with RQ, albumin and urea levels—indirect markers of advanced liver disease. Gene clusters Muscle‐5 and Muscle‐35 had strong negative correlations with liver fibrosis scores and positive correlations with RQ, albumin and urea. The Fat‐1 gene cluster showed a strong positive correlation with the liver fibrosis score and strong negative correlations with RQ, albumin and urea.

As predicted, both Liver‐1 and Liver‐2 gene clusters had strong negative correlations with Muscle‐5 and Muscle‐35 and a strong positive correlation with Fat‐1 (Table 1). GO terms showed Liver‐1 genes were related to extracellular matrix and fibrosis, and Liver‐2 to ribosome function and proliferation. Muscle‐5 genes related to mitochondrial function and respiration, Muscle‐35 contained glucose‐sensing genes, and Fat‐1 genes were related to mitochondrial function. The correlation between the Fat‐1 module and fibrosis was also the only one that survived formal multiple‐testing correction at an FDR < 0.05.

**Figure 1:**
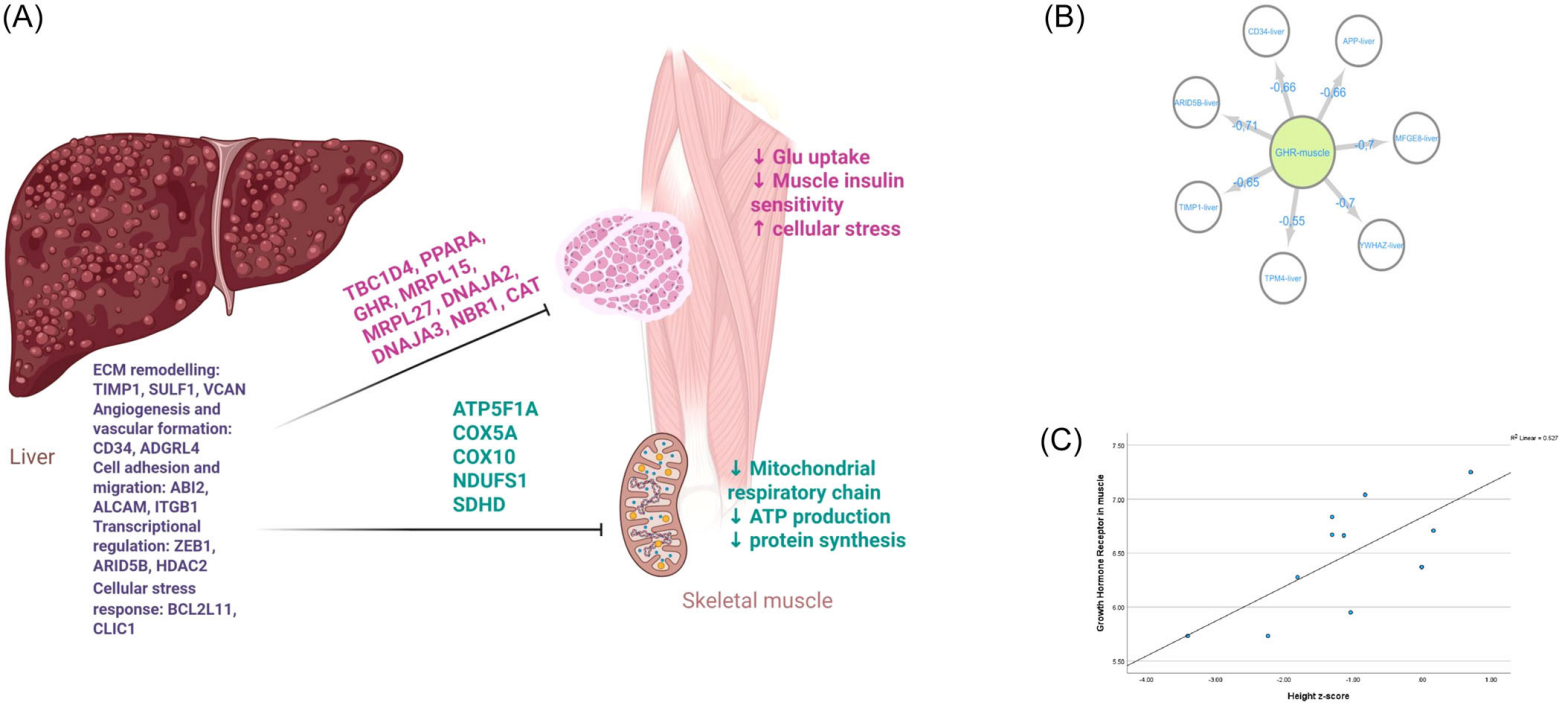
Gene correlations between Liver‐1 and Muscle‐5 gene clusters. (A) Illustration of key genes from Liver‐1 and their negative correlation with genes from Muscle‐5 gene clusters. (B) M uscle GHR shows significant negative correlations (*p* < 0.05) with liver genes, including CD34 (transmembrane phosphoglycoprotein), APP (amyloid precursor protein), MFGE8 (milk fat globule EGF factor 8/lactadherin), YWHAZ (14‐3‐3 zeta protein), TPM4 (tropomyosin 4), TIMP1 (metallopeptidase inhibitor 1) and ARID5B (AT‐rich interaction domain 5B). (C) Muscle GHR expression significantly correlates with patients’ height *z*‐score (SDS) (*p* < 0.05), linking growth hormone signalling to growth outcomes in children with chronic liver disease. GHR, growth hormone receptor; SDS, standard deviation scores.

Examining the top 5% in Liver‐1 and Muscle‐5 gene centrality, we identified 2102 pairs with strong correlations <−0.7 (*p* < 0.05), involving 65 liver genes and 83 muscle genes (Supporting Information S1: Table S1 and Supporting Information S1: Figure S2). Top Liver‐1 genes involved tissue remodelling, inflammation, cell adhesion and extracellular matrix regulation—key processes in fibrosis development. The strongly negative correlation between mitochondrial genes in muscle and liver fibrosis genes suggests decreased muscle energy production capacity (Figure 1A).

Genes like ATP5F1A, COX5A, COX10, NDUFS1 and SDHD in muscle are components of the mitochondrial respiratory chain, and their negative correlation could indicate lower energy availability. Negative correlation with TBC1D4 (glucose uptake) and PPARA (metabolic regulation) suggests reduced glucose utilisation, contributing to peripheral insulin resistance. Negative correlation with mitochondrial ribosomal proteins (MRPL15 and MRPL27) indicates a potential decrease in mitochondrial protein synthesis. Negative correlation with heat shock proteins (DNAJA2 and DNAJA3) and autophagy receptors (NBR1) may impair cellular stress handling and protein clearance, further contributing to muscle wasting. Reduced expression of antioxidant genes like CAT (catalase) may increase oxidative stress in muscle tissue.

The growth hormone receptor (GHR) in muscle is of particular interest in children (Figure 1B,C).^5^, ^22^ In this data, GHR in muscle correlated positively with height *z*‐score. The GHR gene in muscle was negatively correlated with the following three genes in the Liver‐1 gene cluster: ARID‐5B (regulates fatty acid metabolism and proliferation at the pre B‐cell stage), MFGE8 (also known as lactadherin; among its related pathways are regulation of insulin‐like growth factor [IGF] transport and uptake by IGF‐binding proteins^23^; produced by mesenchymal stromal cells and has an anti‐fibrotic effect in liver via transforming growth factor‐beta^24^, ^25^) and YWHAZ (a highly conserved housekeeping gene which interacts with IRS‐1 suggesting a role in regulating insulin sensitivity). Other genes of interest in Muscle‐5, such as TBC1D4 and CAT, show strong negative correlations with ARID‐5B, MFGE8 and YWHAZ, as well as with APP (amyloid precursor protein, which has been reported to be elevated in biliary atresia and advanced fibrosis^26^).

Examining top 5% in Liver‐1 and Muscle‐35 identified 123 pairs with correlations <−0.7 (*p* < 0.05), including 54 liver genes and six muscle genes (Supporting Information S1: Table S2). Key muscle genes were GEMIN7, ISCU, inosine triphosphatase (ITPA), MYL2, RNF212B and SEC.14L5. These genes participate in maintaining mitochondrial function, genetic stability, lipid homoeostasis^23^ and cellular repair. Their negative correlation with liver fibrosis genes may indicate a role in muscle catabolism.

Examining top 5% in Liver‐2 and Muscle‐35 identified 26 pairs with correlations <−0.7 (*p* < 0.05), involving 22 liver genes and three muscle genes (Supporting Information S1: Table S3 and Supporting Information S1: Figure S3). Liver genes were linked to cell growth regulation, protein synthesis, gene expression regulation and cellular trafficking. Most of these genes have been associated with HCC, whereas the patients we studied did not have HCC, as evidenced by the histology of their liver explants. Key muscle genes with strong negative correlations were ITPA, KIR2DS4 and MEN1. ITPA is involved in purine metabolism, prevents the incorporation of noncanonical purine nucleotides into DNA and RNA, KIR2DS4 (killer cell immunoglobulin‐like receptor) in immune system regulation, particularly involving NK and subsets of T‐cells, and MEN1 (Menin 1) is a tumour suppressor, transcriptional regulator associated with multiple endocrine neoplasia type 1.

**Figure 2:**
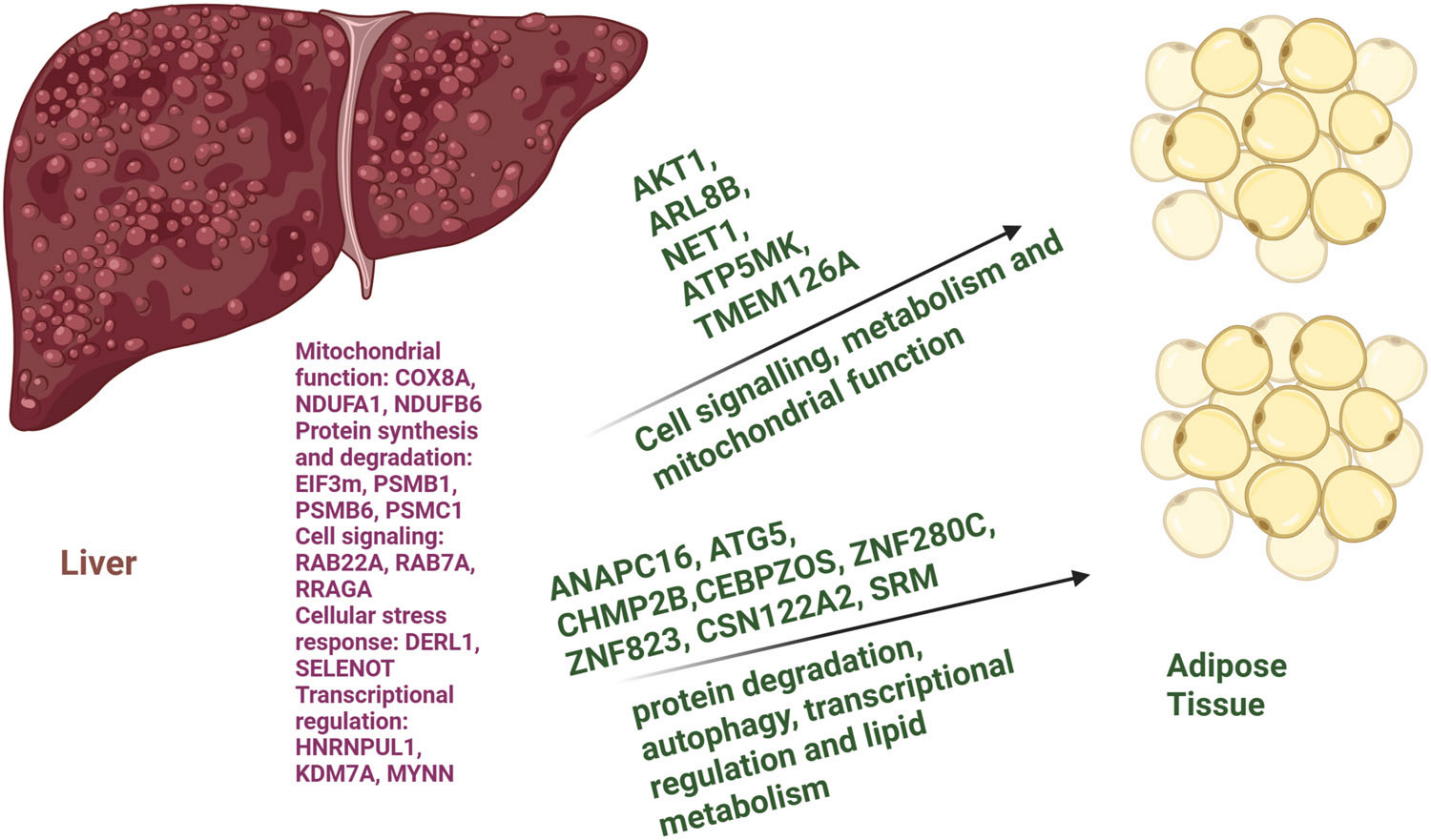
Illustration of the positive correlation of genes in the Liver‐1 and Liver‐2 gene clusters with genes in the Fat‐1 gene cluster. Heatmap of genes from Liver‐2 gene cluster with significant (*p* < 0.05) correlations with genes from Muscle‐35 and Fat‐1 gene clusters.

We explored positive correlations between Liver‐1, Liver‐2 and Fat‐1. Examining the top 5% in Liver‐1 and Fat‐1 (Figure 2) identified 142 pairs with correlations >0.7 (*p* < 0.05), involving 60 liver genes and 13 adipose tissue genes (Figure 2, Supporting Information S1: Table S4 and Supporting Information S1: Figure S2). Liver‐1 genes related to ECM remodelling, angiogenesis, cell adhesion, transcriptional regulation and cellular stress response had strong positive correlations with Fat‐1 genes involved in ribosome biogenesis, protein synthesis, cellular transport, signalling and mitochondrial function, suggesting coordinated transcriptional response between liver and adipose tissue.

Examining the top 5% in Liver‐2 and Fat‐1 identified 421 pairs with correlations >0.7 (*p* < 0.05), involving 51 liver genes and 32 adipose tissue genes (Figure 2, Supporting Information S1: Table S5 and Supporting Information S1: Figure S3). Liver‐2 genes were associated with mitochondrial function, protein synthesis and degradation, cell signalling, cellular stress response and transcriptional regulation. Fat‐1 genes are related to cell signalling and metabolism, protein degradation and autophagy, mitochondrial function, transcriptional regulation and lipid metabolism.

Genes of particular interest included AKT1 (required for brown adipose tissue development),^24^ ATG5 (activates autophagy and promotes lower adipose tissue mass),^25^ RRAGA (senses amino acid availability) and SRM (spermidine synthase, linked to adipose tissue thermogenesis).^26^, ^27^ The strong positive correlations suggest coordinated responses to the energy crisis across organs.^28^, ^29^

#### 3.1.2 Liver inflammation

Certain clusters correlated with liver inflammation score, but their intercorrelations and correlations with clinical parameters were not significant. Liver‐4 gene cluster correlated significantly with blood neutrophils (0.7, *p* < 0.05) and monocytes (0.7, *p* < 0.05), and had a significant negative correlation with gene cluster Fat‐39 (−0.67, *p* < 0.05). Fat‐39 had a strongly significant negative correlation with liver inflammation on histology (−0.76, *p* < 0.05).

**Figure 3:**
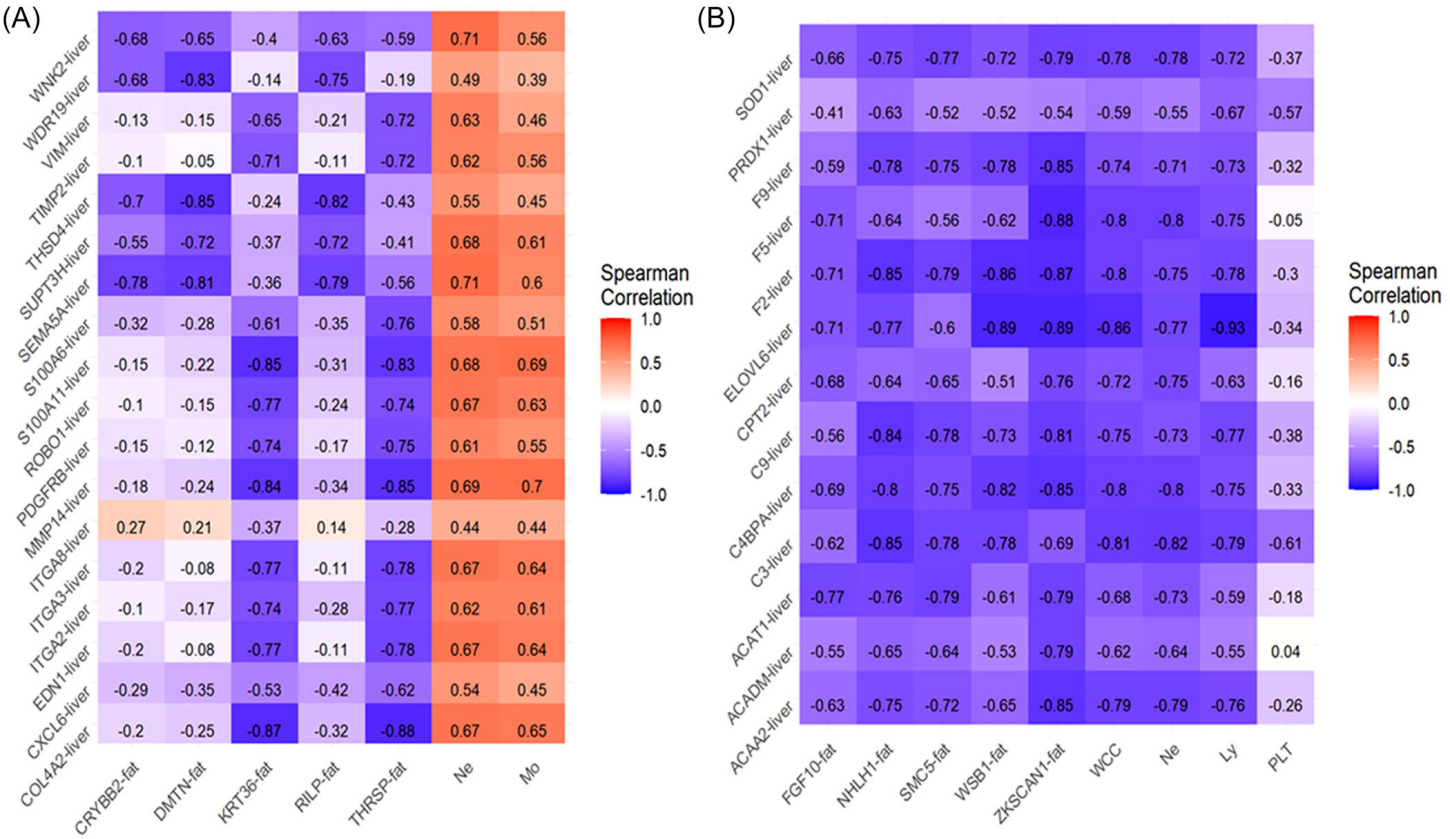
Gene correlations related to liver inflammation and liver steatosis. (A) Liver inflammation. Heatmap showing correlations between Liver‐4 and Fat‐39 gene clusters with neutrophil and monocyte counts (*p* < 0.05). Liver‐4 and Fat‐39 clusters were negatively correlated (*r* = −0.67, *p* < 0.05), with Fat‐39 showing negative correlation with liver inflammation (−0.76, *p* < 0.05). (B) Liver steatosis. Heatmap showing correlations between Liver‐6 and Fat‐5 gene clusters and blood parameters, including WCC, neutrophils, lymphocytes and platelets (*p* < 0.05). Liver‐6 positively correlated with histological steatosis and negatively with Fat‐5 (*r* = −0.75, *p* < 0.02), linking gene expression patterns to liver pathology and systemic immune profiles. WCC, white cell count.

Top genes in Fat‐39 (*p* < 0.05 with strong correlations >−0.65 with Liver‐4 genes) were CRYBB2, DMTN, KRT36, RILP, RPL9L and THRSP (Figure 3A). The most interesting is THRSP (thyroid hormone‐responsive gene), which is related to insulin sensitivity. Significant genes in Liver‐4 included EDN1 (endothelin 1), PDGFRB, MMP14, S100A11/S100A6, TIMP2/COL4A2, VIM (vimentin) and genes involved in inflammatory cell recruitment like CXCL6, ITGA2/ITGA3/ITGB8 and ROBO1 (Supporting Information S1: Table S6).

#### 3.1.3 Liver steatosis

Supporting Information S1: Tables S7 and S8 show significant liver‐muscle gene correlations with steatosis, although the significance of these correlations remains unclear.

Adipose tissue gene clusters associated with liver steatosis were inversely correlated with white blood cells, neutrophils, lymphocytes and platelets. Liver genes associated with upregulating metabolic enzymes (ACAA2, ACADM and ACAT1), increasing lipid processing (ELOVL6 and CPT2), and activating coagulation and complement cascades (C3, C4BPA, C9, F2, F5, F9, SOD1 and PRDX1) were positively correlated with liver steatosis. They showed negative correlations with adipose tissue genes, including FGF10, NHLH1, SMC5, WSB1 and ZKSCAN1 (Supporting Information S1: Tables S9 and S10), and with full blood count parameters (Figure 3B). FGF10 is essential for white adipose tissue development.^30^ These adipose tissue genes are primarily involved in maintaining adipose tissue health and function and are relevant to inter‐organ communication during liver disease.

GO enrichment pathways for the key gene clusters of interest are shown in Supporting Information S1: Table S11.

## 4 DISCUSSION

This study examines the interplay among liver, muscle and adipose tissues in children with ESCLD undergoing liver transplantation, using WGCNA. The analysis focused on gene clusters related to clinical parameters. We focused on gene clusters that correlated with liver fibrosis scoring on histology and clinically with low RQ, a well‐accepted characteristic of cirrhotic patients.^20^ The identification of negative correlations between genes related to liver fibrosis and genes related to mitochondrial function in muscle is consistent with the literature supporting mitochondrial dysfunction in muscle as one mechanism of sarcopenia in chronic diseases.^31^, ^32^ The negative association with genes related to insulin sensitivity and glucose uptake in muscle suggests that peripheral insulin resistance, as observed in cirrhosis, may be a possible underlying mechanism for muscle pathology.^7^, ^33^ It is important to highlight that we did not have histological or functional evidence of muscle dysfunction for these patients.

The discovery of a negative correlation between GHR in muscle tissue and liver fibrosis is particularly relevant for paediatric patients, as it may partly explain the impaired growth commonly observed in these children. The liver produces IGF‐1; therefore, levels are low in children with cirrhosis. Additionally, growth hormone resistance has been well described in these children,^34^ with earlier reports suggesting it may contribute to lipolysis resistance,^5^ possibly to preserve energy stores. It would be valuable to investigate, in future functional studies, whether GHR downregulation in muscle underlies this anabolic resistance.

The liver gene cluster related to ribosome function and proliferation (Liver‐2) is positively correlated with adipose tissue genes implicated in brown adipose tissue development and more efficient lipolysis and energy production, including AKT1, ATG5 and SRM. During a disease process such as cirrhosis, various pathways may be affected, while others are activated to counteract these effects. For example, as liver disease progresses, some pathways may increase energy production via lipolysis, whilst others may protect energy stores by developing anabolic resistance. Depending on the balance between these processes and their evolution during disease progression, the net effect on the patient may range from preservation of muscle and adipose tissue to varying degrees of muscle loss and adipose tissue loss.

The patients included in this study, and their liver explants, were not particularly characterised by inflammation or steatosis; therefore, findings in these categories were less pronounced. Nevertheless, the negative association of THRSP in adipose tissue with inflammation‐associated parameters in the liver warrants further investigation as a potential step in explaining inflammation‐mediated peripheral insulin resistance.^35^, ^36^ Insulin increases THRSP mRNA expression five‐ to eight‐fold during euglycemic hyperinsulinemia.^36^ This induction is impaired in insulin‐resistant subjects, and THRSP expression closely correlates with whole‐body insulin sensitivity. This gene and its negative correlation with upregulated fibro‐inflammation genes, including EDN1, MMP14, VIMENTIN, CXCL6 and ITGA2/ITGA3, may suggest a pathway for the development of inflammation‐mediated insulin resistance. These same genes positively correlated with neutrophil and monocyte counts in peripheral blood.

In this cohort of patients with ESCLD who were not overweight or obese, liver histological steatosis had a negative correlation with inflammatory markers in peripheral blood, but a positive correlation with hepatic genes promoting lipid processing and energy production and the inflammatory and coagulation cascade, such as ACAA2, ACAT1, CPT2, C3, C9, F2 and F9. In adipose tissue, steatosis correlated with genes involved in maintaining adipose tissue health and function, such as FGF10. Further functional studies looking into adipose tissue turnover would be of great interest in these patients.

This study has limitations. The small sample size limits the generalisability of these findings, and larger validation studies are needed to confirm these gene expression patterns. Specifically, the small sample size (*n* = 9 ESCLD patients), diagnostic heterogeneity (biliary atresia predominated, *n* = 7/9), the wide age range (0.7–17.7 years), and the presence of potential other clinical confounders mean that these findings are hypothesis‐generating and require further validation. This study identifies correlations; however, further work is required to establish causal relationships, and functional studies are needed to delineate how these gene networks interact in vivo. Such studies are necessary to determine how these findings can be translated to therapeutic interventions aimed at maintaining muscle and adipose tissue health in cirrhosis.

## 5 CONCLUSION

This cross‐sectional observational study identifies associations between tissue‐specific gene expression in children with ESCLD that warrant functional validation in experimental models. Findings suggest associations with pathways related to mitochondrial dysfunction, peripheral insulin resistance and GHR in muscle, as well as potentially compensatory pathways in adipose tissue. This work provides some insight into how ESCLD may affect systemic energy homeostasis.

## CONFLICT OF INTEREST STATEMENT

The authors declare no conflicts of interest.

## Supporting information

Table showing the total module‐trait correlation tests performed across all three tissues. **Supplement Figure 1**. Heatmap showing Spearman correlations between tissue‐specific module eigengenes (liver, muscle, adipose) and clinical parameters, including liver fibrosis scores (Ishak, modified Ishak), REE/Wt, RQ, serum albumin, urea, steatosis, and inflammation. Modules with strong correlations (| r | ≥ 0.7) are highlighted; warm colours indicate positive associations, cool colours negative. Tissue‐specific modules (Liver‐1, Muscle‐57, Fat‐1) are shown on the y‐axis, clinical parameters on the x‐axis. The visualisation reveals strong associations between liver modules and fibrosis, muscle modules and metabolic indicators, and adipose modules with energy regulation in children with end‐stage chronic liver disease. **Supplement Figure 2.** Spearman correlations between key genes from Liver‐1, Muscle‐5, and Fat‐1 clusters. Heatmap of genes from Liver‐1 gene cluster with significant (P < 0.05) correlations with genes from Muscle‐5 and Fat‐1 gene clusters. **Supplement Figure 3.** Heatmap of correlations of genes between Liver‐2, Fat‐1, and Muscle‐35. Heatmap of genes from Liver‐2 gene cluster with significant (P < 0.05) correlations with genes from Muscle‐35 and Fat‐1 gene clusters. **Supplement** Table 1 represents the top 5% in Liver‐1 gene centrality and in Muscle‐5, with calculated Spearman correlations between the two groups, focusing only on strong correlations of <−0.7 with a p < 0.05. There were 2102 pairs (65 liver genes and 83 muscle genes). **Supplement Table 2** represents the top 5% in Liver‐1 gene centrality and in Muscle‐35, with calculated Spearman correlations between the two groups, focusing only on strong correlations of <−0.7 with a p < 0.05. **Supplement Table 3** represents the top 5% in Liver‐2 gene centrality and in Muscle‐35, with calculated Spearman correlations between the two groups, focusing only on strong correlations of <−0.7 with a p < 0.05. There were 2102 pairs (65 liver genes and 83 muscle genes). **Supplement Table 4** represents the top 5% in Liver‐1 gene centrality and in Fat‐1, with calculated Spearman correlations between the two groups, focusing only on strong correlations of >0.7 with a p < 0.05. **Supplement Table 5** represents the top 5% in Liver‐2 gene centrality and in Fat‐1, with calculated Spearman correlations between the two groups, focusing only on strong correlations of >0.6 with a p < 0.05. **Supplement Table 6** represents the top 5% in Liver‐4 gene centrality and in Fat‐39, with calculated Spearman correlations between the two groups, focusing only on strong correlations of <−0.6 with a p < 0.05. **Supplement Table 7** represents the top 5% in Liver‐5 gene centrality and Muscle‐63, with calculated Spearman correlations between the two groups, focusing only on strong correlations of >0.60.6 with a p < 0.05. Liver‐5 gene cluster correlated with liver histological steatosis (−0.9, p < 0.01) and Muscle‐63 with liver histological steatosis (−0.69, p < 0.05) and the correlation between the two gene groups was 0.7, p < 0.05. **Supplement Table 8** represents the top 5% in Liver‐39 gene centrality and Muscle‐54, with calculated Spearman correlations between the two groups, focusing only on strong correlations of <−0.81 with a p < 0.05. Liver‐39 gene cluster correlated with liver histological steatosis (0.75, p < 0.05) and Muscle‐54 with liver histological steatosis (−0.67, p < 0.05) and the correlation between the two gene groups was −0.81, p < 0.01. **Supplement Table 9** represents the top 5% in Liver‐5 gene centrality and in Fat‐49, with calculated Spearman correlations between the two groups, focusing only on strong correlations of >0.6 with a p < 0.05. **Supplement Table 10** represents the top 5% in Liver‐39 gene centrality and in Fat‐5, Liver‐39 and Fat‐43 and Liver‐39 and Fat‐19 with calculated Spearman correlations between the two groups, focusing only on strong correlations of over I0.6I with a p < 0.05. **Supplement Table 11** shows that, using GO enrichment pathways for the key gene clusters of interest, Liver‐1 genes were associated with extracellular matrix and fibrosis, whereas Liver‐2 genes were associated with ribosome function and proliferation. Muscle‐5 genes related to mitochondrial function and respiration, Muscle‐35 contained glucose‐sensing genes, and Fat‐1 genes were related to mitochondrial function.
