# Supplementary material for: Inter‐tissue relationships of gene expression in liver, muscle and adipose tissue of children with end‐stage chronic liver disease
Source: J Pediatr Gastroenterol Nutr. 2026 Jul 6;83(3):500–8. doi: 10.1002/jpn3.70498 (PMC13539642; doi:10.1002/jpn3.70498)
Supplement: Supplementary file 1 [file JPN3-83-500-s001.docx]

**Supplement Table Methods**

| **Category** | **Description** | **Value** |
| --- | --- | --- |
| OVERALL SUMMARY |  |  |
| Total module-trait tests | All tissues combined | 5796 |
| Significant (\|r\|>=0.7, p<0.05) | Original approach | 269 |
| Significant after BH correction | FDR < 0.05 | 2 |
| Significant after BH correction | FDR < 0.10 | 2 |
| Significant after BH correction | FDR < 0.20 | 2 |
|  |  |  |
| BY TISSUE |  |  |
| Liver | 54 modules x 28 traits | 1512 |
| - Nominal significant | \|r\|>=0.7, p<0.05 | 91 |
| - BH significant | FDR < 0.05 | 0 |
| Muscle | 75 modules x 28 traits | 2100 |
| - Nominal significant | \|r\|>=0.7, p<0.05 | 77 |
| - BH significant | FDR < 0.05 | 0 |
| Fat | 78 modules x 28 traits | 2184 |
| - Nominal significant | \|r\|>=0.7, p<0.05 | 101 |
| - BH significant | FDR < 0.05 | 2 |

Table showing the total module-trait correlation tests performed across all three tissues: 1,512 tests in liver (54 modules × 28 clinical traits), 2,100 tests in muscle (75 modules × 28 traits), and 2,184 tests in adipose tissue (78 modules × 28 traits). Using our original approach with a stringent correlation threshold (|r| ≥ 0.7) and nominal significance (p < 0.05), we identified 269 module-trait associations.

After applying Benjamini-Hochberg correction for multiple testing across all 5,796 tests, only 2 associations survive at FDR < 0.05. Both are the Fat-1 module correlation with fibrosis scores (r = 0.97, FDR = 0.031), reflecting the same biological finding with two fibrosis measures (Metavir and Modified Ishak).

**Supplement Figure 1**

**Supplement Figure 1.** Heatmap showing Spearman correlations between tissue-specific module eigengenes (liver, muscle, adipose) and clinical parameters, including liver fibrosis scores (Ishak, modified Ishak), REE/Wt, RQ, serum albumin, urea, steatosis, and inflammation. Modules with strong correlations (|r| ≥ 0.7) are highlighted; warm colours indicate positive associations, cool colours negative. Tissue-specific modules (Liver-1, Muscle-57, Fat-1) are shown on the y-axis, clinical parameters on the x-axis. The visualisation reveals strong associations between liver modules and fibrosis, muscle modules and metabolic indicators, and adipose modules with energy regulation in children with end-stage chronic liver disease.

**LIVER FIBROSIS**

**Supplement Figure 2**

**Supplement Figure 2.** Spearman correlations between key genes from Liver-1, Muscle-5, and Fat-1 clusters. Heatmap of genes from Liver-1 gene cluster with significant (P<0.05) correlations with genes from Muscle-5 and Fat-1 gene clusters.

**Supplement Figure 3**

**Supplement Figure 3**. Heatmap of correlations of genes between Liver-2, Fat-1, and Muscle-35. Heatmap of genes from Liver-2 gene cluster with significant (P<0.05) correlations with genes from Muscle-35 and Fat-1 gene clusters.

**Supplement Table 1**

| **Liver-1** | **Muscle-5** | **Correlation** | **p_value** | **adjusted_p_value** |
| --- | --- | --- | --- | --- |
| ADGRL4-liver | ACADM-muscle | -0.70909 | 0.014552 | 0.050131 |
| ANXA5-liver | ACADM-muscle | -0.71818 | 0.0128 | 0.048396 |
| APP-liver | ACADM-muscle | -0.85455 | 0.000807 | 0.022594 |
| ARHGAP18-liver | ACADM-muscle | -0.7 | 0.016471 | 0.052234 |
| BMPR2-liver | ACADM-muscle | -0.70909 | 0.014552 | 0.050131 |
| CD34-liver | ACADM-muscle | -0.7 | 0.016471 | 0.052234 |
| CDH5-liver | ACADM-muscle | -0.71818 | 0.0128 | 0.048396 |
| CLIC1-liver | ACADM-muscle | -0.73636 | 0.00976 | 0.043199 |
| DEFB1-liver | ACADM-muscle | -0.75455 | 0.007282 | 0.038865 |
| DSG2-liver | ACADM-muscle | -0.79091 | 0.003746 | 0.032221 |
| FNBP1L-liver | ACADM-muscle | -0.72727 | 0.011205 | 0.045908 |
| HEG1-liver | ACADM-muscle | -0.70909 | 0.014552 | 0.050131 |
| IFT57-liver | ACADM-muscle | -0.73636 | 0.00976 | 0.043199 |
| IPO7-liver | ACADM-muscle | -0.70909 | 0.014552 | 0.050131 |
| ITGB1-liver | ACADM-muscle | -0.78182 | 0.004473 | 0.033769 |
| KHDRBS1-liver | ACADM-muscle | -0.79091 | 0.003746 | 0.032221 |
| KIF3A-liver | ACADM-muscle | -0.70909 | 0.014552 | 0.050131 |
| MFGE8-liver | ACADM-muscle | -0.7 | 0.016471 | 0.052234 |
| MMGT1-liver | ACADM-muscle | -0.76364 | 0.006233 | 0.036383 |
| MYH9-liver | ACADM-muscle | -0.8 | 0.00311 | 0.031511 |
| NCKAP1-liver | ACADM-muscle | -0.78182 | 0.004473 | 0.033769 |
| PRKX-liver | ACADM-muscle | -0.73636 | 0.00976 | 0.043199 |
| RAP2A-liver | ACADM-muscle | -0.70909 | 0.014552 | 0.050131 |
| SLC25A6-liver | ACADM-muscle | -0.79091 | 0.003746 | 0.032221 |
| STX7-liver | ACADM-muscle | -0.7 | 0.016471 | 0.052234 |
| SUCO-liver | ACADM-muscle | -0.73636 | 0.00976 | 0.043199 |
| SULF1-liver | ACADM-muscle | -0.73636 | 0.00976 | 0.043199 |
| TES-liver | ACADM-muscle | -0.74545 | 0.008455 | 0.041717 |
| TIMP1-liver | ACADM-muscle | -0.7 | 0.016471 | 0.052234 |
| TMSB10-liver | ACADM-muscle | -0.72727 | 0.011205 | 0.045908 |
| TPM4-liver | ACADM-muscle | -0.73636 | 0.00976 | 0.043199 |
| TSPAN14-liver | ACADM-muscle | -0.7 | 0.016471 | 0.052234 |
| YWHAZ-liver | ACADM-muscle | -0.77273 | 0.005299 | 0.035326 |
| ANXA5-liver | AFG3L2-muscle | -0.73636 | 0.00976 | 0.043199 |
| APP-liver | AFG3L2-muscle | -0.82727 | 0.001677 | 0.02618 |
| ARHGAP18-liver | AFG3L2-muscle | -0.71818 | 0.0128 | 0.048396 |
| ARID5B-liver | AFG3L2-muscle | -0.74545 | 0.008455 | 0.041717 |
| CD34-liver | AFG3L2-muscle | -0.76364 | 0.006233 | 0.036383 |
| CDH5-liver | AFG3L2-muscle | -0.73636 | 0.00976 | 0.043199 |
| CLIC1-liver | AFG3L2-muscle | -0.77273 | 0.005299 | 0.035326 |
| DSG2-liver | AFG3L2-muscle | -0.77273 | 0.005299 | 0.035326 |
| EZR-liver | AFG3L2-muscle | -0.7 | 0.016471 | 0.052234 |
| FNBP1L-liver | AFG3L2-muscle | -0.73636 | 0.00976 | 0.043199 |
| HEG1-liver | AFG3L2-muscle | -0.74545 | 0.008455 | 0.041717 |
| IFT57-liver | AFG3L2-muscle | -0.72727 | 0.011205 | 0.045908 |
| ITGB1-liver | AFG3L2-muscle | -0.75455 | 0.007282 | 0.038865 |
| KHDRBS1-liver | AFG3L2-muscle | -0.75455 | 0.007282 | 0.038865 |
| KIF3A-liver | AFG3L2-muscle | -0.7 | 0.016471 | 0.052234 |
| MFGE8-liver | AFG3L2-muscle | -0.76364 | 0.006233 | 0.036383 |
| MMGT1-liver | AFG3L2-muscle | -0.75455 | 0.007282 | 0.038865 |
| MYH9-liver | AFG3L2-muscle | -0.74545 | 0.008455 | 0.041717 |
| NCKAP1-liver | AFG3L2-muscle | -0.72727 | 0.011205 | 0.045908 |
| PRKX-liver | AFG3L2-muscle | -0.73636 | 0.00976 | 0.043199 |
| RAP2A-liver | AFG3L2-muscle | -0.75455 | 0.007282 | 0.038865 |
| SLC25A6-liver | AFG3L2-muscle | -0.76364 | 0.006233 | 0.036383 |
| SMIM10-liver | AFG3L2-muscle | -0.70909 | 0.014552 | 0.050131 |
| STX7-liver | AFG3L2-muscle | -0.75455 | 0.007282 | 0.038865 |
| SUCO-liver | AFG3L2-muscle | -0.74545 | 0.008455 | 0.041717 |
| SULF1-liver | AFG3L2-muscle | -0.77273 | 0.005299 | 0.035326 |
| TES-liver | AFG3L2-muscle | -0.72727 | 0.011205 | 0.045908 |
| TIMP1-liver | AFG3L2-muscle | -0.75455 | 0.007282 | 0.038865 |
| TMSB10-liver | AFG3L2-muscle | -0.73636 | 0.00976 | 0.043199 |
| TPM4-liver | AFG3L2-muscle | -0.70909 | 0.014552 | 0.050131 |
| VCAN-liver | AFG3L2-muscle | -0.71818 | 0.0128 | 0.048396 |
| YWHAZ-liver | AFG3L2-muscle | -0.81818 | 0.002083 | 0.027717 |
| TTF2-liver | AKT1S1-muscle | -0.70909 | 0.014552 | 0.050131 |
| BMPR2-liver | ANO5-muscle | -0.7 | 0.016471 | 0.052234 |
| APP-liver | ASB10-muscle | -0.75455 | 0.007282 | 0.038865 |
| ARID5B-liver | ASB10-muscle | -0.72727 | 0.011205 | 0.045908 |
| CD34-liver | ASB10-muscle | -0.7 | 0.016471 | 0.052234 |
| CLIC1-liver | ASB10-muscle | -0.70909 | 0.014552 | 0.050131 |
| DSG2-liver | ASB10-muscle | -0.72727 | 0.011205 | 0.045908 |
| FNBP1L-liver | ASB10-muscle | -0.7 | 0.016471 | 0.052234 |
| HEG1-liver | ASB10-muscle | -0.7 | 0.016471 | 0.052234 |
| ITGB1-liver | ASB10-muscle | -0.7 | 0.016471 | 0.052234 |
| MFGE8-liver | ASB10-muscle | -0.71818 | 0.0128 | 0.048396 |
| PLPP5-liver | ASB10-muscle | -0.73636 | 0.00976 | 0.043199 |
| RAP2A-liver | ASB10-muscle | -0.7 | 0.016471 | 0.052234 |
| SULF1-liver | ASB10-muscle | -0.70909 | 0.014552 | 0.050131 |
| YWHAZ-liver | ASB10-muscle | -0.76364 | 0.006233 | 0.036383 |
| APP-liver | ATP5F1A-muscle | -0.78182 | 0.004473 | 0.033769 |
| BMPR2-liver | ATP5F1A-muscle | -0.71818 | 0.0128 | 0.048396 |
| DSG2-liver | ATP5F1A-muscle | -0.76364 | 0.006233 | 0.036383 |
| FNBP1L-liver | ATP5F1A-muscle | -0.7 | 0.016471 | 0.052234 |
| IPO7-liver | ATP5F1A-muscle | -0.70909 | 0.014552 | 0.050131 |
| ITGB1-liver | ATP5F1A-muscle | -0.75455 | 0.007282 | 0.038865 |
| MMGT1-liver | ATP5F1A-muscle | -0.73636 | 0.00976 | 0.043199 |
| NCKAP1-liver | ATP5F1A-muscle | -0.73636 | 0.00976 | 0.043199 |
| SLC25A6-liver | ATP5F1A-muscle | -0.72727 | 0.011205 | 0.045908 |
| SUCO-liver | ATP5F1A-muscle | -0.7 | 0.016471 | 0.052234 |
| TTF2-liver | ATP5F1A-muscle | -0.71818 | 0.0128 | 0.048396 |
| ADGRL4-liver | ATP5MJ-muscle | -0.74545 | 0.008455 | 0.041717 |
| ANXA5-liver | ATP5MJ-muscle | -0.73636 | 0.00976 | 0.043199 |
| APP-liver | ATP5MJ-muscle | -0.9 | 0.00016 | 0.016927 |
| ARHGAP18-liver | ATP5MJ-muscle | -0.76364 | 0.006233 | 0.036383 |
| B4GALT5-liver | ATP5MJ-muscle | -0.71818 | 0.0128 | 0.048396 |
| BMPR2-liver | ATP5MJ-muscle | -0.79091 | 0.003746 | 0.032221 |
| CA5B-liver | ATP5MJ-muscle | -0.73636 | 0.00976 | 0.043199 |
| CCN4-liver | ATP5MJ-muscle | -0.70909 | 0.014552 | 0.050131 |
| CD34-liver | ATP5MJ-muscle | -0.73636 | 0.00976 | 0.043199 |
| CDH5-liver | ATP5MJ-muscle | -0.7 | 0.016471 | 0.052234 |
| CLIC1-liver | ATP5MJ-muscle | -0.77273 | 0.005299 | 0.035326 |
| DEFB1-liver | ATP5MJ-muscle | -0.80909 | 0.002559 | 0.029155 |
| DSG2-liver | ATP5MJ-muscle | -0.84545 | 0.001045 | 0.024446 |
| FNBP1L-liver | ATP5MJ-muscle | -0.79091 | 0.003746 | 0.032221 |
| HEG1-liver | ATP5MJ-muscle | -0.72727 | 0.011205 | 0.045908 |
| IFT57-liver | ATP5MJ-muscle | -0.72727 | 0.011205 | 0.045908 |
| IPO7-liver | ATP5MJ-muscle | -0.77273 | 0.005299 | 0.035326 |
| ITGB1-liver | ATP5MJ-muscle | -0.83636 | 0.001333 | 0.025685 |
| KHDRBS1-liver | ATP5MJ-muscle | -0.8 | 0.00311 | 0.031511 |
| KIF3A-liver | ATP5MJ-muscle | -0.76364 | 0.006233 | 0.036383 |
| MFGE8-liver | ATP5MJ-muscle | -0.7 | 0.016471 | 0.052234 |
| MMGT1-liver | ATP5MJ-muscle | -0.81818 | 0.002083 | 0.027717 |
| MYH9-liver | ATP5MJ-muscle | -0.8 | 0.00311 | 0.031511 |
| NCKAP1-liver | ATP5MJ-muscle | -0.82727 | 0.001677 | 0.02618 |
| PRKX-liver | ATP5MJ-muscle | -0.76364 | 0.006233 | 0.036383 |
| RALB-liver | ATP5MJ-muscle | -0.73636 | 0.00976 | 0.043199 |
| RAP2A-liver | ATP5MJ-muscle | -0.78182 | 0.004473 | 0.033769 |
| RPS6KA2-liver | ATP5MJ-muscle | -0.70909 | 0.014552 | 0.050131 |
| SLC25A6-liver | ATP5MJ-muscle | -0.81818 | 0.002083 | 0.027717 |
| SMIM10-liver | ATP5MJ-muscle | -0.74545 | 0.008455 | 0.041717 |
| STX7-liver | ATP5MJ-muscle | -0.75455 | 0.007282 | 0.038865 |
| SUCO-liver | ATP5MJ-muscle | -0.79091 | 0.003746 | 0.032221 |
| SULF1-liver | ATP5MJ-muscle | -0.77273 | 0.005299 | 0.035326 |
| TES-liver | ATP5MJ-muscle | -0.79091 | 0.003746 | 0.032221 |
| TIMP1-liver | ATP5MJ-muscle | -0.70909 | 0.014552 | 0.050131 |
| TMSB10-liver | ATP5MJ-muscle | -0.76364 | 0.006233 | 0.036383 |
| TPM4-liver | ATP5MJ-muscle | -0.8 | 0.00311 | 0.031511 |
| TSPAN14-liver | ATP5MJ-muscle | -0.70909 | 0.014552 | 0.050131 |
| TTF2-liver | ATP5MJ-muscle | -0.77273 | 0.005299 | 0.035326 |
| YWHAZ-liver | ATP5MJ-muscle | -0.80909 | 0.002559 | 0.029155 |
| MMGT1-liver | ATP6V1H-muscle | -0.70909 | 0.014552 | 0.050131 |
| TTF2-liver | ATP8A1-muscle | -0.76364 | 0.006233 | 0.036383 |
| ABI2-liver | C6orf136-muscle | -0.76364 | 0.006233 | 0.036383 |
| ADGRL4-liver | C6orf136-muscle | -0.82727 | 0.001677 | 0.02618 |
| ANXA5-liver | C6orf136-muscle | -0.85455 | 0.000807 | 0.022594 |
| APP-liver | C6orf136-muscle | -0.86364 | 0.000612 | 0.022041 |
| ARHGAP18-liver | C6orf136-muscle | -0.89091 | 0.000233 | 0.018341 |
| ARID5B-liver | C6orf136-muscle | -0.89091 | 0.000233 | 0.018341 |
| B4GALT5-liver | C6orf136-muscle | -0.70909 | 0.014552 | 0.050131 |
| BMPR2-liver | C6orf136-muscle | -0.72727 | 0.011205 | 0.045908 |
| CAMSAP2-liver | C6orf136-muscle | -0.75455 | 0.007282 | 0.038865 |
| CCN4-liver | C6orf136-muscle | -0.85455 | 0.000807 | 0.022594 |
| CD34-liver | C6orf136-muscle | -0.82727 | 0.001677 | 0.02618 |
| CDH5-liver | C6orf136-muscle | -0.87273 | 0.000455 | 0.020203 |
| CLIC1-liver | C6orf136-muscle | -0.81818 | 0.002083 | 0.027717 |
| DEFB1-liver | C6orf136-muscle | -0.82727 | 0.001677 | 0.02618 |
| DSG2-liver | C6orf136-muscle | -0.87273 | 0.000455 | 0.020203 |
| EPS8-liver | C6orf136-muscle | -0.83636 | 0.001333 | 0.025685 |
| EZR-liver | C6orf136-muscle | -0.77273 | 0.005299 | 0.035326 |
| FNBP1L-liver | C6orf136-muscle | -0.85455 | 0.000807 | 0.022594 |
| HDAC2-liver | C6orf136-muscle | -0.76364 | 0.006233 | 0.036383 |
| HEG1-liver | C6orf136-muscle | -0.82727 | 0.001677 | 0.02618 |
| IFT57-liver | C6orf136-muscle | -0.85455 | 0.000807 | 0.022594 |
| ITGB1-liver | C6orf136-muscle | -0.82727 | 0.001677 | 0.02618 |
| KHDRBS1-liver | C6orf136-muscle | -0.84545 | 0.001045 | 0.024446 |
| KIF3A-liver | C6orf136-muscle | -0.84545 | 0.001045 | 0.024446 |
| MFGE8-liver | C6orf136-muscle | -0.90909 | 0.000106 | 0.016759 |
| MFSD6-liver | C6orf136-muscle | -0.8 | 0.00311 | 0.031511 |
| MMGT1-liver | C6orf136-muscle | -0.90909 | 0.000106 | 0.016759 |
| MTMR6-liver | C6orf136-muscle | -0.72727 | 0.011205 | 0.045908 |
| MYH9-liver | C6orf136-muscle | -0.83636 | 0.001333 | 0.025685 |
| NCKAP1-liver | C6orf136-muscle | -0.81818 | 0.002083 | 0.027717 |
| PLPP5-liver | C6orf136-muscle | -0.8 | 0.00311 | 0.031511 |
| PRKX-liver | C6orf136-muscle | -0.80909 | 0.002559 | 0.029155 |
| PRRC2C-liver | C6orf136-muscle | -0.78182 | 0.004473 | 0.033769 |
| RALB-liver | C6orf136-muscle | -0.78182 | 0.004473 | 0.033769 |
| RAP2A-liver | C6orf136-muscle | -0.84545 | 0.001045 | 0.024446 |
| RPS6KA2-liver | C6orf136-muscle | -0.79091 | 0.003746 | 0.032221 |
| SLC25A6-liver | C6orf136-muscle | -0.88182 | 0.00033 | 0.018341 |
| SMIM10-liver | C6orf136-muscle | -0.79091 | 0.003746 | 0.032221 |
| STX7-liver | C6orf136-muscle | -0.88182 | 0.00033 | 0.018341 |
| SUCO-liver | C6orf136-muscle | -0.9 | 0.00016 | 0.016927 |
| SULF1-liver | C6orf136-muscle | -0.81818 | 0.002083 | 0.027717 |
| TES-liver | C6orf136-muscle | -0.85455 | 0.000807 | 0.022594 |
| TIMP1-liver | C6orf136-muscle | -0.83636 | 0.001333 | 0.025685 |
| TMSB10-liver | C6orf136-muscle | -0.81818 | 0.002083 | 0.027717 |
| TPM4-liver | C6orf136-muscle | -0.79091 | 0.003746 | 0.032221 |
| TSPAN14-liver | C6orf136-muscle | -0.8 | 0.00311 | 0.031511 |
| VCAN-liver | C6orf136-muscle | -0.86364 | 0.000612 | 0.022041 |
| WDR1-liver | C6orf136-muscle | -0.71818 | 0.0128 | 0.048396 |
| YWHAZ-liver | C6orf136-muscle | -0.82727 | 0.001677 | 0.02618 |
| ABI2-liver | CAT-muscle | -0.77273 | 0.005299 | 0.035326 |
| ADGRL4-liver | CAT-muscle | -0.89091 | 0.000233 | 0.018341 |
| AGPAT5-liver | CAT-muscle | -0.7 | 0.016471 | 0.052234 |
| ALCAM-liver | CAT-muscle | -0.84545 | 0.001045 | 0.024446 |
| ANXA5-liver | CAT-muscle | -0.84545 | 0.001045 | 0.024446 |
| AP2B1-liver | CAT-muscle | -0.75455 | 0.007282 | 0.038865 |
| APP-liver | CAT-muscle | -0.88182 | 0.00033 | 0.018341 |
| ARHGAP18-liver | CAT-muscle | -0.9 | 0.00016 | 0.016927 |
| ARID5B-liver | CAT-muscle | -0.79091 | 0.003746 | 0.032221 |
| B4GALT5-liver | CAT-muscle | -0.77273 | 0.005299 | 0.035326 |
| BMPR2-liver | CAT-muscle | -0.78182 | 0.004473 | 0.033769 |
| CA5B-liver | CAT-muscle | -0.73636 | 0.00976 | 0.043199 |
| CAMSAP2-liver | CAT-muscle | -0.76364 | 0.006233 | 0.036383 |
| CAPNS1-liver | CAT-muscle | -0.70909 | 0.014552 | 0.050131 |
| CCN4-liver | CAT-muscle | -0.86364 | 0.000612 | 0.022041 |
| CD34-liver | CAT-muscle | -0.78182 | 0.004473 | 0.033769 |
| CDH5-liver | CAT-muscle | -0.88182 | 0.00033 | 0.018341 |
| CLIC1-liver | CAT-muscle | -0.79091 | 0.003746 | 0.032221 |
| DEFB1-liver | CAT-muscle | -0.90909 | 0.000106 | 0.016759 |
| DSG2-liver | CAT-muscle | -0.9 | 0.00016 | 0.016927 |
| EPS8-liver | CAT-muscle | -0.82727 | 0.001677 | 0.02618 |
| EZR-liver | CAT-muscle | -0.74545 | 0.008455 | 0.041717 |
| FNBP1L-liver | CAT-muscle | -0.87273 | 0.000455 | 0.020203 |
| HDAC2-liver | CAT-muscle | -0.81818 | 0.002083 | 0.027717 |
| HEG1-liver | CAT-muscle | -0.78182 | 0.004473 | 0.033769 |
| IFT57-liver | CAT-muscle | -0.82727 | 0.001677 | 0.02618 |
| IPO7-liver | CAT-muscle | -0.8 | 0.00311 | 0.031511 |
| ITGB1-liver | CAT-muscle | -0.86364 | 0.000612 | 0.022041 |
| KHDRBS1-liver | CAT-muscle | -0.88182 | 0.00033 | 0.018341 |
| KIF3A-liver | CAT-muscle | -0.87273 | 0.000455 | 0.020203 |
| KIF5B-liver | CAT-muscle | -0.7 | 0.016471 | 0.052234 |
| MFGE8-liver | CAT-muscle | -0.84545 | 0.001045 | 0.024446 |
| MFSD6-liver | CAT-muscle | -0.82727 | 0.001677 | 0.02618 |
| MMGT1-liver | CAT-muscle | -0.92727 | 3.97E-05 | 0.016759 |
| MTMR6-liver | CAT-muscle | -0.78182 | 0.004473 | 0.033769 |
| MYH9-liver | CAT-muscle | -0.87273 | 0.000455 | 0.020203 |
| NCKAP1-liver | CAT-muscle | -0.88182 | 0.00033 | 0.018341 |
| PLPP5-liver | CAT-muscle | -0.79091 | 0.003746 | 0.032221 |
| PRKX-liver | CAT-muscle | -0.82727 | 0.001677 | 0.02618 |
| PRRC2C-liver | CAT-muscle | -0.84545 | 0.001045 | 0.024446 |
| RALB-liver | CAT-muscle | -0.87273 | 0.000455 | 0.020203 |
| RAP2A-liver | CAT-muscle | -0.82727 | 0.001677 | 0.02618 |
| RPS6KA2-liver | CAT-muscle | -0.82727 | 0.001677 | 0.02618 |
| SLC25A6-liver | CAT-muscle | -0.91818 | 6.66E-05 | 0.016759 |
| SMIM10-liver | CAT-muscle | -0.77273 | 0.005299 | 0.035326 |
| STX7-liver | CAT-muscle | -0.86364 | 0.000612 | 0.022041 |
| SUCO-liver | CAT-muscle | -0.91818 | 6.66E-05 | 0.016759 |
| SULF1-liver | CAT-muscle | -0.79091 | 0.003746 | 0.032221 |
| TES-liver | CAT-muscle | -0.89091 | 0.000233 | 0.018341 |
| TIMP1-liver | CAT-muscle | -0.8 | 0.00311 | 0.031511 |
| TMSB10-liver | CAT-muscle | -0.80909 | 0.002559 | 0.029155 |
| TPM4-liver | CAT-muscle | -0.81818 | 0.002083 | 0.027717 |
| TSPAN14-liver | CAT-muscle | -0.80909 | 0.002559 | 0.029155 |
| TTF2-liver | CAT-muscle | -0.84545 | 0.001045 | 0.024446 |
| VCAN-liver | CAT-muscle | -0.81818 | 0.002083 | 0.027717 |
| WDR1-liver | CAT-muscle | -0.78182 | 0.004473 | 0.033769 |
| YWHAZ-liver | CAT-muscle | -0.78182 | 0.004473 | 0.033769 |
| TTF2-liver | CLASP2-muscle | -0.73636 | 0.00976 | 0.043199 |
| ADGRL4-liver | COQ10A-muscle | -0.72727 | 0.011205 | 0.045908 |
| ANKRD27-liver | COQ10A-muscle | -0.71818 | 0.0128 | 0.048396 |
| ANXA5-liver | COQ10A-muscle | -0.71818 | 0.0128 | 0.048396 |
| APP-liver | COQ10A-muscle | -0.83636 | 0.001333 | 0.025685 |
| ARHGAP18-liver | COQ10A-muscle | -0.82727 | 0.001677 | 0.02618 |
| ARID5B-liver | COQ10A-muscle | -0.79091 | 0.003746 | 0.032221 |
| BMPR2-liver | COQ10A-muscle | -0.79091 | 0.003746 | 0.032221 |
| CCN4-liver | COQ10A-muscle | -0.76364 | 0.006233 | 0.036383 |
| CD34-liver | COQ10A-muscle | -0.72727 | 0.011205 | 0.045908 |
| CDH5-liver | COQ10A-muscle | -0.73636 | 0.00976 | 0.043199 |
| CLIC1-liver | COQ10A-muscle | -0.75455 | 0.007282 | 0.038865 |
| DEFB1-liver | COQ10A-muscle | -0.75455 | 0.007282 | 0.038865 |
| DSG2-liver | COQ10A-muscle | -0.84545 | 0.001045 | 0.024446 |
| EPS8-liver | COQ10A-muscle | -0.78182 | 0.004473 | 0.033769 |
| EZR-liver | COQ10A-muscle | -0.76364 | 0.006233 | 0.036383 |
| FNBP1L-liver | COQ10A-muscle | -0.80909 | 0.002559 | 0.029155 |
| HDAC2-liver | COQ10A-muscle | -0.70909 | 0.014552 | 0.050131 |
| HEG1-liver | COQ10A-muscle | -0.8 | 0.00311 | 0.031511 |
| IFT57-liver | COQ10A-muscle | -0.78182 | 0.004473 | 0.033769 |
| ITGB1-liver | COQ10A-muscle | -0.80909 | 0.002559 | 0.029155 |
| KHDRBS1-liver | COQ10A-muscle | -0.76364 | 0.006233 | 0.036383 |
| KIF3A-liver | COQ10A-muscle | -0.76364 | 0.006233 | 0.036383 |
| KIF5B-liver | COQ10A-muscle | -0.73636 | 0.00976 | 0.043199 |
| MFGE8-liver | COQ10A-muscle | -0.79091 | 0.003746 | 0.032221 |
| MFSD6-liver | COQ10A-muscle | -0.7 | 0.016471 | 0.052234 |
| MMGT1-liver | COQ10A-muscle | -0.86364 | 0.000612 | 0.022041 |
| MYH9-liver | COQ10A-muscle | -0.72727 | 0.011205 | 0.045908 |
| NCKAP1-liver | COQ10A-muscle | -0.80909 | 0.002559 | 0.029155 |
| PLPP5-liver | COQ10A-muscle | -0.81818 | 0.002083 | 0.027717 |
| PRKX-liver | COQ10A-muscle | -0.74545 | 0.008455 | 0.041717 |
| PRRC2C-liver | COQ10A-muscle | -0.70909 | 0.014552 | 0.050131 |
| RAP2A-liver | COQ10A-muscle | -0.8 | 0.00311 | 0.031511 |
| SLC25A6-liver | COQ10A-muscle | -0.80909 | 0.002559 | 0.029155 |
| SMIM10-liver | COQ10A-muscle | -0.77273 | 0.005299 | 0.035326 |
| STX7-liver | COQ10A-muscle | -0.74545 | 0.008455 | 0.041717 |
| SUCO-liver | COQ10A-muscle | -0.81818 | 0.002083 | 0.027717 |
| SULF1-liver | COQ10A-muscle | -0.75455 | 0.007282 | 0.038865 |
| TES-liver | COQ10A-muscle | -0.75455 | 0.007282 | 0.038865 |
| TMSB10-liver | COQ10A-muscle | -0.77273 | 0.005299 | 0.035326 |
| TPM4-liver | COQ10A-muscle | -0.79091 | 0.003746 | 0.032221 |
| TSPAN14-liver | COQ10A-muscle | -0.73636 | 0.00976 | 0.043199 |
| VCAN-liver | COQ10A-muscle | -0.81818 | 0.002083 | 0.027717 |
| YWHAZ-liver | COQ10A-muscle | -0.78182 | 0.004473 | 0.033769 |
| ADGRL4-liver | COX10-muscle | -0.70909 | 0.014552 | 0.050131 |
| ANXA5-liver | COX10-muscle | -0.8 | 0.00311 | 0.031511 |
| APP-liver | COX10-muscle | -0.90909 | 0.000106 | 0.016759 |
| ARHGAP18-liver | COX10-muscle | -0.82727 | 0.001677 | 0.02618 |
| ARID5B-liver | COX10-muscle | -0.75455 | 0.007282 | 0.038865 |
| BMPR2-liver | COX10-muscle | -0.8 | 0.00311 | 0.031511 |
| CA5B-liver | COX10-muscle | -0.80909 | 0.002559 | 0.029155 |
| CCN4-liver | COX10-muscle | -0.74545 | 0.008455 | 0.041717 |
| CD34-liver | COX10-muscle | -0.85455 | 0.000807 | 0.022594 |
| CDH5-liver | COX10-muscle | -0.79091 | 0.003746 | 0.032221 |
| CLIC1-liver | COX10-muscle | -0.87273 | 0.000455 | 0.020203 |
| DEFB1-liver | COX10-muscle | -0.73636 | 0.00976 | 0.043199 |
| DSG2-liver | COX10-muscle | -0.87273 | 0.000455 | 0.020203 |
| EPS8-liver | COX10-muscle | -0.76364 | 0.006233 | 0.036383 |
| EZR-liver | COX10-muscle | -0.80909 | 0.002559 | 0.029155 |
| FNBP1L-liver | COX10-muscle | -0.86364 | 0.000612 | 0.022041 |
| HEG1-liver | COX10-muscle | -0.80909 | 0.002559 | 0.029155 |
| IFT57-liver | COX10-muscle | -0.7 | 0.016471 | 0.052234 |
| IPO7-liver | COX10-muscle | -0.73636 | 0.00976 | 0.043199 |
| ITGB1-liver | COX10-muscle | -0.85455 | 0.000807 | 0.022594 |
| KHDRBS1-liver | COX10-muscle | -0.84545 | 0.001045 | 0.024446 |
| KIF3A-liver | COX10-muscle | -0.81818 | 0.002083 | 0.027717 |
| KIF5B-liver | COX10-muscle | -0.76364 | 0.006233 | 0.036383 |
| MFGE8-liver | COX10-muscle | -0.75455 | 0.007282 | 0.038865 |
| MFSD6-liver | COX10-muscle | -0.77273 | 0.005299 | 0.035326 |
| MMGT1-liver | COX10-muscle | -0.83636 | 0.001333 | 0.025685 |
| MYH9-liver | COX10-muscle | -0.77273 | 0.005299 | 0.035326 |
| NCKAP1-liver | COX10-muscle | -0.86364 | 0.000612 | 0.022041 |
| PLPP5-liver | COX10-muscle | -0.76364 | 0.006233 | 0.036383 |
| PRKX-liver | COX10-muscle | -0.87273 | 0.000455 | 0.020203 |
| PRRC2C-liver | COX10-muscle | -0.77273 | 0.005299 | 0.035326 |
| RALB-liver | COX10-muscle | -0.76364 | 0.006233 | 0.036383 |
| RAP2A-liver | COX10-muscle | -0.89091 | 0.000233 | 0.018341 |
| RPS6KA2-liver | COX10-muscle | -0.73636 | 0.00976 | 0.043199 |
| SLC25A6-liver | COX10-muscle | -0.84545 | 0.001045 | 0.024446 |
| SMIM10-liver | COX10-muscle | -0.85455 | 0.000807 | 0.022594 |
| STX7-liver | COX10-muscle | -0.85455 | 0.000807 | 0.022594 |
| SUCO-liver | COX10-muscle | -0.84545 | 0.001045 | 0.024446 |
| SULF1-liver | COX10-muscle | -0.87273 | 0.000455 | 0.020203 |
| TES-liver | COX10-muscle | -0.83636 | 0.001333 | 0.025685 |
| TIMP1-liver | COX10-muscle | -0.82727 | 0.001677 | 0.02618 |
| TMSB10-liver | COX10-muscle | -0.83636 | 0.001333 | 0.025685 |
| TPM4-liver | COX10-muscle | -0.82727 | 0.001677 | 0.02618 |
| TSPAN14-liver | COX10-muscle | -0.72727 | 0.011205 | 0.045908 |
| TTF2-liver | COX10-muscle | -0.71818 | 0.0128 | 0.048396 |
| VCAN-liver | COX10-muscle | -0.76364 | 0.006233 | 0.036383 |
| YWHAZ-liver | COX10-muscle | -0.9 | 0.00016 | 0.016927 |
| ADGRL4-liver | COX5A-muscle | -0.7 | 0.016471 | 0.052234 |
| ANXA5-liver | COX5A-muscle | -0.77273 | 0.005299 | 0.035326 |
| APP-liver | COX5A-muscle | -0.87273 | 0.000455 | 0.020203 |
| ARHGAP18-liver | COX5A-muscle | -0.73636 | 0.00976 | 0.043199 |
| ARID5B-liver | COX5A-muscle | -0.74545 | 0.008455 | 0.041717 |
| CCN4-liver | COX5A-muscle | -0.71818 | 0.0128 | 0.048396 |
| CD34-liver | COX5A-muscle | -0.8 | 0.00311 | 0.031511 |
| CDH5-liver | COX5A-muscle | -0.73636 | 0.00976 | 0.043199 |
| CLIC1-liver | COX5A-muscle | -0.80909 | 0.002559 | 0.029155 |
| DEFB1-liver | COX5A-muscle | -0.70909 | 0.014552 | 0.050131 |
| DSG2-liver | COX5A-muscle | -0.80909 | 0.002559 | 0.029155 |
| EZR-liver | COX5A-muscle | -0.70909 | 0.014552 | 0.050131 |
| FNBP1L-liver | COX5A-muscle | -0.77273 | 0.005299 | 0.035326 |
| HEG1-liver | COX5A-muscle | -0.75455 | 0.007282 | 0.038865 |
| IFT57-liver | COX5A-muscle | -0.73636 | 0.00976 | 0.043199 |
| ITGB1-liver | COX5A-muscle | -0.8 | 0.00311 | 0.031511 |
| KHDRBS1-liver | COX5A-muscle | -0.79091 | 0.003746 | 0.032221 |
| KIF3A-liver | COX5A-muscle | -0.73636 | 0.00976 | 0.043199 |
| MFGE8-liver | COX5A-muscle | -0.77273 | 0.005299 | 0.035326 |
| MMGT1-liver | COX5A-muscle | -0.77273 | 0.005299 | 0.035326 |
| MYH9-liver | COX5A-muscle | -0.79091 | 0.003746 | 0.032221 |
| NCKAP1-liver | COX5A-muscle | -0.76364 | 0.006233 | 0.036383 |
| PRKX-liver | COX5A-muscle | -0.77273 | 0.005299 | 0.035326 |
| RAP2A-liver | COX5A-muscle | -0.78182 | 0.004473 | 0.033769 |
| RPS6KA2-liver | COX5A-muscle | -0.71818 | 0.0128 | 0.048396 |
| SLC25A6-liver | COX5A-muscle | -0.8 | 0.00311 | 0.031511 |
| SMIM10-liver | COX5A-muscle | -0.73636 | 0.00976 | 0.043199 |
| STX7-liver | COX5A-muscle | -0.79091 | 0.003746 | 0.032221 |
| SUCO-liver | COX5A-muscle | -0.77273 | 0.005299 | 0.035326 |
| SULF1-liver | COX5A-muscle | -0.80909 | 0.002559 | 0.029155 |
| TES-liver | COX5A-muscle | -0.77273 | 0.005299 | 0.035326 |
| TIMP1-liver | COX5A-muscle | -0.79091 | 0.003746 | 0.032221 |
| TMSB10-liver | COX5A-muscle | -0.76364 | 0.006233 | 0.036383 |
| TPM4-liver | COX5A-muscle | -0.74545 | 0.008455 | 0.041717 |
| VCAN-liver | COX5A-muscle | -0.71818 | 0.0128 | 0.048396 |
| YWHAZ-liver | COX5A-muscle | -0.85455 | 0.000807 | 0.022594 |
| TTF2-liver | COX5B-muscle | -0.8 | 0.00311 | 0.031511 |
| APP-liver | COX6A2-muscle | -0.74545 | 0.008455 | 0.041717 |
| CD34-liver | COX6A2-muscle | -0.7 | 0.016471 | 0.052234 |
| CLIC1-liver | COX6A2-muscle | -0.73636 | 0.00976 | 0.043199 |
| DSG2-liver | COX6A2-muscle | -0.73636 | 0.00976 | 0.043199 |
| EPS8-liver | COX6A2-muscle | -0.71818 | 0.0128 | 0.048396 |
| EZR-liver | COX6A2-muscle | -0.7 | 0.016471 | 0.052234 |
| FNBP1L-liver | COX6A2-muscle | -0.71818 | 0.0128 | 0.048396 |
| HEG1-liver | COX6A2-muscle | -0.76364 | 0.006233 | 0.036383 |
| ITGB1-liver | COX6A2-muscle | -0.71818 | 0.0128 | 0.048396 |
| MMGT1-liver | COX6A2-muscle | -0.70909 | 0.014552 | 0.050131 |
| PLPP5-liver | COX6A2-muscle | -0.79091 | 0.003746 | 0.032221 |
| RAP2A-liver | COX6A2-muscle | -0.72727 | 0.011205 | 0.045908 |
| SMIM10-liver | COX6A2-muscle | -0.7 | 0.016471 | 0.052234 |
| SUCO-liver | COX6A2-muscle | -0.7 | 0.016471 | 0.052234 |
| SULF1-liver | COX6A2-muscle | -0.73636 | 0.00976 | 0.043199 |
| TMSB10-liver | COX6A2-muscle | -0.71818 | 0.0128 | 0.048396 |
| VCAN-liver | COX6A2-muscle | -0.73636 | 0.00976 | 0.043199 |
| YWHAZ-liver | COX6A2-muscle | -0.76364 | 0.006233 | 0.036383 |
| APP-liver | DLAT-muscle | -0.72727 | 0.011205 | 0.045908 |
| DSG2-liver | DLAT-muscle | -0.73636 | 0.00976 | 0.043199 |
| ITGB1-liver | DLAT-muscle | -0.7 | 0.016471 | 0.052234 |
| MMGT1-liver | DLAT-muscle | -0.73636 | 0.00976 | 0.043199 |
| NCKAP1-liver | DLAT-muscle | -0.74545 | 0.008455 | 0.041717 |
| PLPP5-liver | DLAT-muscle | -0.73636 | 0.00976 | 0.043199 |
| SLC25A6-liver | DLAT-muscle | -0.73636 | 0.00976 | 0.043199 |
| SUCO-liver | DLAT-muscle | -0.7 | 0.016471 | 0.052234 |
| ANXA5-liver | DNAJA2-muscle | -0.75455 | 0.007282 | 0.038865 |
| APP-liver | DNAJA2-muscle | -0.9 | 0.00016 | 0.016927 |
| ARHGAP18-liver | DNAJA2-muscle | -0.77273 | 0.005299 | 0.035326 |
| ARID5B-liver | DNAJA2-muscle | -0.70909 | 0.014552 | 0.050131 |
| BMPR2-liver | DNAJA2-muscle | -0.76364 | 0.006233 | 0.036383 |
| CA5B-liver | DNAJA2-muscle | -0.79091 | 0.003746 | 0.032221 |
| CD34-liver | DNAJA2-muscle | -0.80909 | 0.002559 | 0.029155 |
| CLIC1-liver | DNAJA2-muscle | -0.82727 | 0.001677 | 0.02618 |
| DEFB1-liver | DNAJA2-muscle | -0.74545 | 0.008455 | 0.041717 |
| DSG2-liver | DNAJA2-muscle | -0.83636 | 0.001333 | 0.025685 |
| FNBP1L-liver | DNAJA2-muscle | -0.80909 | 0.002559 | 0.029155 |
| HEG1-liver | DNAJA2-muscle | -0.75455 | 0.007282 | 0.038865 |
| ITGB1-liver | DNAJA2-muscle | -0.81818 | 0.002083 | 0.027717 |
| KHDRBS1-liver | DNAJA2-muscle | -0.78182 | 0.004473 | 0.033769 |
| KIF3A-liver | DNAJA2-muscle | -0.77273 | 0.005299 | 0.035326 |
| MFGE8-liver | DNAJA2-muscle | -0.70909 | 0.014552 | 0.050131 |
| MMGT1-liver | DNAJA2-muscle | -0.8 | 0.00311 | 0.031511 |
| MYH9-liver | DNAJA2-muscle | -0.74545 | 0.008455 | 0.041717 |
| NCKAP1-liver | DNAJA2-muscle | -0.80909 | 0.002559 | 0.029155 |
| PRKX-liver | DNAJA2-muscle | -0.79091 | 0.003746 | 0.032221 |
| RALB-liver | DNAJA2-muscle | -0.71818 | 0.0128 | 0.048396 |
| RAP2A-liver | DNAJA2-muscle | -0.84545 | 0.001045 | 0.024446 |
| SLC25A6-liver | DNAJA2-muscle | -0.79091 | 0.003746 | 0.032221 |
| SMIM10-liver | DNAJA2-muscle | -0.80909 | 0.002559 | 0.029155 |
| STX7-liver | DNAJA2-muscle | -0.80909 | 0.002559 | 0.029155 |
| SUCO-liver | DNAJA2-muscle | -0.79091 | 0.003746 | 0.032221 |
| SULF1-liver | DNAJA2-muscle | -0.82727 | 0.001677 | 0.02618 |
| TES-liver | DNAJA2-muscle | -0.79091 | 0.003746 | 0.032221 |
| TIMP1-liver | DNAJA2-muscle | -0.76364 | 0.006233 | 0.036383 |
| TMSB10-liver | DNAJA2-muscle | -0.79091 | 0.003746 | 0.032221 |
| TPM4-liver | DNAJA2-muscle | -0.8 | 0.00311 | 0.031511 |
| TTF2-liver | DNAJA2-muscle | -0.75455 | 0.007282 | 0.038865 |
| VCAN-liver | DNAJA2-muscle | -0.7 | 0.016471 | 0.052234 |
| YWHAZ-liver | DNAJA2-muscle | -0.87273 | 0.000455 | 0.020203 |
| ANXA5-liver | DNAJA3-muscle | -0.79091 | 0.003746 | 0.032221 |
| APP-liver | DNAJA3-muscle | -0.90909 | 0.000106 | 0.016759 |
| ARHGAP18-liver | DNAJA3-muscle | -0.79091 | 0.003746 | 0.032221 |
| ARID5B-liver | DNAJA3-muscle | -0.76364 | 0.006233 | 0.036383 |
| BMPR2-liver | DNAJA3-muscle | -0.75455 | 0.007282 | 0.038865 |
| CA5B-liver | DNAJA3-muscle | -0.78182 | 0.004473 | 0.033769 |
| CCN4-liver | DNAJA3-muscle | -0.70909 | 0.014552 | 0.050131 |
| CD34-liver | DNAJA3-muscle | -0.85455 | 0.000807 | 0.022594 |
| CLIC1-liver | DNAJA3-muscle | -0.86364 | 0.000612 | 0.022041 |
| DEFB1-liver | DNAJA3-muscle | -0.73636 | 0.00976 | 0.043199 |
| DSG2-liver | DNAJA3-muscle | -0.84545 | 0.001045 | 0.024446 |
| EPS8-liver | DNAJA3-muscle | -0.7 | 0.016471 | 0.052234 |
| EZR-liver | DNAJA3-muscle | -0.71818 | 0.0128 | 0.048396 |
| FNBP1L-liver | DNAJA3-muscle | -0.82727 | 0.001677 | 0.02618 |
| HEG1-liver | DNAJA3-muscle | -0.79091 | 0.003746 | 0.032221 |
| IFT57-liver | DNAJA3-muscle | -0.70909 | 0.014552 | 0.050131 |
| ITGB1-liver | DNAJA3-muscle | -0.82727 | 0.001677 | 0.02618 |
| KHDRBS1-liver | DNAJA3-muscle | -0.79091 | 0.003746 | 0.032221 |
| KIF3A-liver | DNAJA3-muscle | -0.79091 | 0.003746 | 0.032221 |
| KIF5B-liver | DNAJA3-muscle | -0.7 | 0.016471 | 0.052234 |
| MFGE8-liver | DNAJA3-muscle | -0.75455 | 0.007282 | 0.038865 |
| MFSD6-liver | DNAJA3-muscle | -0.7 | 0.016471 | 0.052234 |
| MMGT1-liver | DNAJA3-muscle | -0.80909 | 0.002559 | 0.029155 |
| MYH9-liver | DNAJA3-muscle | -0.76364 | 0.006233 | 0.036383 |
| NCKAP1-liver | DNAJA3-muscle | -0.8 | 0.00311 | 0.031511 |
| PLPP5-liver | DNAJA3-muscle | -0.7 | 0.016471 | 0.052234 |
| PRKX-liver | DNAJA3-muscle | -0.80909 | 0.002559 | 0.029155 |
| PRRC2C-liver | DNAJA3-muscle | -0.7 | 0.016471 | 0.052234 |
| RALB-liver | DNAJA3-muscle | -0.72727 | 0.011205 | 0.045908 |
| RAP2A-liver | DNAJA3-muscle | -0.87273 | 0.000455 | 0.020203 |
| RPS6KA2-liver | DNAJA3-muscle | -0.70909 | 0.014552 | 0.050131 |
| SLC25A6-liver | DNAJA3-muscle | -0.8 | 0.00311 | 0.031511 |
| SMIM10-liver | DNAJA3-muscle | -0.83636 | 0.001333 | 0.025685 |
| STX7-liver | DNAJA3-muscle | -0.84545 | 0.001045 | 0.024446 |
| SUCO-liver | DNAJA3-muscle | -0.80909 | 0.002559 | 0.029155 |
| SULF1-liver | DNAJA3-muscle | -0.86364 | 0.000612 | 0.022041 |
| TES-liver | DNAJA3-muscle | -0.80909 | 0.002559 | 0.029155 |
| TIMP1-liver | DNAJA3-muscle | -0.80909 | 0.002559 | 0.029155 |
| TMSB10-liver | DNAJA3-muscle | -0.81818 | 0.002083 | 0.027717 |
| TPM4-liver | DNAJA3-muscle | -0.80909 | 0.002559 | 0.029155 |
| TSPAN14-liver | DNAJA3-muscle | -0.7 | 0.016471 | 0.052234 |
| TTF2-liver | DNAJA3-muscle | -0.7 | 0.016471 | 0.052234 |
| VCAN-liver | DNAJA3-muscle | -0.73636 | 0.00976 | 0.043199 |
| YWHAZ-liver | DNAJA3-muscle | -0.90909 | 0.000106 | 0.016759 |
| APP-liver | ECI1-muscle | -0.81818 | 0.002083 | 0.027717 |
| CLIC1-liver | ECI1-muscle | -0.7 | 0.016471 | 0.052234 |
| DSG2-liver | ECI1-muscle | -0.72727 | 0.011205 | 0.045908 |
| ITGB1-liver | ECI1-muscle | -0.71818 | 0.0128 | 0.048396 |
| NCKAP1-liver | ECI1-muscle | -0.7 | 0.016471 | 0.052234 |
| RAP2A-liver | ECI1-muscle | -0.70909 | 0.014552 | 0.050131 |
| SULF1-liver | ECI1-muscle | -0.7 | 0.016471 | 0.052234 |
| YWHAZ-liver | ECI1-muscle | -0.76364 | 0.006233 | 0.036383 |
| ANXA5-liver | ENDOG-muscle | -0.79091 | 0.003746 | 0.032221 |
| APP-liver | ENDOG-muscle | -0.83636 | 0.001333 | 0.025685 |
| ARHGAP18-liver | ENDOG-muscle | -0.76364 | 0.006233 | 0.036383 |
| ARID5B-liver | ENDOG-muscle | -0.79091 | 0.003746 | 0.032221 |
| CCN4-liver | ENDOG-muscle | -0.7 | 0.016471 | 0.052234 |
| CD34-liver | ENDOG-muscle | -0.81818 | 0.002083 | 0.027717 |
| CDH5-liver | ENDOG-muscle | -0.77273 | 0.005299 | 0.035326 |
| CLIC1-liver | ENDOG-muscle | -0.80909 | 0.002559 | 0.029155 |
| DEFB1-liver | ENDOG-muscle | -0.7 | 0.016471 | 0.052234 |
| DSG2-liver | ENDOG-muscle | -0.78182 | 0.004473 | 0.033769 |
| EZR-liver | ENDOG-muscle | -0.70909 | 0.014552 | 0.050131 |
| FNBP1L-liver | ENDOG-muscle | -0.76364 | 0.006233 | 0.036383 |
| HEG1-liver | ENDOG-muscle | -0.76364 | 0.006233 | 0.036383 |
| IFT57-liver | ENDOG-muscle | -0.73636 | 0.00976 | 0.043199 |
| ITGB1-liver | ENDOG-muscle | -0.74545 | 0.008455 | 0.041717 |
| KHDRBS1-liver | ENDOG-muscle | -0.79091 | 0.003746 | 0.032221 |
| KIF3A-liver | ENDOG-muscle | -0.75455 | 0.007282 | 0.038865 |
| MFGE8-liver | ENDOG-muscle | -0.79091 | 0.003746 | 0.032221 |
| MMGT1-liver | ENDOG-muscle | -0.78182 | 0.004473 | 0.033769 |
| MYH9-liver | ENDOG-muscle | -0.75455 | 0.007282 | 0.038865 |
| NCKAP1-liver | ENDOG-muscle | -0.74545 | 0.008455 | 0.041717 |
| PRKX-liver | ENDOG-muscle | -0.78182 | 0.004473 | 0.033769 |
| RAP2A-liver | ENDOG-muscle | -0.80909 | 0.002559 | 0.029155 |
| SLC25A6-liver | ENDOG-muscle | -0.78182 | 0.004473 | 0.033769 |
| SMIM10-liver | ENDOG-muscle | -0.75455 | 0.007282 | 0.038865 |
| STX7-liver | ENDOG-muscle | -0.82727 | 0.001677 | 0.02618 |
| SUCO-liver | ENDOG-muscle | -0.78182 | 0.004473 | 0.033769 |
| SULF1-liver | ENDOG-muscle | -0.80909 | 0.002559 | 0.029155 |
| TES-liver | ENDOG-muscle | -0.77273 | 0.005299 | 0.035326 |
| TIMP1-liver | ENDOG-muscle | -0.81818 | 0.002083 | 0.027717 |
| TMSB10-liver | ENDOG-muscle | -0.77273 | 0.005299 | 0.035326 |
| TPM4-liver | ENDOG-muscle | -0.72727 | 0.011205 | 0.045908 |
| TSPAN14-liver | ENDOG-muscle | -0.70909 | 0.014552 | 0.050131 |
| VCAN-liver | ENDOG-muscle | -0.73636 | 0.00976 | 0.043199 |
| YWHAZ-liver | ENDOG-muscle | -0.84545 | 0.001045 | 0.024446 |
| BCL2L11-liver | ERCC8-muscle | -0.72727 | 0.011205 | 0.045908 |
| ADGRL4-liver | FAM210A-muscle | -0.76364 | 0.006233 | 0.036383 |
| ALCAM-liver | FAM210A-muscle | -0.71818 | 0.0128 | 0.048396 |
| ANXA5-liver | FAM210A-muscle | -0.73636 | 0.00976 | 0.043199 |
| APP-liver | FAM210A-muscle | -0.81818 | 0.002083 | 0.027717 |
| ARHGAP18-liver | FAM210A-muscle | -0.80909 | 0.002559 | 0.029155 |
| ARID5B-liver | FAM210A-muscle | -0.74545 | 0.008455 | 0.041717 |
| BMPR2-liver | FAM210A-muscle | -0.75455 | 0.007282 | 0.038865 |
| CCN4-liver | FAM210A-muscle | -0.75455 | 0.007282 | 0.038865 |
| CDH5-liver | FAM210A-muscle | -0.75455 | 0.007282 | 0.038865 |
| CLIC1-liver | FAM210A-muscle | -0.71818 | 0.0128 | 0.048396 |
| DEFB1-liver | FAM210A-muscle | -0.82727 | 0.001677 | 0.02618 |
| DSG2-liver | FAM210A-muscle | -0.81818 | 0.002083 | 0.027717 |
| EPS8-liver | FAM210A-muscle | -0.74545 | 0.008455 | 0.041717 |
| EZR-liver | FAM210A-muscle | -0.7 | 0.016471 | 0.052234 |
| FNBP1L-liver | FAM210A-muscle | -0.76364 | 0.006233 | 0.036383 |
| HDAC2-liver | FAM210A-muscle | -0.73636 | 0.00976 | 0.043199 |
| HEG1-liver | FAM210A-muscle | -0.79091 | 0.003746 | 0.032221 |
| IFT57-liver | FAM210A-muscle | -0.84545 | 0.001045 | 0.024446 |
| ITGB1-liver | FAM210A-muscle | -0.77273 | 0.005299 | 0.035326 |
| KHDRBS1-liver | FAM210A-muscle | -0.79091 | 0.003746 | 0.032221 |
| KIF3A-liver | FAM210A-muscle | -0.77273 | 0.005299 | 0.035326 |
| MFGE8-liver | FAM210A-muscle | -0.78182 | 0.004473 | 0.033769 |
| MMGT1-liver | FAM210A-muscle | -0.86364 | 0.000612 | 0.022041 |
| MYH9-liver | FAM210A-muscle | -0.79091 | 0.003746 | 0.032221 |
| NCKAP1-liver | FAM210A-muscle | -0.8 | 0.00311 | 0.031511 |
| PLPP5-liver | FAM210A-muscle | -0.76364 | 0.006233 | 0.036383 |
| PRKX-liver | FAM210A-muscle | -0.72727 | 0.011205 | 0.045908 |
| RAP2A-liver | FAM210A-muscle | -0.74545 | 0.008455 | 0.041717 |
| SLC25A6-liver | FAM210A-muscle | -0.80909 | 0.002559 | 0.029155 |
| SMIM10-liver | FAM210A-muscle | -0.70909 | 0.014552 | 0.050131 |
| STX7-liver | FAM210A-muscle | -0.70909 | 0.014552 | 0.050131 |
| SUCO-liver | FAM210A-muscle | -0.8 | 0.00311 | 0.031511 |
| SULF1-liver | FAM210A-muscle | -0.71818 | 0.0128 | 0.048396 |
| TES-liver | FAM210A-muscle | -0.76364 | 0.006233 | 0.036383 |
| TMSB10-liver | FAM210A-muscle | -0.77273 | 0.005299 | 0.035326 |
| TPM4-liver | FAM210A-muscle | -0.78182 | 0.004473 | 0.033769 |
| TSPAN14-liver | FAM210A-muscle | -0.8 | 0.00311 | 0.031511 |
| VCAN-liver | FAM210A-muscle | -0.80909 | 0.002559 | 0.029155 |
| YWHAZ-liver | FAM210A-muscle | -0.72727 | 0.011205 | 0.045908 |
| DEFB1-liver | FYCO1-muscle | -0.72727 | 0.011205 | 0.045908 |
| IFT57-liver | FYCO1-muscle | -0.7 | 0.016471 | 0.052234 |
| MMGT1-liver | FYCO1-muscle | -0.7 | 0.016471 | 0.052234 |
| ARID5B-liver | GHR-muscle | -0.70909 | 0.014552 | 0.050131 |
| MFGE8-liver | GHR-muscle | -0.7 | 0.016471 | 0.052234 |
| YWHAZ-liver | GHR-muscle | -0.7 | 0.016471 | 0.052234 |
| ABI2-liver | GPT2-muscle | -0.7 | 0.016471 | 0.052234 |
| ADGRL4-liver | GPT2-muscle | -0.86364 | 0.000612 | 0.022041 |
| ALCAM-liver | GPT2-muscle | -0.78182 | 0.004473 | 0.033769 |
| ANXA5-liver | GPT2-muscle | -0.8 | 0.00311 | 0.031511 |
| AP2B1-liver | GPT2-muscle | -0.70909 | 0.014552 | 0.050131 |
| APP-liver | GPT2-muscle | -0.9 | 0.00016 | 0.016927 |
| ARHGAP18-liver | GPT2-muscle | -0.87273 | 0.000455 | 0.020203 |
| ARID5B-liver | GPT2-muscle | -0.77273 | 0.005299 | 0.035326 |
| B4GALT5-liver | GPT2-muscle | -0.78182 | 0.004473 | 0.033769 |
| BMPR2-liver | GPT2-muscle | -0.81818 | 0.002083 | 0.027717 |
| CA5B-liver | GPT2-muscle | -0.72727 | 0.011205 | 0.045908 |
| CCN4-liver | GPT2-muscle | -0.84545 | 0.001045 | 0.024446 |
| CD34-liver | GPT2-muscle | -0.75455 | 0.007282 | 0.038865 |
| CDH5-liver | GPT2-muscle | -0.83636 | 0.001333 | 0.025685 |
| CLIC1-liver | GPT2-muscle | -0.78182 | 0.004473 | 0.033769 |
| DEFB1-liver | GPT2-muscle | -0.88182 | 0.00033 | 0.018341 |
| DSG2-liver | GPT2-muscle | -0.90909 | 0.000106 | 0.016759 |
| EPS8-liver | GPT2-muscle | -0.81818 | 0.002083 | 0.027717 |
| EZR-liver | GPT2-muscle | -0.76364 | 0.006233 | 0.036383 |
| FNBP1L-liver | GPT2-muscle | -0.86364 | 0.000612 | 0.022041 |
| HDAC2-liver | GPT2-muscle | -0.8 | 0.00311 | 0.031511 |
| HEG1-liver | GPT2-muscle | -0.79091 | 0.003746 | 0.032221 |
| IFT57-liver | GPT2-muscle | -0.82727 | 0.001677 | 0.02618 |
| IPO7-liver | GPT2-muscle | -0.80909 | 0.002559 | 0.029155 |
| ITGB1-liver | GPT2-muscle | -0.88182 | 0.00033 | 0.018341 |
| KHDRBS1-liver | GPT2-muscle | -0.86364 | 0.000612 | 0.022041 |
| KIF3A-liver | GPT2-muscle | -0.83636 | 0.001333 | 0.025685 |
| KIF5B-liver | GPT2-muscle | -0.73636 | 0.00976 | 0.043199 |
| MFGE8-liver | GPT2-muscle | -0.82727 | 0.001677 | 0.02618 |
| MFSD6-liver | GPT2-muscle | -0.76364 | 0.006233 | 0.036383 |
| MKKS-liver | GPT2-muscle | -0.70909 | 0.014552 | 0.050131 |
| MMGT1-liver | GPT2-muscle | -0.91818 | 6.66E-05 | 0.016759 |
| MTMR6-liver | GPT2-muscle | -0.73636 | 0.00976 | 0.043199 |
| MYH9-liver | GPT2-muscle | -0.85455 | 0.000807 | 0.022594 |
| NCKAP1-liver | GPT2-muscle | -0.89091 | 0.000233 | 0.018341 |
| PLPP5-liver | GPT2-muscle | -0.81818 | 0.002083 | 0.027717 |
| PRKX-liver | GPT2-muscle | -0.80909 | 0.002559 | 0.029155 |
| PRRC2C-liver | GPT2-muscle | -0.8 | 0.00311 | 0.031511 |
| RALB-liver | GPT2-muscle | -0.80909 | 0.002559 | 0.029155 |
| RAP2A-liver | GPT2-muscle | -0.80909 | 0.002559 | 0.029155 |
| RPS6KA2-liver | GPT2-muscle | -0.79091 | 0.003746 | 0.032221 |
| SLC25A6-liver | GPT2-muscle | -0.90909 | 0.000106 | 0.016759 |
| SMIM10-liver | GPT2-muscle | -0.76364 | 0.006233 | 0.036383 |
| STX7-liver | GPT2-muscle | -0.80909 | 0.002559 | 0.029155 |
| SUCO-liver | GPT2-muscle | -0.89091 | 0.000233 | 0.018341 |
| SULF1-liver | GPT2-muscle | -0.78182 | 0.004473 | 0.033769 |
| TES-liver | GPT2-muscle | -0.85455 | 0.000807 | 0.022594 |
| TIMP1-liver | GPT2-muscle | -0.75455 | 0.007282 | 0.038865 |
| TMSB10-liver | GPT2-muscle | -0.8 | 0.00311 | 0.031511 |
| TPM4-liver | GPT2-muscle | -0.82727 | 0.001677 | 0.02618 |
| TSPAN14-liver | GPT2-muscle | -0.79091 | 0.003746 | 0.032221 |
| TTF2-liver | GPT2-muscle | -0.82727 | 0.001677 | 0.02618 |
| VCAN-liver | GPT2-muscle | -0.81818 | 0.002083 | 0.027717 |
| VEZF1-liver | GPT2-muscle | -0.7 | 0.016471 | 0.052234 |
| WDR1-liver | GPT2-muscle | -0.71818 | 0.0128 | 0.048396 |
| YWHAZ-liver | GPT2-muscle | -0.79091 | 0.003746 | 0.032221 |
| ADGRL4-liver | GTF2H5-muscle | -0.70909 | 0.014552 | 0.050131 |
| ANKRD27-liver | GTF2H5-muscle | -0.71818 | 0.0128 | 0.048396 |
| ANXA5-liver | GTF2H5-muscle | -0.71818 | 0.0128 | 0.048396 |
| APP-liver | GTF2H5-muscle | -0.85455 | 0.000807 | 0.022594 |
| ARHGAP18-liver | GTF2H5-muscle | -0.77273 | 0.005299 | 0.035326 |
| ARID5B-liver | GTF2H5-muscle | -0.73636 | 0.00976 | 0.043199 |
| B4GALT5-liver | GTF2H5-muscle | -0.73636 | 0.00976 | 0.043199 |
| BMPR2-liver | GTF2H5-muscle | -0.79091 | 0.003746 | 0.032221 |
| CA5B-liver | GTF2H5-muscle | -0.77273 | 0.005299 | 0.035326 |
| CCN4-liver | GTF2H5-muscle | -0.79091 | 0.003746 | 0.032221 |
| CD34-liver | GTF2H5-muscle | -0.77273 | 0.005299 | 0.035326 |
| CDH5-liver | GTF2H5-muscle | -0.72727 | 0.011205 | 0.045908 |
| CLIC1-liver | GTF2H5-muscle | -0.80909 | 0.002559 | 0.029155 |
| DSG2-liver | GTF2H5-muscle | -0.86364 | 0.000612 | 0.022041 |
| EPS8-liver | GTF2H5-muscle | -0.82727 | 0.001677 | 0.02618 |
| EZR-liver | GTF2H5-muscle | -0.83636 | 0.001333 | 0.025685 |
| FNBP1L-liver | GTF2H5-muscle | -0.84545 | 0.001045 | 0.024446 |
| HEG1-liver | GTF2H5-muscle | -0.79091 | 0.003746 | 0.032221 |
| IPO7-liver | GTF2H5-muscle | -0.77273 | 0.005299 | 0.035326 |
| ITGB1-liver | GTF2H5-muscle | -0.87273 | 0.000455 | 0.020203 |
| KHDRBS1-liver | GTF2H5-muscle | -0.76364 | 0.006233 | 0.036383 |
| KIF3A-liver | GTF2H5-muscle | -0.72727 | 0.011205 | 0.045908 |
| KIF5B-liver | GTF2H5-muscle | -0.76364 | 0.006233 | 0.036383 |
| MFGE8-liver | GTF2H5-muscle | -0.76364 | 0.006233 | 0.036383 |
| MMGT1-liver | GTF2H5-muscle | -0.79091 | 0.003746 | 0.032221 |
| MYH9-liver | GTF2H5-muscle | -0.73636 | 0.00976 | 0.043199 |
| NCKAP1-liver | GTF2H5-muscle | -0.82727 | 0.001677 | 0.02618 |
| PLPP5-liver | GTF2H5-muscle | -0.88182 | 0.00033 | 0.018341 |
| PRKX-liver | GTF2H5-muscle | -0.79091 | 0.003746 | 0.032221 |
| PRRC2C-liver | GTF2H5-muscle | -0.76364 | 0.006233 | 0.036383 |
| RAP2A-liver | GTF2H5-muscle | -0.79091 | 0.003746 | 0.032221 |
| RPS6KA2-liver | GTF2H5-muscle | -0.75455 | 0.007282 | 0.038865 |
| SLC25A6-liver | GTF2H5-muscle | -0.82727 | 0.001677 | 0.02618 |
| SMIM10-liver | GTF2H5-muscle | -0.76364 | 0.006233 | 0.036383 |
| STX7-liver | GTF2H5-muscle | -0.74545 | 0.008455 | 0.041717 |
| SUCO-liver | GTF2H5-muscle | -0.80909 | 0.002559 | 0.029155 |
| SULF1-liver | GTF2H5-muscle | -0.80909 | 0.002559 | 0.029155 |
| TES-liver | GTF2H5-muscle | -0.76364 | 0.006233 | 0.036383 |
| TIMP1-liver | GTF2H5-muscle | -0.73636 | 0.00976 | 0.043199 |
| TMSB10-liver | GTF2H5-muscle | -0.76364 | 0.006233 | 0.036383 |
| TPM4-liver | GTF2H5-muscle | -0.76364 | 0.006233 | 0.036383 |
| TTF2-liver | GTF2H5-muscle | -0.70909 | 0.014552 | 0.050131 |
| VCAN-liver | GTF2H5-muscle | -0.78182 | 0.004473 | 0.033769 |
| YWHAZ-liver | GTF2H5-muscle | -0.83636 | 0.001333 | 0.025685 |
| APP-liver | GTPBP8-muscle | -0.79091 | 0.003746 | 0.032221 |
| ARHGAP18-liver | GTPBP8-muscle | -0.75455 | 0.007282 | 0.038865 |
| BMPR2-liver | GTPBP8-muscle | -0.75455 | 0.007282 | 0.038865 |
| DEFB1-liver | GTPBP8-muscle | -0.71818 | 0.0128 | 0.048396 |
| DSG2-liver | GTPBP8-muscle | -0.79091 | 0.003746 | 0.032221 |
| EPS8-liver | GTPBP8-muscle | -0.7 | 0.016471 | 0.052234 |
| EZR-liver | GTPBP8-muscle | -0.71818 | 0.0128 | 0.048396 |
| FNBP1L-liver | GTPBP8-muscle | -0.74545 | 0.008455 | 0.041717 |
| HEG1-liver | GTPBP8-muscle | -0.70909 | 0.014552 | 0.050131 |
| ITGB1-liver | GTPBP8-muscle | -0.74545 | 0.008455 | 0.041717 |
| KHDRBS1-liver | GTPBP8-muscle | -0.72727 | 0.011205 | 0.045908 |
| KIF3A-liver | GTPBP8-muscle | -0.7 | 0.016471 | 0.052234 |
| KIF5B-liver | GTPBP8-muscle | -0.70909 | 0.014552 | 0.050131 |
| MMGT1-liver | GTPBP8-muscle | -0.8 | 0.00311 | 0.031511 |
| NCKAP1-liver | GTPBP8-muscle | -0.80909 | 0.002559 | 0.029155 |
| PLPP5-liver | GTPBP8-muscle | -0.75455 | 0.007282 | 0.038865 |
| PRKX-liver | GTPBP8-muscle | -0.70909 | 0.014552 | 0.050131 |
| RAP2A-liver | GTPBP8-muscle | -0.73636 | 0.00976 | 0.043199 |
| SLC25A6-liver | GTPBP8-muscle | -0.75455 | 0.007282 | 0.038865 |
| SMIM10-liver | GTPBP8-muscle | -0.70909 | 0.014552 | 0.050131 |
| SUCO-liver | GTPBP8-muscle | -0.73636 | 0.00976 | 0.043199 |
| TMEM138-liver | GTPBP8-muscle | -0.70909 | 0.014552 | 0.050131 |
| TMSB10-liver | GTPBP8-muscle | -0.7 | 0.016471 | 0.052234 |
| TPM4-liver | GTPBP8-muscle | -0.74545 | 0.008455 | 0.041717 |
| TTF2-liver | GTPBP8-muscle | -0.73636 | 0.00976 | 0.043199 |
| VCAN-liver | GTPBP8-muscle | -0.71818 | 0.0128 | 0.048396 |
| YWHAZ-liver | GTPBP8-muscle | -0.70909 | 0.014552 | 0.050131 |
| ABI2-liver | H2AZ2-muscle | -0.7 | 0.016471 | 0.052234 |
| ADGRL4-liver | H2AZ2-muscle | -0.72727 | 0.011205 | 0.045908 |
| ANXA5-liver | H2AZ2-muscle | -0.81818 | 0.002083 | 0.027717 |
| APP-liver | H2AZ2-muscle | -0.79091 | 0.003746 | 0.032221 |
| ARHGAP18-liver | H2AZ2-muscle | -0.81818 | 0.002083 | 0.027717 |
| ARID5B-liver | H2AZ2-muscle | -0.9 | 0.00016 | 0.016927 |
| CAMSAP2-liver | H2AZ2-muscle | -0.70909 | 0.014552 | 0.050131 |
| CCN4-liver | H2AZ2-muscle | -0.79091 | 0.003746 | 0.032221 |
| CD34-liver | H2AZ2-muscle | -0.80909 | 0.002559 | 0.029155 |
| CDH5-liver | H2AZ2-muscle | -0.78182 | 0.004473 | 0.033769 |
| CLIC1-liver | H2AZ2-muscle | -0.78182 | 0.004473 | 0.033769 |
| DEFB1-liver | H2AZ2-muscle | -0.72727 | 0.011205 | 0.045908 |
| DSG2-liver | H2AZ2-muscle | -0.79091 | 0.003746 | 0.032221 |
| EPS8-liver | H2AZ2-muscle | -0.79091 | 0.003746 | 0.032221 |
| EZR-liver | H2AZ2-muscle | -0.70909 | 0.014552 | 0.050131 |
| FNBP1L-liver | H2AZ2-muscle | -0.78182 | 0.004473 | 0.033769 |
| HEG1-liver | H2AZ2-muscle | -0.79091 | 0.003746 | 0.032221 |
| IFT57-liver | H2AZ2-muscle | -0.80909 | 0.002559 | 0.029155 |
| ITGB1-liver | H2AZ2-muscle | -0.73636 | 0.00976 | 0.043199 |
| KHDRBS1-liver | H2AZ2-muscle | -0.76364 | 0.006233 | 0.036383 |
| KIF3A-liver | H2AZ2-muscle | -0.77273 | 0.005299 | 0.035326 |
| MFGE8-liver | H2AZ2-muscle | -0.9 | 0.00016 | 0.016927 |
| MFSD6-liver | H2AZ2-muscle | -0.73636 | 0.00976 | 0.043199 |
| MMGT1-liver | H2AZ2-muscle | -0.82727 | 0.001677 | 0.02618 |
| MYH9-liver | H2AZ2-muscle | -0.76364 | 0.006233 | 0.036383 |
| NCKAP1-liver | H2AZ2-muscle | -0.71818 | 0.0128 | 0.048396 |
| PLPP5-liver | H2AZ2-muscle | -0.75455 | 0.007282 | 0.038865 |
| PRKX-liver | H2AZ2-muscle | -0.73636 | 0.00976 | 0.043199 |
| PRRC2C-liver | H2AZ2-muscle | -0.70909 | 0.014552 | 0.050131 |
| RAP2A-liver | H2AZ2-muscle | -0.79091 | 0.003746 | 0.032221 |
| RPS6KA2-liver | H2AZ2-muscle | -0.70909 | 0.014552 | 0.050131 |
| SLC25A6-liver | H2AZ2-muscle | -0.8 | 0.00311 | 0.031511 |
| SMIM10-liver | H2AZ2-muscle | -0.72727 | 0.011205 | 0.045908 |
| STX7-liver | H2AZ2-muscle | -0.84545 | 0.001045 | 0.024446 |
| SUCO-liver | H2AZ2-muscle | -0.82727 | 0.001677 | 0.02618 |
| SULF1-liver | H2AZ2-muscle | -0.78182 | 0.004473 | 0.033769 |
| TES-liver | H2AZ2-muscle | -0.78182 | 0.004473 | 0.033769 |
| TIMP1-liver | H2AZ2-muscle | -0.81818 | 0.002083 | 0.027717 |
| TMSB10-liver | H2AZ2-muscle | -0.76364 | 0.006233 | 0.036383 |
| TPM4-liver | H2AZ2-muscle | -0.7 | 0.016471 | 0.052234 |
| TSPAN14-liver | H2AZ2-muscle | -0.72727 | 0.011205 | 0.045908 |
| VCAN-liver | H2AZ2-muscle | -0.82727 | 0.001677 | 0.02618 |
| YWHAZ-liver | H2AZ2-muscle | -0.8 | 0.00311 | 0.031511 |
| ANXA5-liver | HCCS-muscle | -0.7 | 0.016471 | 0.052234 |
| APP-liver | HCCS-muscle | -0.70909 | 0.014552 | 0.050131 |
| ARHGAP18-liver | HCCS-muscle | -0.71818 | 0.0128 | 0.048396 |
| ARID5B-liver | HCCS-muscle | -0.81818 | 0.002083 | 0.027717 |
| CD34-liver | HCCS-muscle | -0.70909 | 0.014552 | 0.050131 |
| DSG2-liver | HCCS-muscle | -0.7 | 0.016471 | 0.052234 |
| IFT57-liver | HCCS-muscle | -0.70909 | 0.014552 | 0.050131 |
| MFGE8-liver | HCCS-muscle | -0.80909 | 0.002559 | 0.029155 |
| MMGT1-liver | HCCS-muscle | -0.73636 | 0.00976 | 0.043199 |
| RAP2A-liver | HCCS-muscle | -0.7 | 0.016471 | 0.052234 |
| SLC25A6-liver | HCCS-muscle | -0.7 | 0.016471 | 0.052234 |
| STX7-liver | HCCS-muscle | -0.74545 | 0.008455 | 0.041717 |
| SUCO-liver | HCCS-muscle | -0.72727 | 0.011205 | 0.045908 |
| TIMP1-liver | HCCS-muscle | -0.70909 | 0.014552 | 0.050131 |
| VCAN-liver | HCCS-muscle | -0.72727 | 0.011205 | 0.045908 |
| YWHAZ-liver | HCCS-muscle | -0.71818 | 0.0128 | 0.048396 |
| APP-liver | HINT3-muscle | -0.76364 | 0.006233 | 0.036383 |
| DSG2-liver | HINT3-muscle | -0.72727 | 0.011205 | 0.045908 |
| MMGT1-liver | HINT3-muscle | -0.70909 | 0.014552 | 0.050131 |
| NCKAP1-liver | HINT3-muscle | -0.72727 | 0.011205 | 0.045908 |
| RAP2A-liver | HINT3-muscle | -0.70909 | 0.014552 | 0.050131 |
| TTF2-liver | HINT3-muscle | -0.78182 | 0.004473 | 0.033769 |
| YWHAZ-liver | HINT3-muscle | -0.70909 | 0.014552 | 0.050131 |
| ALCAM-liver | ITGB1BP2-muscle | -0.70909 | 0.014552 | 0.050131 |
| APP-liver | ITGB1BP2-muscle | -0.72727 | 0.011205 | 0.045908 |
| DEFB1-liver | ITGB1BP2-muscle | -0.72727 | 0.011205 | 0.045908 |
| DSG2-liver | ITGB1BP2-muscle | -0.71818 | 0.0128 | 0.048396 |
| MMGT1-liver | ITGB1BP2-muscle | -0.73636 | 0.00976 | 0.043199 |
| NCKAP1-liver | ITGB1BP2-muscle | -0.70909 | 0.014552 | 0.050131 |
| SLC25A6-liver | ITGB1BP2-muscle | -0.72727 | 0.011205 | 0.045908 |
| SUCO-liver | ITGB1BP2-muscle | -0.7 | 0.016471 | 0.052234 |
| TTF2-liver | ITGB1BP2-muscle | -0.75455 | 0.007282 | 0.038865 |
| IPO7-liver | KLHDC1-muscle | -0.73636 | 0.00976 | 0.043199 |
| TTF2-liver | KLHDC1-muscle | -0.84545 | 0.001045 | 0.024446 |
| PLPP5-liver | KREMEN1-muscle | -0.73636 | 0.00976 | 0.043199 |
| APP-liver | LACTB2-muscle | -0.75455 | 0.007282 | 0.038865 |
| CA5B-liver | LACTB2-muscle | -0.71818 | 0.0128 | 0.048396 |
| DSG2-liver | LACTB2-muscle | -0.71818 | 0.0128 | 0.048396 |
| FNBP1L-liver | LACTB2-muscle | -0.7 | 0.016471 | 0.052234 |
| IPO7-liver | LACTB2-muscle | -0.7 | 0.016471 | 0.052234 |
| KHDRBS1-liver | LACTB2-muscle | -0.70909 | 0.014552 | 0.050131 |
| MMGT1-liver | LACTB2-muscle | -0.70909 | 0.014552 | 0.050131 |
| NCKAP1-liver | LACTB2-muscle | -0.75455 | 0.007282 | 0.038865 |
| PRKX-liver | LACTB2-muscle | -0.70909 | 0.014552 | 0.050131 |
| RAP2A-liver | LACTB2-muscle | -0.72727 | 0.011205 | 0.045908 |
| SLC25A6-liver | LACTB2-muscle | -0.70909 | 0.014552 | 0.050131 |
| STX7-liver | LACTB2-muscle | -0.71818 | 0.0128 | 0.048396 |
| SUCO-liver | LACTB2-muscle | -0.70909 | 0.014552 | 0.050131 |
| TTF2-liver | LACTB2-muscle | -0.79091 | 0.003746 | 0.032221 |
| APP-liver | LIAS-muscle | -0.76364 | 0.006233 | 0.036383 |
| BMPR2-liver | LIAS-muscle | -0.70909 | 0.014552 | 0.050131 |
| DSG2-liver | LIAS-muscle | -0.74545 | 0.008455 | 0.041717 |
| IFT57-liver | LIAS-muscle | -0.7 | 0.016471 | 0.052234 |
| ITGB1-liver | LIAS-muscle | -0.71818 | 0.0128 | 0.048396 |
| MMGT1-liver | LIAS-muscle | -0.75455 | 0.007282 | 0.038865 |
| NCKAP1-liver | LIAS-muscle | -0.72727 | 0.011205 | 0.045908 |
| PLPP5-liver | LIAS-muscle | -0.70909 | 0.014552 | 0.050131 |
| SLC25A6-liver | LIAS-muscle | -0.71818 | 0.0128 | 0.048396 |
| SUCO-liver | LIAS-muscle | -0.7 | 0.016471 | 0.052234 |
| ADGRL4-liver | LRPPRC-muscle | -0.76364 | 0.006233 | 0.036383 |
| ANXA5-liver | LRPPRC-muscle | -0.7 | 0.016471 | 0.052234 |
| APP-liver | LRPPRC-muscle | -0.88182 | 0.00033 | 0.018341 |
| ARHGAP18-liver | LRPPRC-muscle | -0.78182 | 0.004473 | 0.033769 |
| B4GALT5-liver | LRPPRC-muscle | -0.73636 | 0.00976 | 0.043199 |
| BMPR2-liver | LRPPRC-muscle | -0.80909 | 0.002559 | 0.029155 |
| CA5B-liver | LRPPRC-muscle | -0.75455 | 0.007282 | 0.038865 |
| CCN4-liver | LRPPRC-muscle | -0.74545 | 0.008455 | 0.041717 |
| CD34-liver | LRPPRC-muscle | -0.7 | 0.016471 | 0.052234 |
| CDH5-liver | LRPPRC-muscle | -0.71818 | 0.0128 | 0.048396 |
| CLIC1-liver | LRPPRC-muscle | -0.73636 | 0.00976 | 0.043199 |
| DEFB1-liver | LRPPRC-muscle | -0.79091 | 0.003746 | 0.032221 |
| DSG2-liver | LRPPRC-muscle | -0.86364 | 0.000612 | 0.022041 |
| EPS8-liver | LRPPRC-muscle | -0.71818 | 0.0128 | 0.048396 |
| EZR-liver | LRPPRC-muscle | -0.7 | 0.016471 | 0.052234 |
| FNBP1L-liver | LRPPRC-muscle | -0.80909 | 0.002559 | 0.029155 |
| HDAC2-liver | LRPPRC-muscle | -0.7 | 0.016471 | 0.052234 |
| HEG1-liver | LRPPRC-muscle | -0.70909 | 0.014552 | 0.050131 |
| IFT57-liver | LRPPRC-muscle | -0.70909 | 0.014552 | 0.050131 |
| IPO7-liver | LRPPRC-muscle | -0.80909 | 0.002559 | 0.029155 |
| ITGB1-liver | LRPPRC-muscle | -0.85455 | 0.000807 | 0.022594 |
| KHDRBS1-liver | LRPPRC-muscle | -0.78182 | 0.004473 | 0.033769 |
| KIF3A-liver | LRPPRC-muscle | -0.74545 | 0.008455 | 0.041717 |
| KIF5B-liver | LRPPRC-muscle | -0.70909 | 0.014552 | 0.050131 |
| MFGE8-liver | LRPPRC-muscle | -0.71818 | 0.0128 | 0.048396 |
| MMGT1-liver | LRPPRC-muscle | -0.83636 | 0.001333 | 0.025685 |
| MYH9-liver | LRPPRC-muscle | -0.76364 | 0.006233 | 0.036383 |
| NCKAP1-liver | LRPPRC-muscle | -0.84545 | 0.001045 | 0.024446 |
| PLPP5-liver | LRPPRC-muscle | -0.74545 | 0.008455 | 0.041717 |
| PRKX-liver | LRPPRC-muscle | -0.74545 | 0.008455 | 0.041717 |
| PRRC2C-liver | LRPPRC-muscle | -0.70909 | 0.014552 | 0.050131 |
| RALB-liver | LRPPRC-muscle | -0.71818 | 0.0128 | 0.048396 |
| RAP2A-liver | LRPPRC-muscle | -0.76364 | 0.006233 | 0.036383 |
| RPS6KA2-liver | LRPPRC-muscle | -0.72727 | 0.011205 | 0.045908 |
| SLC25A6-liver | LRPPRC-muscle | -0.83636 | 0.001333 | 0.025685 |
| SMIM10-liver | LRPPRC-muscle | -0.72727 | 0.011205 | 0.045908 |
| STX7-liver | LRPPRC-muscle | -0.73636 | 0.00976 | 0.043199 |
| SUCO-liver | LRPPRC-muscle | -0.80909 | 0.002559 | 0.029155 |
| SULF1-liver | LRPPRC-muscle | -0.73636 | 0.00976 | 0.043199 |
| TES-liver | LRPPRC-muscle | -0.77273 | 0.005299 | 0.035326 |
| TMSB10-liver | LRPPRC-muscle | -0.72727 | 0.011205 | 0.045908 |
| TPM4-liver | LRPPRC-muscle | -0.78182 | 0.004473 | 0.033769 |
| TTF2-liver | LRPPRC-muscle | -0.82727 | 0.001677 | 0.02618 |
| VCAN-liver | LRPPRC-muscle | -0.70909 | 0.014552 | 0.050131 |
| YWHAZ-liver | LRPPRC-muscle | -0.77273 | 0.005299 | 0.035326 |
| APP-liver | LYRM7-muscle | -0.77273 | 0.005299 | 0.035326 |
| ARHGAP18-liver | LYRM7-muscle | -0.71818 | 0.0128 | 0.048396 |
| ARID5B-liver | LYRM7-muscle | -0.76364 | 0.006233 | 0.036383 |
| CD34-liver | LYRM7-muscle | -0.71818 | 0.0128 | 0.048396 |
| CLIC1-liver | LYRM7-muscle | -0.70909 | 0.014552 | 0.050131 |
| DSG2-liver | LYRM7-muscle | -0.74545 | 0.008455 | 0.041717 |
| FNBP1L-liver | LYRM7-muscle | -0.71818 | 0.0128 | 0.048396 |
| ITGB1-liver | LYRM7-muscle | -0.71818 | 0.0128 | 0.048396 |
| MFGE8-liver | LYRM7-muscle | -0.75455 | 0.007282 | 0.038865 |
| MMGT1-liver | LYRM7-muscle | -0.74545 | 0.008455 | 0.041717 |
| RAP2A-liver | LYRM7-muscle | -0.73636 | 0.00976 | 0.043199 |
| SLC25A6-liver | LYRM7-muscle | -0.71818 | 0.0128 | 0.048396 |
| STX7-liver | LYRM7-muscle | -0.74545 | 0.008455 | 0.041717 |
| SUCO-liver | LYRM7-muscle | -0.73636 | 0.00976 | 0.043199 |
| SULF1-liver | LYRM7-muscle | -0.70909 | 0.014552 | 0.050131 |
| YWHAZ-liver | LYRM7-muscle | -0.76364 | 0.006233 | 0.036383 |
| APP-liver | MICAL3-muscle | -0.7 | 0.016471 | 0.052234 |
| BMPR2-liver | MICAL3-muscle | -0.76364 | 0.006233 | 0.036383 |
| CA5B-liver | MICAL3-muscle | -0.76364 | 0.006233 | 0.036383 |
| DSG2-liver | MICAL3-muscle | -0.7 | 0.016471 | 0.052234 |
| IPO7-liver | MICAL3-muscle | -0.76364 | 0.006233 | 0.036383 |
| NCKAP1-liver | MICAL3-muscle | -0.70909 | 0.014552 | 0.050131 |
| RAP2A-liver | MICAL3-muscle | -0.7 | 0.016471 | 0.052234 |
| TTF2-liver | MICAL3-muscle | -0.81818 | 0.002083 | 0.027717 |
| APP-liver | MRPL15-muscle | -0.79091 | 0.003746 | 0.032221 |
| ARHGAP18-liver | MRPL15-muscle | -0.74545 | 0.008455 | 0.041717 |
| BMPR2-liver | MRPL15-muscle | -0.75455 | 0.007282 | 0.038865 |
| CDH5-liver | MRPL15-muscle | -0.71818 | 0.0128 | 0.048396 |
| DEFB1-liver | MRPL15-muscle | -0.7 | 0.016471 | 0.052234 |
| DSG2-liver | MRPL15-muscle | -0.79091 | 0.003746 | 0.032221 |
| EPS8-liver | MRPL15-muscle | -0.7 | 0.016471 | 0.052234 |
| FNBP1L-liver | MRPL15-muscle | -0.73636 | 0.00976 | 0.043199 |
| IPO7-liver | MRPL15-muscle | -0.7 | 0.016471 | 0.052234 |
| ITGB1-liver | MRPL15-muscle | -0.76364 | 0.006233 | 0.036383 |
| KHDRBS1-liver | MRPL15-muscle | -0.70909 | 0.014552 | 0.050131 |
| MFGE8-liver | MRPL15-muscle | -0.7 | 0.016471 | 0.052234 |
| MMGT1-liver | MRPL15-muscle | -0.8 | 0.00311 | 0.031511 |
| NCKAP1-liver | MRPL15-muscle | -0.77273 | 0.005299 | 0.035326 |
| PLPP5-liver | MRPL15-muscle | -0.74545 | 0.008455 | 0.041717 |
| RAP2A-liver | MRPL15-muscle | -0.70909 | 0.014552 | 0.050131 |
| SLC25A6-liver | MRPL15-muscle | -0.76364 | 0.006233 | 0.036383 |
| SUCO-liver | MRPL15-muscle | -0.75455 | 0.007282 | 0.038865 |
| TPM4-liver | MRPL15-muscle | -0.70909 | 0.014552 | 0.050131 |
| TTF2-liver | MRPL15-muscle | -0.7 | 0.016471 | 0.052234 |
| VCAN-liver | MRPL15-muscle | -0.70909 | 0.014552 | 0.050131 |
| YWHAZ-liver | MRPL15-muscle | -0.7 | 0.016471 | 0.052234 |
| APP-liver | MRPL27-muscle | -0.78182 | 0.004473 | 0.033769 |
| ARHGAP18-liver | MRPL27-muscle | -0.70909 | 0.014552 | 0.050131 |
| ARID5B-liver | MRPL27-muscle | -0.73636 | 0.00976 | 0.043199 |
| CCN4-liver | MRPL27-muscle | -0.71818 | 0.0128 | 0.048396 |
| CD34-liver | MRPL27-muscle | -0.75455 | 0.007282 | 0.038865 |
| CLIC1-liver | MRPL27-muscle | -0.78182 | 0.004473 | 0.033769 |
| DSG2-liver | MRPL27-muscle | -0.78182 | 0.004473 | 0.033769 |
| EPS8-liver | MRPL27-muscle | -0.79091 | 0.003746 | 0.032221 |
| EZR-liver | MRPL27-muscle | -0.78182 | 0.004473 | 0.033769 |
| FNBP1L-liver | MRPL27-muscle | -0.77273 | 0.005299 | 0.035326 |
| HEG1-liver | MRPL27-muscle | -0.78182 | 0.004473 | 0.033769 |
| ITGB1-liver | MRPL27-muscle | -0.77273 | 0.005299 | 0.035326 |
| KHDRBS1-liver | MRPL27-muscle | -0.7 | 0.016471 | 0.052234 |
| MFGE8-liver | MRPL27-muscle | -0.74545 | 0.008455 | 0.041717 |
| MMGT1-liver | MRPL27-muscle | -0.71818 | 0.0128 | 0.048396 |
| NCKAP1-liver | MRPL27-muscle | -0.74545 | 0.008455 | 0.041717 |
| PLPP5-liver | MRPL27-muscle | -0.85455 | 0.000807 | 0.022594 |
| PRKX-liver | MRPL27-muscle | -0.73636 | 0.00976 | 0.043199 |
| PRRC2C-liver | MRPL27-muscle | -0.70909 | 0.014552 | 0.050131 |
| RAP2A-liver | MRPL27-muscle | -0.74545 | 0.008455 | 0.041717 |
| SLC25A6-liver | MRPL27-muscle | -0.74545 | 0.008455 | 0.041717 |
| SMIM10-liver | MRPL27-muscle | -0.70909 | 0.014552 | 0.050131 |
| STX7-liver | MRPL27-muscle | -0.7 | 0.016471 | 0.052234 |
| SUCO-liver | MRPL27-muscle | -0.73636 | 0.00976 | 0.043199 |
| SULF1-liver | MRPL27-muscle | -0.78182 | 0.004473 | 0.033769 |
| TES-liver | MRPL27-muscle | -0.7 | 0.016471 | 0.052234 |
| TIMP1-liver | MRPL27-muscle | -0.71818 | 0.0128 | 0.048396 |
| TMSB10-liver | MRPL27-muscle | -0.73636 | 0.00976 | 0.043199 |
| VCAN-liver | MRPL27-muscle | -0.76364 | 0.006233 | 0.036383 |
| YWHAZ-liver | MRPL27-muscle | -0.80909 | 0.002559 | 0.029155 |
| APP-liver | MRPL44-muscle | -0.79091 | 0.003746 | 0.032221 |
| ARHGAP18-liver | MRPL44-muscle | -0.74545 | 0.008455 | 0.041717 |
| ARID5B-liver | MRPL44-muscle | -0.70909 | 0.014552 | 0.050131 |
| CA5B-liver | MRPL44-muscle | -0.70909 | 0.014552 | 0.050131 |
| CD34-liver | MRPL44-muscle | -0.71818 | 0.0128 | 0.048396 |
| CLIC1-liver | MRPL44-muscle | -0.70909 | 0.014552 | 0.050131 |
| DSG2-liver | MRPL44-muscle | -0.76364 | 0.006233 | 0.036383 |
| FNBP1L-liver | MRPL44-muscle | -0.74545 | 0.008455 | 0.041717 |
| ITGB1-liver | MRPL44-muscle | -0.71818 | 0.0128 | 0.048396 |
| KHDRBS1-liver | MRPL44-muscle | -0.7 | 0.016471 | 0.052234 |
| KIF3A-liver | MRPL44-muscle | -0.7 | 0.016471 | 0.052234 |
| MMGT1-liver | MRPL44-muscle | -0.76364 | 0.006233 | 0.036383 |
| NCKAP1-liver | MRPL44-muscle | -0.74545 | 0.008455 | 0.041717 |
| PRKX-liver | MRPL44-muscle | -0.7 | 0.016471 | 0.052234 |
| RAP2A-liver | MRPL44-muscle | -0.77273 | 0.005299 | 0.035326 |
| SLC25A6-liver | MRPL44-muscle | -0.73636 | 0.00976 | 0.043199 |
| SMIM10-liver | MRPL44-muscle | -0.71818 | 0.0128 | 0.048396 |
| STX7-liver | MRPL44-muscle | -0.77273 | 0.005299 | 0.035326 |
| SUCO-liver | MRPL44-muscle | -0.75455 | 0.007282 | 0.038865 |
| SULF1-liver | MRPL44-muscle | -0.70909 | 0.014552 | 0.050131 |
| TES-liver | MRPL44-muscle | -0.70909 | 0.014552 | 0.050131 |
| TTF2-liver | MRPL44-muscle | -0.76364 | 0.006233 | 0.036383 |
| YWHAZ-liver | MRPL44-muscle | -0.75455 | 0.007282 | 0.038865 |
| APP-liver | MRPL15-muscle | -0.73636 | 0.00976 | 0.043199 |
| ARID5B-liver | MRPL27-muscle | -0.71818 | 0.0128 | 0.048396 |
| DSG2-liver | MRPS18B-muscle | -0.71818 | 0.0128 | 0.048396 |
| ITGB1-liver | MRPS18B-muscle | -0.7 | 0.016471 | 0.052234 |
| MFGE8-liver | MRPS18B-muscle | -0.73636 | 0.00976 | 0.043199 |
| MMGT1-liver | MRPS18B-muscle | -0.7 | 0.016471 | 0.052234 |
| PLPP5-liver | MRPS18B-muscle | -0.7 | 0.016471 | 0.052234 |
| YWHAZ-liver | MRPS18B-muscle | -0.70909 | 0.014552 | 0.050131 |
| ANXA5-liver | MRPS30-muscle | -0.70909 | 0.014552 | 0.050131 |
| APP-liver | MRPS30-muscle | -0.79091 | 0.003746 | 0.032221 |
| ARHGAP18-liver | MRPS30-muscle | -0.76364 | 0.006233 | 0.036383 |
| ARID5B-liver | MRPS30-muscle | -0.8 | 0.00311 | 0.031511 |
| CCN4-liver | MRPS30-muscle | -0.7 | 0.016471 | 0.052234 |
| CD34-liver | MRPS30-muscle | -0.75455 | 0.007282 | 0.038865 |
| CDH5-liver | MRPS30-muscle | -0.71818 | 0.0128 | 0.048396 |
| CLIC1-liver | MRPS30-muscle | -0.74545 | 0.008455 | 0.041717 |
| DSG2-liver | MRPS30-muscle | -0.77273 | 0.005299 | 0.035326 |
| EPS8-liver | MRPS30-muscle | -0.70909 | 0.014552 | 0.050131 |
| FNBP1L-liver | MRPS30-muscle | -0.75455 | 0.007282 | 0.038865 |
| HEG1-liver | MRPS30-muscle | -0.72727 | 0.011205 | 0.045908 |
| ITGB1-liver | MRPS30-muscle | -0.73636 | 0.00976 | 0.043199 |
| KHDRBS1-liver | MRPS30-muscle | -0.7 | 0.016471 | 0.052234 |
| KIF3A-liver | MRPS30-muscle | -0.7 | 0.016471 | 0.052234 |
| MFGE8-liver | MRPS30-muscle | -0.78182 | 0.004473 | 0.033769 |
| MMGT1-liver | MRPS30-muscle | -0.78182 | 0.004473 | 0.033769 |
| NCKAP1-liver | MRPS30-muscle | -0.70909 | 0.014552 | 0.050131 |
| PLPP5-liver | MRPS30-muscle | -0.70909 | 0.014552 | 0.050131 |
| PRKX-liver | MRPS30-muscle | -0.7 | 0.016471 | 0.052234 |
| RAP2A-liver | MRPS30-muscle | -0.78182 | 0.004473 | 0.033769 |
| SLC25A6-liver | MRPS30-muscle | -0.74545 | 0.008455 | 0.041717 |
| SMIM10-liver | MRPS30-muscle | -0.73636 | 0.00976 | 0.043199 |
| STX7-liver | MRPS30-muscle | -0.78182 | 0.004473 | 0.033769 |
| SUCO-liver | MRPS30-muscle | -0.77273 | 0.005299 | 0.035326 |
| SULF1-liver | MRPS30-muscle | -0.74545 | 0.008455 | 0.041717 |
| TES-liver | MRPS30-muscle | -0.70909 | 0.014552 | 0.050131 |
| TIMP1-liver | MRPS30-muscle | -0.72727 | 0.011205 | 0.045908 |
| TMSB10-liver | MRPS30-muscle | -0.70909 | 0.014552 | 0.050131 |
| VCAN-liver | MRPS30-muscle | -0.73636 | 0.00976 | 0.043199 |
| YWHAZ-liver | MRPS30-muscle | -0.79091 | 0.003746 | 0.032221 |
| ANXA5-liver | MRS2-muscle | -0.71818 | 0.0128 | 0.048396 |
| APP-liver | MRS2-muscle | -0.8 | 0.00311 | 0.031511 |
| ARID5B-liver | MRS2-muscle | -0.73636 | 0.00976 | 0.043199 |
| CD34-liver | MRS2-muscle | -0.8 | 0.00311 | 0.031511 |
| CLIC1-liver | MRS2-muscle | -0.79091 | 0.003746 | 0.032221 |
| DSG2-liver | MRS2-muscle | -0.72727 | 0.011205 | 0.045908 |
| FNBP1L-liver | MRS2-muscle | -0.71818 | 0.0128 | 0.048396 |
| HEG1-liver | MRS2-muscle | -0.71818 | 0.0128 | 0.048396 |
| KHDRBS1-liver | MRS2-muscle | -0.70909 | 0.014552 | 0.050131 |
| MFGE8-liver | MRS2-muscle | -0.70909 | 0.014552 | 0.050131 |
| NCKAP1-liver | MRS2-muscle | -0.7 | 0.016471 | 0.052234 |
| PRKX-liver | MRS2-muscle | -0.73636 | 0.00976 | 0.043199 |
| RAP2A-liver | MRS2-muscle | -0.78182 | 0.004473 | 0.033769 |
| SLC25A6-liver | MRS2-muscle | -0.7 | 0.016471 | 0.052234 |
| SMIM10-liver | MRS2-muscle | -0.72727 | 0.011205 | 0.045908 |
| STX7-liver | MRS2-muscle | -0.77273 | 0.005299 | 0.035326 |
| SUCO-liver | MRS2-muscle | -0.7 | 0.016471 | 0.052234 |
| SULF1-liver | MRS2-muscle | -0.79091 | 0.003746 | 0.032221 |
| TES-liver | MRS2-muscle | -0.7 | 0.016471 | 0.052234 |
| TIMP1-liver | MRS2-muscle | -0.77273 | 0.005299 | 0.035326 |
| TMSB10-liver | MRS2-muscle | -0.72727 | 0.011205 | 0.045908 |
| YWHAZ-liver | MRS2-muscle | -0.84545 | 0.001045 | 0.024446 |
| ADGRL4-liver | MSI2-muscle | -0.73636 | 0.00976 | 0.043199 |
| ANXA5-liver | MSI2-muscle | -0.74545 | 0.008455 | 0.041717 |
| APP-liver | MSI2-muscle | -0.85455 | 0.000807 | 0.022594 |
| ARHGAP18-liver | MSI2-muscle | -0.78182 | 0.004473 | 0.033769 |
| ARID5B-liver | MSI2-muscle | -0.70909 | 0.014552 | 0.050131 |
| BMPR2-liver | MSI2-muscle | -0.77273 | 0.005299 | 0.035326 |
| CCN4-liver | MSI2-muscle | -0.73636 | 0.00976 | 0.043199 |
| CD34-liver | MSI2-muscle | -0.71818 | 0.0128 | 0.048396 |
| CDH5-liver | MSI2-muscle | -0.74545 | 0.008455 | 0.041717 |
| CLIC1-liver | MSI2-muscle | -0.76364 | 0.006233 | 0.036383 |
| DEFB1-liver | MSI2-muscle | -0.79091 | 0.003746 | 0.032221 |
| DSG2-liver | MSI2-muscle | -0.82727 | 0.001677 | 0.02618 |
| EPS8-liver | MSI2-muscle | -0.72727 | 0.011205 | 0.045908 |
| EZR-liver | MSI2-muscle | -0.70909 | 0.014552 | 0.050131 |
| FNBP1L-liver | MSI2-muscle | -0.77273 | 0.005299 | 0.035326 |
| HEG1-liver | MSI2-muscle | -0.8 | 0.00311 | 0.031511 |
| IFT57-liver | MSI2-muscle | -0.80909 | 0.002559 | 0.029155 |
| IPO7-liver | MSI2-muscle | -0.7 | 0.016471 | 0.052234 |
| ITGB1-liver | MSI2-muscle | -0.8 | 0.00311 | 0.031511 |
| KHDRBS1-liver | MSI2-muscle | -0.8 | 0.00311 | 0.031511 |
| KIF3A-liver | MSI2-muscle | -0.76364 | 0.006233 | 0.036383 |
| MFGE8-liver | MSI2-muscle | -0.75455 | 0.007282 | 0.038865 |
| MMGT1-liver | MSI2-muscle | -0.83636 | 0.001333 | 0.025685 |
| MYH9-liver | MSI2-muscle | -0.80909 | 0.002559 | 0.029155 |
| NCKAP1-liver | MSI2-muscle | -0.80909 | 0.002559 | 0.029155 |
| PLPP5-liver | MSI2-muscle | -0.75455 | 0.007282 | 0.038865 |
| PRKX-liver | MSI2-muscle | -0.75455 | 0.007282 | 0.038865 |
| RAP2A-liver | MSI2-muscle | -0.76364 | 0.006233 | 0.036383 |
| SLC25A6-liver | MSI2-muscle | -0.80909 | 0.002559 | 0.029155 |
| SMIM10-liver | MSI2-muscle | -0.72727 | 0.011205 | 0.045908 |
| STX7-liver | MSI2-muscle | -0.71818 | 0.0128 | 0.048396 |
| SUCO-liver | MSI2-muscle | -0.79091 | 0.003746 | 0.032221 |
| SULF1-liver | MSI2-muscle | -0.76364 | 0.006233 | 0.036383 |
| TES-liver | MSI2-muscle | -0.77273 | 0.005299 | 0.035326 |
| TIMP1-liver | MSI2-muscle | -0.7 | 0.016471 | 0.052234 |
| TMSB10-liver | MSI2-muscle | -0.79091 | 0.003746 | 0.032221 |
| TPM4-liver | MSI2-muscle | -0.79091 | 0.003746 | 0.032221 |
| TSPAN14-liver | MSI2-muscle | -0.77273 | 0.005299 | 0.035326 |
| VCAN-liver | MSI2-muscle | -0.78182 | 0.004473 | 0.033769 |
| YWHAZ-liver | MSI2-muscle | -0.78182 | 0.004473 | 0.033769 |
| ANXA5-liver | MTRES1-muscle | -0.77273 | 0.005299 | 0.035326 |
| APP-liver | MTRES1-muscle | -0.78182 | 0.004473 | 0.033769 |
| ARHGAP18-liver | MTRES1-muscle | -0.7 | 0.016471 | 0.052234 |
| ARID5B-liver | MTRES1-muscle | -0.78182 | 0.004473 | 0.033769 |
| CD34-liver | MTRES1-muscle | -0.8 | 0.00311 | 0.031511 |
| CLIC1-liver | MTRES1-muscle | -0.79091 | 0.003746 | 0.032221 |
| DSG2-liver | MTRES1-muscle | -0.70909 | 0.014552 | 0.050131 |
| HEG1-liver | MTRES1-muscle | -0.77273 | 0.005299 | 0.035326 |
| IFT57-liver | MTRES1-muscle | -0.74545 | 0.008455 | 0.041717 |
| KHDRBS1-liver | MTRES1-muscle | -0.70909 | 0.014552 | 0.050131 |
| KIF3A-liver | MTRES1-muscle | -0.70909 | 0.014552 | 0.050131 |
| MFGE8-liver | MTRES1-muscle | -0.77273 | 0.005299 | 0.035326 |
| MMGT1-liver | MTRES1-muscle | -0.71818 | 0.0128 | 0.048396 |
| MYH9-liver | MTRES1-muscle | -0.72727 | 0.011205 | 0.045908 |
| PRKX-liver | MTRES1-muscle | -0.7 | 0.016471 | 0.052234 |
| RAP2A-liver | MTRES1-muscle | -0.76364 | 0.006233 | 0.036383 |
| SMIM10-liver | MTRES1-muscle | -0.70909 | 0.014552 | 0.050131 |
| STX7-liver | MTRES1-muscle | -0.77273 | 0.005299 | 0.035326 |
| SUCO-liver | MTRES1-muscle | -0.70909 | 0.014552 | 0.050131 |
| SULF1-liver | MTRES1-muscle | -0.79091 | 0.003746 | 0.032221 |
| TES-liver | MTRES1-muscle | -0.71818 | 0.0128 | 0.048396 |
| TIMP1-liver | MTRES1-muscle | -0.78182 | 0.004473 | 0.033769 |
| TMSB10-liver | MTRES1-muscle | -0.76364 | 0.006233 | 0.036383 |
| VCAN-liver | MTRES1-muscle | -0.72727 | 0.011205 | 0.045908 |
| YWHAZ-liver | MTRES1-muscle | -0.82727 | 0.001677 | 0.02618 |
| APP-liver | MTX2-muscle | -0.77273 | 0.005299 | 0.035326 |
| ARHGAP18-liver | MTX2-muscle | -0.70909 | 0.014552 | 0.050131 |
| ARID5B-liver | MTX2-muscle | -0.75455 | 0.007282 | 0.038865 |
| CD34-liver | MTX2-muscle | -0.72727 | 0.011205 | 0.045908 |
| CLIC1-liver | MTX2-muscle | -0.73636 | 0.00976 | 0.043199 |
| DSG2-liver | MTX2-muscle | -0.72727 | 0.011205 | 0.045908 |
| FNBP1L-liver | MTX2-muscle | -0.7 | 0.016471 | 0.052234 |
| HEG1-liver | MTX2-muscle | -0.74545 | 0.008455 | 0.041717 |
| MFGE8-liver | MTX2-muscle | -0.71818 | 0.0128 | 0.048396 |
| MMGT1-liver | MTX2-muscle | -0.73636 | 0.00976 | 0.043199 |
| RAP2A-liver | MTX2-muscle | -0.76364 | 0.006233 | 0.036383 |
| SMIM10-liver | MTX2-muscle | -0.73636 | 0.00976 | 0.043199 |
| STX7-liver | MTX2-muscle | -0.70909 | 0.014552 | 0.050131 |
| SUCO-liver | MTX2-muscle | -0.7 | 0.016471 | 0.052234 |
| SULF1-liver | MTX2-muscle | -0.73636 | 0.00976 | 0.043199 |
| TMSB10-liver | MTX2-muscle | -0.71818 | 0.0128 | 0.048396 |
| TPM4-liver | MTX2-muscle | -0.7 | 0.016471 | 0.052234 |
| VCAN-liver | MTX2-muscle | -0.71818 | 0.0128 | 0.048396 |
| YWHAZ-liver | MTX2-muscle | -0.79091 | 0.003746 | 0.032221 |
| ANXA5-liver | MYLK2-muscle | -0.70909 | 0.014552 | 0.050131 |
| APP-liver | MYLK2-muscle | -0.77273 | 0.005299 | 0.035326 |
| ARID5B-liver | MYLK2-muscle | -0.72727 | 0.011205 | 0.045908 |
| CD34-liver | MYLK2-muscle | -0.75455 | 0.007282 | 0.038865 |
| CLIC1-liver | MYLK2-muscle | -0.76364 | 0.006233 | 0.036383 |
| DSG2-liver | MYLK2-muscle | -0.7 | 0.016471 | 0.052234 |
| HEG1-liver | MYLK2-muscle | -0.75455 | 0.007282 | 0.038865 |
| IFT57-liver | MYLK2-muscle | -0.7 | 0.016471 | 0.052234 |
| MFGE8-liver | MYLK2-muscle | -0.71818 | 0.0128 | 0.048396 |
| RAP2A-liver | MYLK2-muscle | -0.73636 | 0.00976 | 0.043199 |
| STX7-liver | MYLK2-muscle | -0.70909 | 0.014552 | 0.050131 |
| SULF1-liver | MYLK2-muscle | -0.76364 | 0.006233 | 0.036383 |
| TIMP1-liver | MYLK2-muscle | -0.71818 | 0.0128 | 0.048396 |
| TMSB10-liver | MYLK2-muscle | -0.73636 | 0.00976 | 0.043199 |
| VCAN-liver | MYLK2-muscle | -0.7 | 0.016471 | 0.052234 |
| YWHAZ-liver | MYLK2-muscle | -0.80909 | 0.002559 | 0.029155 |
| ALCAM-liver | MYOM2-muscle | -0.75455 | 0.007282 | 0.038865 |
| APP-liver | MYOM2-muscle | -0.71818 | 0.0128 | 0.048396 |
| ARHGAP18-liver | MYOM2-muscle | -0.70909 | 0.014552 | 0.050131 |
| CDH5-liver | MYOM2-muscle | -0.7 | 0.016471 | 0.052234 |
| DEFB1-liver | MYOM2-muscle | -0.73636 | 0.00976 | 0.043199 |
| DSG2-liver | MYOM2-muscle | -0.71818 | 0.0128 | 0.048396 |
| MMGT1-liver | MYOM2-muscle | -0.76364 | 0.006233 | 0.036383 |
| NCKAP1-liver | MYOM2-muscle | -0.72727 | 0.011205 | 0.045908 |
| SLC25A6-liver | MYOM2-muscle | -0.72727 | 0.011205 | 0.045908 |
| SUCO-liver | MYOM2-muscle | -0.71818 | 0.0128 | 0.048396 |
| TTF2-liver | MYOM2-muscle | -0.76364 | 0.006233 | 0.036383 |
| ALCAM-liver | NBR1-muscle | -0.70909 | 0.014552 | 0.050131 |
| ARHGAP18-liver | NBR1-muscle | -0.70909 | 0.014552 | 0.050131 |
| DSG2-liver | NBR1-muscle | -0.70909 | 0.014552 | 0.050131 |
| MMGT1-liver | NBR1-muscle | -0.72727 | 0.011205 | 0.045908 |
| NCKAP1-liver | NBR1-muscle | -0.7 | 0.016471 | 0.052234 |
| PLPP5-liver | NBR1-muscle | -0.7 | 0.016471 | 0.052234 |
| PRRC2C-liver | NBR1-muscle | -0.71818 | 0.0128 | 0.048396 |
| SLC25A6-liver | NBR1-muscle | -0.70909 | 0.014552 | 0.050131 |
| SUCO-liver | NBR1-muscle | -0.71818 | 0.0128 | 0.048396 |
| TTF2-liver | NBR1-muscle | -0.87273 | 0.000455 | 0.020203 |
| ADGRL4-liver | NDUFS1-muscle | -0.84545 | 0.001045 | 0.024446 |
| ALCAM-liver | NDUFS1-muscle | -0.74545 | 0.008455 | 0.041717 |
| ANXA5-liver | NDUFS1-muscle | -0.76364 | 0.006233 | 0.036383 |
| APP-liver | NDUFS1-muscle | -0.88182 | 0.00033 | 0.018341 |
| ARHGAP18-liver | NDUFS1-muscle | -0.82727 | 0.001677 | 0.02618 |
| ARID5B-liver | NDUFS1-muscle | -0.73636 | 0.00976 | 0.043199 |
| B4GALT5-liver | NDUFS1-muscle | -0.77273 | 0.005299 | 0.035326 |
| BMPR2-liver | NDUFS1-muscle | -0.79091 | 0.003746 | 0.032221 |
| CCN4-liver | NDUFS1-muscle | -0.81818 | 0.002083 | 0.027717 |
| CD34-liver | NDUFS1-muscle | -0.71818 | 0.0128 | 0.048396 |
| CDH5-liver | NDUFS1-muscle | -0.8 | 0.00311 | 0.031511 |
| CLIC1-liver | NDUFS1-muscle | -0.74545 | 0.008455 | 0.041717 |
| DEFB1-liver | NDUFS1-muscle | -0.85455 | 0.000807 | 0.022594 |
| DSG2-liver | NDUFS1-muscle | -0.88182 | 0.00033 | 0.018341 |
| EPS8-liver | NDUFS1-muscle | -0.77273 | 0.005299 | 0.035326 |
| EZR-liver | NDUFS1-muscle | -0.71818 | 0.0128 | 0.048396 |
| FNBP1L-liver | NDUFS1-muscle | -0.82727 | 0.001677 | 0.02618 |
| HDAC2-liver | NDUFS1-muscle | -0.77273 | 0.005299 | 0.035326 |
| HEG1-liver | NDUFS1-muscle | -0.74545 | 0.008455 | 0.041717 |
| IFT57-liver | NDUFS1-muscle | -0.8 | 0.00311 | 0.031511 |
| IPO7-liver | NDUFS1-muscle | -0.8 | 0.00311 | 0.031511 |
| ITGB1-liver | NDUFS1-muscle | -0.86364 | 0.000612 | 0.022041 |
| KHDRBS1-liver | NDUFS1-muscle | -0.82727 | 0.001677 | 0.02618 |
| KIF3A-liver | NDUFS1-muscle | -0.79091 | 0.003746 | 0.032221 |
| KIF5B-liver | NDUFS1-muscle | -0.7 | 0.016471 | 0.052234 |
| MFGE8-liver | NDUFS1-muscle | -0.8 | 0.00311 | 0.031511 |
| MFSD6-liver | NDUFS1-muscle | -0.7 | 0.016471 | 0.052234 |
| MKKS-liver | NDUFS1-muscle | -0.7 | 0.016471 | 0.052234 |
| MMGT1-liver | NDUFS1-muscle | -0.88182 | 0.00033 | 0.018341 |
| MTMR6-liver | NDUFS1-muscle | -0.7 | 0.016471 | 0.052234 |
| MYH9-liver | NDUFS1-muscle | -0.83636 | 0.001333 | 0.025685 |
| NCKAP1-liver | NDUFS1-muscle | -0.85455 | 0.000807 | 0.022594 |
| PLPP5-liver | NDUFS1-muscle | -0.77273 | 0.005299 | 0.035326 |
| PRKX-liver | NDUFS1-muscle | -0.76364 | 0.006233 | 0.036383 |
| PRRC2C-liver | NDUFS1-muscle | -0.74545 | 0.008455 | 0.041717 |
| RALB-liver | NDUFS1-muscle | -0.77273 | 0.005299 | 0.035326 |
| RAP2A-liver | NDUFS1-muscle | -0.76364 | 0.006233 | 0.036383 |
| RPS6KA2-liver | NDUFS1-muscle | -0.78182 | 0.004473 | 0.033769 |
| SLC25A6-liver | NDUFS1-muscle | -0.88182 | 0.00033 | 0.018341 |
| SMIM10-liver | NDUFS1-muscle | -0.71818 | 0.0128 | 0.048396 |
| STX7-liver | NDUFS1-muscle | -0.77273 | 0.005299 | 0.035326 |
| SUCO-liver | NDUFS1-muscle | -0.85455 | 0.000807 | 0.022594 |
| SULF1-liver | NDUFS1-muscle | -0.74545 | 0.008455 | 0.041717 |
| TES-liver | NDUFS1-muscle | -0.81818 | 0.002083 | 0.027717 |
| TIMP1-liver | NDUFS1-muscle | -0.71818 | 0.0128 | 0.048396 |
| TMSB10-liver | NDUFS1-muscle | -0.75455 | 0.007282 | 0.038865 |
| TPM4-liver | NDUFS1-muscle | -0.79091 | 0.003746 | 0.032221 |
| TSPAN14-liver | NDUFS1-muscle | -0.74545 | 0.008455 | 0.041717 |
| TTF2-liver | NDUFS1-muscle | -0.79091 | 0.003746 | 0.032221 |
| VCAN-liver | NDUFS1-muscle | -0.77273 | 0.005299 | 0.035326 |
| YWHAZ-liver | NDUFS1-muscle | -0.76364 | 0.006233 | 0.036383 |
| ADGRL4-liver | NDUFS7-muscle | -0.70909 | 0.014552 | 0.050131 |
| ANKRD27-liver | NDUFS7-muscle | -0.74545 | 0.008455 | 0.041717 |
| APP-liver | NDUFS7-muscle | -0.81818 | 0.002083 | 0.027717 |
| ARHGAP18-liver | NDUFS7-muscle | -0.77273 | 0.005299 | 0.035326 |
| B4GALT5-liver | NDUFS7-muscle | -0.70909 | 0.014552 | 0.050131 |
| BMPR2-liver | NDUFS7-muscle | -0.83636 | 0.001333 | 0.025685 |
| CCN4-liver | NDUFS7-muscle | -0.72727 | 0.011205 | 0.045908 |
| CDH5-liver | NDUFS7-muscle | -0.76364 | 0.006233 | 0.036383 |
| CLIC1-liver | NDUFS7-muscle | -0.73636 | 0.00976 | 0.043199 |
| DEFB1-liver | NDUFS7-muscle | -0.71818 | 0.0128 | 0.048396 |
| DSG2-liver | NDUFS7-muscle | -0.81818 | 0.002083 | 0.027717 |
| EPS8-liver | NDUFS7-muscle | -0.73636 | 0.00976 | 0.043199 |
| EZR-liver | NDUFS7-muscle | -0.73636 | 0.00976 | 0.043199 |
| FNBP1L-liver | NDUFS7-muscle | -0.77273 | 0.005299 | 0.035326 |
| HEG1-liver | NDUFS7-muscle | -0.78182 | 0.004473 | 0.033769 |
| IFT57-liver | NDUFS7-muscle | -0.73636 | 0.00976 | 0.043199 |
| IPO7-liver | NDUFS7-muscle | -0.77273 | 0.005299 | 0.035326 |
| ITGB1-liver | NDUFS7-muscle | -0.80909 | 0.002559 | 0.029155 |
| KHDRBS1-liver | NDUFS7-muscle | -0.75455 | 0.007282 | 0.038865 |
| KIF3A-liver | NDUFS7-muscle | -0.72727 | 0.011205 | 0.045908 |
| KIF5B-liver | NDUFS7-muscle | -0.73636 | 0.00976 | 0.043199 |
| MMGT1-liver | NDUFS7-muscle | -0.81818 | 0.002083 | 0.027717 |
| MYH9-liver | NDUFS7-muscle | -0.75455 | 0.007282 | 0.038865 |
| NCKAP1-liver | NDUFS7-muscle | -0.8 | 0.00311 | 0.031511 |
| PLPP5-liver | NDUFS7-muscle | -0.79091 | 0.003746 | 0.032221 |
| PRKX-liver | NDUFS7-muscle | -0.73636 | 0.00976 | 0.043199 |
| RAP2A-liver | NDUFS7-muscle | -0.75455 | 0.007282 | 0.038865 |
| SLC25A6-liver | NDUFS7-muscle | -0.78182 | 0.004473 | 0.033769 |
| SMIM10-liver | NDUFS7-muscle | -0.73636 | 0.00976 | 0.043199 |
| SUCO-liver | NDUFS7-muscle | -0.78182 | 0.004473 | 0.033769 |
| SULF1-liver | NDUFS7-muscle | -0.73636 | 0.00976 | 0.043199 |
| TES-liver | NDUFS7-muscle | -0.73636 | 0.00976 | 0.043199 |
| TMSB10-liver | NDUFS7-muscle | -0.76364 | 0.006233 | 0.036383 |
| TPM4-liver | NDUFS7-muscle | -0.78182 | 0.004473 | 0.033769 |
| TSPAN14-liver | NDUFS7-muscle | -0.71818 | 0.0128 | 0.048396 |
| VCAN-liver | NDUFS7-muscle | -0.76364 | 0.006233 | 0.036383 |
| YWHAZ-liver | NDUFS7-muscle | -0.74545 | 0.008455 | 0.041717 |
| ANXA5-liver | NFS1-muscle | -0.71818 | 0.0128 | 0.048396 |
| APP-liver | NFS1-muscle | -0.82727 | 0.001677 | 0.02618 |
| ARHGAP18-liver | NFS1-muscle | -0.74545 | 0.008455 | 0.041717 |
| ARID5B-liver | NFS1-muscle | -0.79091 | 0.003746 | 0.032221 |
| CCN4-liver | NFS1-muscle | -0.71818 | 0.0128 | 0.048396 |
| CD34-liver | NFS1-muscle | -0.78182 | 0.004473 | 0.033769 |
| CLIC1-liver | NFS1-muscle | -0.77273 | 0.005299 | 0.035326 |
| DSG2-liver | NFS1-muscle | -0.8 | 0.00311 | 0.031511 |
| EPS8-liver | NFS1-muscle | -0.71818 | 0.0128 | 0.048396 |
| FNBP1L-liver | NFS1-muscle | -0.78182 | 0.004473 | 0.033769 |
| HEG1-liver | NFS1-muscle | -0.71818 | 0.0128 | 0.048396 |
| ITGB1-liver | NFS1-muscle | -0.78182 | 0.004473 | 0.033769 |
| KHDRBS1-liver | NFS1-muscle | -0.7 | 0.016471 | 0.052234 |
| KIF3A-liver | NFS1-muscle | -0.7 | 0.016471 | 0.052234 |
| MFGE8-liver | NFS1-muscle | -0.78182 | 0.004473 | 0.033769 |
| MMGT1-liver | NFS1-muscle | -0.76364 | 0.006233 | 0.036383 |
| NCKAP1-liver | NFS1-muscle | -0.72727 | 0.011205 | 0.045908 |
| PLPP5-liver | NFS1-muscle | -0.72727 | 0.011205 | 0.045908 |
| PRKX-liver | NFS1-muscle | -0.70909 | 0.014552 | 0.050131 |
| RAP2A-liver | NFS1-muscle | -0.78182 | 0.004473 | 0.033769 |
| RPS6KA2-liver | NFS1-muscle | -0.7 | 0.016471 | 0.052234 |
| SLC25A6-liver | NFS1-muscle | -0.76364 | 0.006233 | 0.036383 |
| SMIM10-liver | NFS1-muscle | -0.73636 | 0.00976 | 0.043199 |
| STX7-liver | NFS1-muscle | -0.79091 | 0.003746 | 0.032221 |
| SUCO-liver | NFS1-muscle | -0.77273 | 0.005299 | 0.035326 |
| SULF1-liver | NFS1-muscle | -0.77273 | 0.005299 | 0.035326 |
| TES-liver | NFS1-muscle | -0.72727 | 0.011205 | 0.045908 |
| TIMP1-liver | NFS1-muscle | -0.74545 | 0.008455 | 0.041717 |
| TMSB10-liver | NFS1-muscle | -0.70909 | 0.014552 | 0.050131 |
| TPM4-liver | NFS1-muscle | -0.7 | 0.016471 | 0.052234 |
| VCAN-liver | NFS1-muscle | -0.71818 | 0.0128 | 0.048396 |
| YWHAZ-liver | NFS1-muscle | -0.82727 | 0.001677 | 0.02618 |
| ADGRL4-liver | NNT-muscle | -0.8 | 0.00311 | 0.031511 |
| ALCAM-liver | NNT-muscle | -0.8 | 0.00311 | 0.031511 |
| ANXA5-liver | NNT-muscle | -0.73636 | 0.00976 | 0.043199 |
| APP-liver | NNT-muscle | -0.80909 | 0.002559 | 0.029155 |
| ARHGAP18-liver | NNT-muscle | -0.80909 | 0.002559 | 0.029155 |
| ARID5B-liver | NNT-muscle | -0.73636 | 0.00976 | 0.043199 |
| CCN4-liver | NNT-muscle | -0.78182 | 0.004473 | 0.033769 |
| CDH5-liver | NNT-muscle | -0.77273 | 0.005299 | 0.035326 |
| DEFB1-liver | NNT-muscle | -0.83636 | 0.001333 | 0.025685 |
| DSG2-liver | NNT-muscle | -0.82727 | 0.001677 | 0.02618 |
| EPS8-liver | NNT-muscle | -0.76364 | 0.006233 | 0.036383 |
| FNBP1L-liver | NNT-muscle | -0.78182 | 0.004473 | 0.033769 |
| HDAC2-liver | NNT-muscle | -0.71818 | 0.0128 | 0.048396 |
| IFT57-liver | NNT-muscle | -0.76364 | 0.006233 | 0.036383 |
| IPO7-liver | NNT-muscle | -0.72727 | 0.011205 | 0.045908 |
| ITGB1-liver | NNT-muscle | -0.78182 | 0.004473 | 0.033769 |
| KHDRBS1-liver | NNT-muscle | -0.79091 | 0.003746 | 0.032221 |
| KIF3A-liver | NNT-muscle | -0.76364 | 0.006233 | 0.036383 |
| MFGE8-liver | NNT-muscle | -0.79091 | 0.003746 | 0.032221 |
| MFSD6-liver | NNT-muscle | -0.72727 | 0.011205 | 0.045908 |
| MMGT1-liver | NNT-muscle | -0.85455 | 0.000807 | 0.022594 |
| MYH9-liver | NNT-muscle | -0.77273 | 0.005299 | 0.035326 |
| NCKAP1-liver | NNT-muscle | -0.81818 | 0.002083 | 0.027717 |
| PLPP5-liver | NNT-muscle | -0.75455 | 0.007282 | 0.038865 |
| PRKX-liver | NNT-muscle | -0.71818 | 0.0128 | 0.048396 |
| PRRC2C-liver | NNT-muscle | -0.75455 | 0.007282 | 0.038865 |
| RALB-liver | NNT-muscle | -0.74545 | 0.008455 | 0.041717 |
| RAP2A-liver | NNT-muscle | -0.71818 | 0.0128 | 0.048396 |
| RPS6KA2-liver | NNT-muscle | -0.70909 | 0.014552 | 0.050131 |
| SLC25A6-liver | NNT-muscle | -0.84545 | 0.001045 | 0.024446 |
| STX7-liver | NNT-muscle | -0.75455 | 0.007282 | 0.038865 |
| SUCO-liver | NNT-muscle | -0.82727 | 0.001677 | 0.02618 |
| TES-liver | NNT-muscle | -0.78182 | 0.004473 | 0.033769 |
| TMSB10-liver | NNT-muscle | -0.7 | 0.016471 | 0.052234 |
| TPM4-liver | NNT-muscle | -0.71818 | 0.0128 | 0.048396 |
| TSPAN14-liver | NNT-muscle | -0.70909 | 0.014552 | 0.050131 |
| TTF2-liver | NNT-muscle | -0.82727 | 0.001677 | 0.02618 |
| VCAN-liver | NNT-muscle | -0.74545 | 0.008455 | 0.041717 |
| ANXA5-liver | NOSIP-muscle | -0.71818 | 0.0128 | 0.048396 |
| APP-liver | NOSIP-muscle | -0.82727 | 0.001677 | 0.02618 |
| ARID5B-liver | NOSIP-muscle | -0.76364 | 0.006233 | 0.036383 |
| CCN4-liver | NOSIP-muscle | -0.7 | 0.016471 | 0.052234 |
| CD34-liver | NOSIP-muscle | -0.78182 | 0.004473 | 0.033769 |
| CLIC1-liver | NOSIP-muscle | -0.79091 | 0.003746 | 0.032221 |
| DSG2-liver | NOSIP-muscle | -0.78182 | 0.004473 | 0.033769 |
| EPS8-liver | NOSIP-muscle | -0.71818 | 0.0128 | 0.048396 |
| EZR-liver | NOSIP-muscle | -0.72727 | 0.011205 | 0.045908 |
| FNBP1L-liver | NOSIP-muscle | -0.75455 | 0.007282 | 0.038865 |
| HEG1-liver | NOSIP-muscle | -0.75455 | 0.007282 | 0.038865 |
| ITGB1-liver | NOSIP-muscle | -0.77273 | 0.005299 | 0.035326 |
| KHDRBS1-liver | NOSIP-muscle | -0.71818 | 0.0128 | 0.048396 |
| MFGE8-liver | NOSIP-muscle | -0.77273 | 0.005299 | 0.035326 |
| MMGT1-liver | NOSIP-muscle | -0.71818 | 0.0128 | 0.048396 |
| MYH9-liver | NOSIP-muscle | -0.70909 | 0.014552 | 0.050131 |
| NCKAP1-liver | NOSIP-muscle | -0.72727 | 0.011205 | 0.045908 |
| PLPP5-liver | NOSIP-muscle | -0.74545 | 0.008455 | 0.041717 |
| PRKX-liver | NOSIP-muscle | -0.72727 | 0.011205 | 0.045908 |
| RAP2A-liver | NOSIP-muscle | -0.74545 | 0.008455 | 0.041717 |
| SLC25A6-liver | NOSIP-muscle | -0.75455 | 0.007282 | 0.038865 |
| SMIM10-liver | NOSIP-muscle | -0.7 | 0.016471 | 0.052234 |
| STX7-liver | NOSIP-muscle | -0.73636 | 0.00976 | 0.043199 |
| SUCO-liver | NOSIP-muscle | -0.72727 | 0.011205 | 0.045908 |
| SULF1-liver | NOSIP-muscle | -0.79091 | 0.003746 | 0.032221 |
| TES-liver | NOSIP-muscle | -0.70909 | 0.014552 | 0.050131 |
| TIMP1-liver | NOSIP-muscle | -0.75455 | 0.007282 | 0.038865 |
| TMSB10-liver | NOSIP-muscle | -0.72727 | 0.011205 | 0.045908 |
| VCAN-liver | NOSIP-muscle | -0.72727 | 0.011205 | 0.045908 |
| YWHAZ-liver | NOSIP-muscle | -0.84545 | 0.001045 | 0.024446 |
| ABI2-liver | NUDT12-muscle | -0.72727 | 0.011205 | 0.045908 |
| ADGRL4-liver | NUDT12-muscle | -0.76364 | 0.006233 | 0.036383 |
| ANXA5-liver | NUDT12-muscle | -0.85455 | 0.000807 | 0.022594 |
| APP-liver | NUDT12-muscle | -0.84545 | 0.001045 | 0.024446 |
| ARHGAP18-liver | NUDT12-muscle | -0.85455 | 0.000807 | 0.022594 |
| ARID5B-liver | NUDT12-muscle | -0.90909 | 0.000106 | 0.016759 |
| CAMSAP2-liver | NUDT12-muscle | -0.74545 | 0.008455 | 0.041717 |
| CCN4-liver | NUDT12-muscle | -0.81818 | 0.002083 | 0.027717 |
| CD34-liver | NUDT12-muscle | -0.85455 | 0.000807 | 0.022594 |
| CDH5-liver | NUDT12-muscle | -0.81818 | 0.002083 | 0.027717 |
| CLIC1-liver | NUDT12-muscle | -0.83636 | 0.001333 | 0.025685 |
| DEFB1-liver | NUDT12-muscle | -0.76364 | 0.006233 | 0.036383 |
| DSG2-liver | NUDT12-muscle | -0.83636 | 0.001333 | 0.025685 |
| EPS8-liver | NUDT12-muscle | -0.80909 | 0.002559 | 0.029155 |
| EZR-liver | NUDT12-muscle | -0.75455 | 0.007282 | 0.038865 |
| FNBP1L-liver | NUDT12-muscle | -0.82727 | 0.001677 | 0.02618 |
| HDAC2-liver | NUDT12-muscle | -0.7 | 0.016471 | 0.052234 |
| HEG1-liver | NUDT12-muscle | -0.83636 | 0.001333 | 0.025685 |
| IFT57-liver | NUDT12-muscle | -0.83636 | 0.001333 | 0.025685 |
| ITGB1-liver | NUDT12-muscle | -0.79091 | 0.003746 | 0.032221 |
| KHDRBS1-liver | NUDT12-muscle | -0.80909 | 0.002559 | 0.029155 |
| KIF3A-liver | NUDT12-muscle | -0.81818 | 0.002083 | 0.027717 |
| MFGE8-liver | NUDT12-muscle | -0.90909 | 0.000106 | 0.016759 |
| MFSD6-liver | NUDT12-muscle | -0.76364 | 0.006233 | 0.036383 |
| MMGT1-liver | NUDT12-muscle | -0.86364 | 0.000612 | 0.022041 |
| MYH9-liver | NUDT12-muscle | -0.80909 | 0.002559 | 0.029155 |
| NCKAP1-liver | NUDT12-muscle | -0.76364 | 0.006233 | 0.036383 |
| PLPP5-liver | NUDT12-muscle | -0.77273 | 0.005299 | 0.035326 |
| PRKX-liver | NUDT12-muscle | -0.79091 | 0.003746 | 0.032221 |
| PRRC2C-liver | NUDT12-muscle | -0.73636 | 0.00976 | 0.043199 |
| RALB-liver | NUDT12-muscle | -0.73636 | 0.00976 | 0.043199 |
| RAP2A-liver | NUDT12-muscle | -0.84545 | 0.001045 | 0.024446 |
| RPS6KA2-liver | NUDT12-muscle | -0.75455 | 0.007282 | 0.038865 |
| SLC25A6-liver | NUDT12-muscle | -0.83636 | 0.001333 | 0.025685 |
| SMIM10-liver | NUDT12-muscle | -0.79091 | 0.003746 | 0.032221 |
| STX7-liver | NUDT12-muscle | -0.88182 | 0.00033 | 0.018341 |
| SUCO-liver | NUDT12-muscle | -0.86364 | 0.000612 | 0.022041 |
| SULF1-liver | NUDT12-muscle | -0.83636 | 0.001333 | 0.025685 |
| TES-liver | NUDT12-muscle | -0.82727 | 0.001677 | 0.02618 |
| TIMP1-liver | NUDT12-muscle | -0.85455 | 0.000807 | 0.022594 |
| TMSB10-liver | NUDT12-muscle | -0.81818 | 0.002083 | 0.027717 |
| TPM4-liver | NUDT12-muscle | -0.76364 | 0.006233 | 0.036383 |
| TSPAN14-liver | NUDT12-muscle | -0.77273 | 0.005299 | 0.035326 |
| VCAN-liver | NUDT12-muscle | -0.85455 | 0.000807 | 0.022594 |
| YWHAZ-liver | NUDT12-muscle | -0.85455 | 0.000807 | 0.022594 |
| ADGRL4-liver | OGDH-muscle | -0.77273 | 0.005299 | 0.035326 |
| ANXA5-liver | OGDH-muscle | -0.72727 | 0.011205 | 0.045908 |
| APP-liver | OGDH-muscle | -0.82727 | 0.001677 | 0.02618 |
| ARHGAP18-liver | OGDH-muscle | -0.73636 | 0.00976 | 0.043199 |
| ARID5B-liver | OGDH-muscle | -0.7 | 0.016471 | 0.052234 |
| CCN4-liver | OGDH-muscle | -0.77273 | 0.005299 | 0.035326 |
| CDH5-liver | OGDH-muscle | -0.74545 | 0.008455 | 0.041717 |
| CLIC1-liver | OGDH-muscle | -0.70909 | 0.014552 | 0.050131 |
| DEFB1-liver | OGDH-muscle | -0.77273 | 0.005299 | 0.035326 |
| DSG2-liver | OGDH-muscle | -0.81818 | 0.002083 | 0.027717 |
| EPS8-liver | OGDH-muscle | -0.75455 | 0.007282 | 0.038865 |
| EZR-liver | OGDH-muscle | -0.70909 | 0.014552 | 0.050131 |
| FNBP1L-liver | OGDH-muscle | -0.75455 | 0.007282 | 0.038865 |
| HEG1-liver | OGDH-muscle | -0.73636 | 0.00976 | 0.043199 |
| IFT57-liver | OGDH-muscle | -0.79091 | 0.003746 | 0.032221 |
| IPO7-liver | OGDH-muscle | -0.72727 | 0.011205 | 0.045908 |
| ITGB1-liver | OGDH-muscle | -0.8 | 0.00311 | 0.031511 |
| KHDRBS1-liver | OGDH-muscle | -0.8 | 0.00311 | 0.031511 |
| KIF3A-liver | OGDH-muscle | -0.71818 | 0.0128 | 0.048396 |
| MFGE8-liver | OGDH-muscle | -0.78182 | 0.004473 | 0.033769 |
| MMGT1-liver | OGDH-muscle | -0.8 | 0.00311 | 0.031511 |
| MYH9-liver | OGDH-muscle | -0.81818 | 0.002083 | 0.027717 |
| NCKAP1-liver | OGDH-muscle | -0.80909 | 0.002559 | 0.029155 |
| PLPP5-liver | OGDH-muscle | -0.77273 | 0.005299 | 0.035326 |
| PRKX-liver | OGDH-muscle | -0.72727 | 0.011205 | 0.045908 |
| SLC25A6-liver | OGDH-muscle | -0.83636 | 0.001333 | 0.025685 |
| SUCO-liver | OGDH-muscle | -0.77273 | 0.005299 | 0.035326 |
| SULF1-liver | OGDH-muscle | -0.70909 | 0.014552 | 0.050131 |
| TES-liver | OGDH-muscle | -0.75455 | 0.007282 | 0.038865 |
| TMSB10-liver | OGDH-muscle | -0.71818 | 0.0128 | 0.048396 |
| TPM4-liver | OGDH-muscle | -0.71818 | 0.0128 | 0.048396 |
| TSPAN14-liver | OGDH-muscle | -0.71818 | 0.0128 | 0.048396 |
| VCAN-liver | OGDH-muscle | -0.75455 | 0.007282 | 0.038865 |
| YWHAZ-liver | OGDH-muscle | -0.72727 | 0.011205 | 0.045908 |
| DSG2-liver | PAIP2B-muscle | -0.7 | 0.016471 | 0.052234 |
| MMGT1-liver | PAIP2B-muscle | -0.74545 | 0.008455 | 0.041717 |
| SLC25A6-liver | PAIP2B-muscle | -0.70909 | 0.014552 | 0.050131 |
| SUCO-liver | PAIP2B-muscle | -0.7 | 0.016471 | 0.052234 |
| ANXA5-liver | PDHX-muscle | -0.74545 | 0.008455 | 0.041717 |
| APP-liver | PDHX-muscle | -0.70909 | 0.014552 | 0.050131 |
| ARHGAP18-liver | PDHX-muscle | -0.72727 | 0.011205 | 0.045908 |
| ARID5B-liver | PDHX-muscle | -0.85455 | 0.000807 | 0.022594 |
| CCN4-liver | PDHX-muscle | -0.7 | 0.016471 | 0.052234 |
| CD34-liver | PDHX-muscle | -0.74545 | 0.008455 | 0.041717 |
| CLIC1-liver | PDHX-muscle | -0.70909 | 0.014552 | 0.050131 |
| DSG2-liver | PDHX-muscle | -0.7 | 0.016471 | 0.052234 |
| EPS8-liver | PDHX-muscle | -0.71818 | 0.0128 | 0.048396 |
| HEG1-liver | PDHX-muscle | -0.71818 | 0.0128 | 0.048396 |
| IFT57-liver | PDHX-muscle | -0.73636 | 0.00976 | 0.043199 |
| MFGE8-liver | PDHX-muscle | -0.84545 | 0.001045 | 0.024446 |
| MMGT1-liver | PDHX-muscle | -0.73636 | 0.00976 | 0.043199 |
| RAP2A-liver | PDHX-muscle | -0.70909 | 0.014552 | 0.050131 |
| SLC25A6-liver | PDHX-muscle | -0.70909 | 0.014552 | 0.050131 |
| STX7-liver | PDHX-muscle | -0.77273 | 0.005299 | 0.035326 |
| SUCO-liver | PDHX-muscle | -0.73636 | 0.00976 | 0.043199 |
| SULF1-liver | PDHX-muscle | -0.70909 | 0.014552 | 0.050131 |
| TIMP1-liver | PDHX-muscle | -0.75455 | 0.007282 | 0.038865 |
| VCAN-liver | PDHX-muscle | -0.75455 | 0.007282 | 0.038865 |
| YWHAZ-liver | PDHX-muscle | -0.73636 | 0.00976 | 0.043199 |
| APP-liver | PEBP4-muscle | -0.72727 | 0.011205 | 0.045908 |
| YWHAZ-liver | PEBP4-muscle | -0.74545 | 0.008455 | 0.041717 |
| ANXA5-liver | PLAA-muscle | -0.73636 | 0.00976 | 0.043199 |
| APP-liver | PLAA-muscle | -0.84545 | 0.001045 | 0.024446 |
| ARHGAP18-liver | PLAA-muscle | -0.71818 | 0.0128 | 0.048396 |
| ARID5B-liver | PLAA-muscle | -0.76364 | 0.006233 | 0.036383 |
| CD34-liver | PLAA-muscle | -0.81818 | 0.002083 | 0.027717 |
| CLIC1-liver | PLAA-muscle | -0.82727 | 0.001677 | 0.02618 |
| DSG2-liver | PLAA-muscle | -0.78182 | 0.004473 | 0.033769 |
| FNBP1L-liver | PLAA-muscle | -0.76364 | 0.006233 | 0.036383 |
| HEG1-liver | PLAA-muscle | -0.78182 | 0.004473 | 0.033769 |
| ITGB1-liver | PLAA-muscle | -0.76364 | 0.006233 | 0.036383 |
| KHDRBS1-liver | PLAA-muscle | -0.70909 | 0.014552 | 0.050131 |
| KIF3A-liver | PLAA-muscle | -0.70909 | 0.014552 | 0.050131 |
| MFGE8-liver | PLAA-muscle | -0.74545 | 0.008455 | 0.041717 |
| MMGT1-liver | PLAA-muscle | -0.73636 | 0.00976 | 0.043199 |
| MYH9-liver | PLAA-muscle | -0.7 | 0.016471 | 0.052234 |
| NCKAP1-liver | PLAA-muscle | -0.71818 | 0.0128 | 0.048396 |
| PLPP5-liver | PLAA-muscle | -0.70909 | 0.014552 | 0.050131 |
| PRKX-liver | PLAA-muscle | -0.73636 | 0.00976 | 0.043199 |
| RAP2A-liver | PLAA-muscle | -0.80909 | 0.002559 | 0.029155 |
| SLC25A6-liver | PLAA-muscle | -0.72727 | 0.011205 | 0.045908 |
| SMIM10-liver | PLAA-muscle | -0.77273 | 0.005299 | 0.035326 |
| STX7-liver | PLAA-muscle | -0.77273 | 0.005299 | 0.035326 |
| SUCO-liver | PLAA-muscle | -0.73636 | 0.00976 | 0.043199 |
| SULF1-liver | PLAA-muscle | -0.82727 | 0.001677 | 0.02618 |
| TES-liver | PLAA-muscle | -0.72727 | 0.011205 | 0.045908 |
| TIMP1-liver | PLAA-muscle | -0.76364 | 0.006233 | 0.036383 |
| TMSB10-liver | PLAA-muscle | -0.77273 | 0.005299 | 0.035326 |
| TPM4-liver | PLAA-muscle | -0.73636 | 0.00976 | 0.043199 |
| VCAN-liver | PLAA-muscle | -0.72727 | 0.011205 | 0.045908 |
| YWHAZ-liver | PLAA-muscle | -0.88182 | 0.00033 | 0.018341 |
| ADGRL4-liver | POLR3K-muscle | -0.70909 | 0.014552 | 0.050131 |
| ANXA5-liver | POLR3K-muscle | -0.84545 | 0.001045 | 0.024446 |
| APP-liver | POLR3K-muscle | -0.83636 | 0.001333 | 0.025685 |
| ARHGAP18-liver | POLR3K-muscle | -0.82727 | 0.001677 | 0.02618 |
| ARID5B-liver | POLR3K-muscle | -0.89091 | 0.000233 | 0.018341 |
| CAMSAP2-liver | POLR3K-muscle | -0.71818 | 0.0128 | 0.048396 |
| CCN4-liver | POLR3K-muscle | -0.8 | 0.00311 | 0.031511 |
| CD34-liver | POLR3K-muscle | -0.88182 | 0.00033 | 0.018341 |
| CDH5-liver | POLR3K-muscle | -0.80909 | 0.002559 | 0.029155 |
| CLIC1-liver | POLR3K-muscle | -0.86364 | 0.000612 | 0.022041 |
| DSG2-liver | POLR3K-muscle | -0.82727 | 0.001677 | 0.02618 |
| EPS8-liver | POLR3K-muscle | -0.82727 | 0.001677 | 0.02618 |
| EZR-liver | POLR3K-muscle | -0.80909 | 0.002559 | 0.029155 |
| FNBP1L-liver | POLR3K-muscle | -0.83636 | 0.001333 | 0.025685 |
| HEG1-liver | POLR3K-muscle | -0.83636 | 0.001333 | 0.025685 |
| IFT57-liver | POLR3K-muscle | -0.76364 | 0.006233 | 0.036383 |
| ITGB1-liver | POLR3K-muscle | -0.79091 | 0.003746 | 0.032221 |
| KHDRBS1-liver | POLR3K-muscle | -0.80909 | 0.002559 | 0.029155 |
| KIF3A-liver | POLR3K-muscle | -0.8 | 0.00311 | 0.031511 |
| MFGE8-liver | POLR3K-muscle | -0.88182 | 0.00033 | 0.018341 |
| MFSD6-liver | POLR3K-muscle | -0.78182 | 0.004473 | 0.033769 |
| MMGT1-liver | POLR3K-muscle | -0.81818 | 0.002083 | 0.027717 |
| MYH9-liver | POLR3K-muscle | -0.77273 | 0.005299 | 0.035326 |
| NCKAP1-liver | POLR3K-muscle | -0.77273 | 0.005299 | 0.035326 |
| PLPP5-liver | POLR3K-muscle | -0.8 | 0.00311 | 0.031511 |
| PRKX-liver | POLR3K-muscle | -0.82727 | 0.001677 | 0.02618 |
| PRRC2C-liver | POLR3K-muscle | -0.76364 | 0.006233 | 0.036383 |
| RALB-liver | POLR3K-muscle | -0.72727 | 0.011205 | 0.045908 |
| RAP2A-liver | POLR3K-muscle | -0.85455 | 0.000807 | 0.022594 |
| RPS6KA2-liver | POLR3K-muscle | -0.74545 | 0.008455 | 0.041717 |
| SLC25A6-liver | POLR3K-muscle | -0.82727 | 0.001677 | 0.02618 |
| SMIM10-liver | POLR3K-muscle | -0.8 | 0.00311 | 0.031511 |
| STX7-liver | POLR3K-muscle | -0.88182 | 0.00033 | 0.018341 |
| SUCO-liver | POLR3K-muscle | -0.84545 | 0.001045 | 0.024446 |
| SULF1-liver | POLR3K-muscle | -0.86364 | 0.000612 | 0.022041 |
| TES-liver | POLR3K-muscle | -0.81818 | 0.002083 | 0.027717 |
| TIMP1-liver | POLR3K-muscle | -0.88182 | 0.00033 | 0.018341 |
| TMSB10-liver | POLR3K-muscle | -0.81818 | 0.002083 | 0.027717 |
| TPM4-liver | POLR3K-muscle | -0.74545 | 0.008455 | 0.041717 |
| TSPAN14-liver | POLR3K-muscle | -0.72727 | 0.011205 | 0.045908 |
| VCAN-liver | POLR3K-muscle | -0.83636 | 0.001333 | 0.025685 |
| YWHAZ-liver | POLR3K-muscle | -0.88182 | 0.00033 | 0.018341 |
| ABI2-liver | PPARA-muscle | -0.79091 | 0.003746 | 0.032221 |
| ADGRL4-liver | PPARA-muscle | -0.88182 | 0.00033 | 0.018341 |
| ALCAM-liver | PPARA-muscle | -0.74545 | 0.008455 | 0.041717 |
| ANXA5-liver | PPARA-muscle | -0.87273 | 0.000455 | 0.020203 |
| AP2B1-liver | PPARA-muscle | -0.76364 | 0.006233 | 0.036383 |
| APP-liver | PPARA-muscle | -0.88182 | 0.00033 | 0.018341 |
| ARHGAP18-liver | PPARA-muscle | -0.92727 | 3.97E-05 | 0.016759 |
| ARID5B-liver | PPARA-muscle | -0.87273 | 0.000455 | 0.020203 |
| ATL3-liver | PPARA-muscle | -0.73636 | 0.00976 | 0.043199 |
| B4GALT5-liver | PPARA-muscle | -0.78182 | 0.004473 | 0.033769 |
| BMPR2-liver | PPARA-muscle | -0.8 | 0.00311 | 0.031511 |
| CAMSAP2-liver | PPARA-muscle | -0.78182 | 0.004473 | 0.033769 |
| CAPNS1-liver | PPARA-muscle | -0.74545 | 0.008455 | 0.041717 |
| CCN4-liver | PPARA-muscle | -0.9 | 0.00016 | 0.016927 |
| CD34-liver | PPARA-muscle | -0.82727 | 0.001677 | 0.02618 |
| CDH5-liver | PPARA-muscle | -0.92727 | 3.97E-05 | 0.016759 |
| CLIC1-liver | PPARA-muscle | -0.83636 | 0.001333 | 0.025685 |
| DEFB1-liver | PPARA-muscle | -0.86364 | 0.000612 | 0.022041 |
| DSG2-liver | PPARA-muscle | -0.90909 | 0.000106 | 0.016759 |
| EPS8-liver | PPARA-muscle | -0.88182 | 0.00033 | 0.018341 |
| EZR-liver | PPARA-muscle | -0.81818 | 0.002083 | 0.027717 |
| FNBP1L-liver | PPARA-muscle | -0.89091 | 0.000233 | 0.018341 |
| HDAC2-liver | PPARA-muscle | -0.82727 | 0.001677 | 0.02618 |
| HEG1-liver | PPARA-muscle | -0.86364 | 0.000612 | 0.022041 |
| IFT57-liver | PPARA-muscle | -0.88182 | 0.00033 | 0.018341 |
| IPO7-liver | PPARA-muscle | -0.73636 | 0.00976 | 0.043199 |
| ITGB1-liver | PPARA-muscle | -0.87273 | 0.000455 | 0.020203 |
| KHDRBS1-liver | PPARA-muscle | -0.88182 | 0.00033 | 0.018341 |
| KIF3A-liver | PPARA-muscle | -0.88182 | 0.00033 | 0.018341 |
| KIF5B-liver | PPARA-muscle | -0.74545 | 0.008455 | 0.041717 |
| MFGE8-liver | PPARA-muscle | -0.90909 | 0.000106 | 0.016759 |
| MFSD6-liver | PPARA-muscle | -0.82727 | 0.001677 | 0.02618 |
| MMGT1-liver | PPARA-muscle | -0.94545 | 1.12E-05 | 0.016759 |
| MTMR6-liver | PPARA-muscle | -0.8 | 0.00311 | 0.031511 |
| MYH9-liver | PPARA-muscle | -0.88182 | 0.00033 | 0.018341 |
| NCKAP1-liver | PPARA-muscle | -0.86364 | 0.000612 | 0.022041 |
| PLPP5-liver | PPARA-muscle | -0.85455 | 0.000807 | 0.022594 |
| PRKX-liver | PPARA-muscle | -0.84545 | 0.001045 | 0.024446 |
| PRRC2C-liver | PPARA-muscle | -0.83636 | 0.001333 | 0.025685 |
| RALB-liver | PPARA-muscle | -0.83636 | 0.001333 | 0.025685 |
| RAP2A-liver | PPARA-muscle | -0.86364 | 0.000612 | 0.022041 |
| RPS6KA2-liver | PPARA-muscle | -0.83636 | 0.001333 | 0.025685 |
| SLC25A6-liver | PPARA-muscle | -0.91818 | 6.66E-05 | 0.016759 |
| SMIM10-liver | PPARA-muscle | -0.81818 | 0.002083 | 0.027717 |
| STX7-liver | PPARA-muscle | -0.88182 | 0.00033 | 0.018341 |
| SUCO-liver | PPARA-muscle | -0.93636 | 2.21E-05 | 0.016759 |
| SULF1-liver | PPARA-muscle | -0.83636 | 0.001333 | 0.025685 |
| TES-liver | PPARA-muscle | -0.89091 | 0.000233 | 0.018341 |
| TIMP1-liver | PPARA-muscle | -0.83636 | 0.001333 | 0.025685 |
| TMSB10-liver | PPARA-muscle | -0.85455 | 0.000807 | 0.022594 |
| TPM4-liver | PPARA-muscle | -0.83636 | 0.001333 | 0.025685 |
| TSPAN14-liver | PPARA-muscle | -0.84545 | 0.001045 | 0.024446 |
| TTF2-liver | PPARA-muscle | -0.70909 | 0.014552 | 0.050131 |
| VCAN-liver | PPARA-muscle | -0.9 | 0.00016 | 0.016927 |
| VEZF1-liver | PPARA-muscle | -0.7 | 0.016471 | 0.052234 |
| WDR1-liver | PPARA-muscle | -0.78182 | 0.004473 | 0.033769 |
| YWHAB-liver | PPARA-muscle | -0.72727 | 0.011205 | 0.045908 |
| YWHAZ-liver | PPARA-muscle | -0.82727 | 0.001677 | 0.02618 |
| APP-liver | PTCD3-muscle | -0.72727 | 0.011205 | 0.045908 |
| DSG2-liver | PTCD3-muscle | -0.74545 | 0.008455 | 0.041717 |
| EPS8-liver | PTCD3-muscle | -0.71818 | 0.0128 | 0.048396 |
| FNBP1L-liver | PTCD3-muscle | -0.7 | 0.016471 | 0.052234 |
| ITGB1-liver | PTCD3-muscle | -0.7 | 0.016471 | 0.052234 |
| KHDRBS1-liver | PTCD3-muscle | -0.7 | 0.016471 | 0.052234 |
| MFGE8-liver | PTCD3-muscle | -0.70909 | 0.014552 | 0.050131 |
| MMGT1-liver | PTCD3-muscle | -0.73636 | 0.00976 | 0.043199 |
| NCKAP1-liver | PTCD3-muscle | -0.76364 | 0.006233 | 0.036383 |
| PLPP5-liver | PTCD3-muscle | -0.74545 | 0.008455 | 0.041717 |
| SLC25A6-liver | PTCD3-muscle | -0.76364 | 0.006233 | 0.036383 |
| SUCO-liver | PTCD3-muscle | -0.71818 | 0.0128 | 0.048396 |
| TTF2-liver | PTCD3-muscle | -0.79091 | 0.003746 | 0.032221 |
| APP-liver | RASL10B-muscle | -0.72727 | 0.011205 | 0.045908 |
| BMPR2-liver | RASL10B-muscle | -0.75455 | 0.007282 | 0.038865 |
| DSG2-liver | RASL10B-muscle | -0.7 | 0.016471 | 0.052234 |
| MMGT1-liver | RASL10B-muscle | -0.71818 | 0.0128 | 0.048396 |
| NCKAP1-liver | RASL10B-muscle | -0.70909 | 0.014552 | 0.050131 |
| TTF2-liver | RASL10B-muscle | -0.7 | 0.016471 | 0.052234 |
| ANXA5-liver | RPL3L-muscle | -0.73636 | 0.00976 | 0.043199 |
| APP-liver | RPL3L-muscle | -0.83636 | 0.001333 | 0.025685 |
| ARHGAP18-liver | RPL3L-muscle | -0.72727 | 0.011205 | 0.045908 |
| BMPR2-liver | RPL3L-muscle | -0.70909 | 0.014552 | 0.050131 |
| CD34-liver | RPL3L-muscle | -0.74545 | 0.008455 | 0.041717 |
| CDH5-liver | RPL3L-muscle | -0.7 | 0.016471 | 0.052234 |
| CLIC1-liver | RPL3L-muscle | -0.77273 | 0.005299 | 0.035326 |
| DEFB1-liver | RPL3L-muscle | -0.72727 | 0.011205 | 0.045908 |
| DSG2-liver | RPL3L-muscle | -0.76364 | 0.006233 | 0.036383 |
| FNBP1L-liver | RPL3L-muscle | -0.71818 | 0.0128 | 0.048396 |
| HEG1-liver | RPL3L-muscle | -0.76364 | 0.006233 | 0.036383 |
| IFT57-liver | RPL3L-muscle | -0.75455 | 0.007282 | 0.038865 |
| ITGB1-liver | RPL3L-muscle | -0.73636 | 0.00976 | 0.043199 |
| KHDRBS1-liver | RPL3L-muscle | -0.75455 | 0.007282 | 0.038865 |
| KIF3A-liver | RPL3L-muscle | -0.72727 | 0.011205 | 0.045908 |
| MFGE8-liver | RPL3L-muscle | -0.70909 | 0.014552 | 0.050131 |
| MMGT1-liver | RPL3L-muscle | -0.77273 | 0.005299 | 0.035326 |
| MYH9-liver | RPL3L-muscle | -0.76364 | 0.006233 | 0.036383 |
| NCKAP1-liver | RPL3L-muscle | -0.72727 | 0.011205 | 0.045908 |
| PRKX-liver | RPL3L-muscle | -0.72727 | 0.011205 | 0.045908 |
| RAP2A-liver | RPL3L-muscle | -0.77273 | 0.005299 | 0.035326 |
| SLC25A6-liver | RPL3L-muscle | -0.73636 | 0.00976 | 0.043199 |
| SMIM10-liver | RPL3L-muscle | -0.73636 | 0.00976 | 0.043199 |
| STX7-liver | RPL3L-muscle | -0.73636 | 0.00976 | 0.043199 |
| SUCO-liver | RPL3L-muscle | -0.73636 | 0.00976 | 0.043199 |
| SULF1-liver | RPL3L-muscle | -0.77273 | 0.005299 | 0.035326 |
| TES-liver | RPL3L-muscle | -0.73636 | 0.00976 | 0.043199 |
| TIMP1-liver | RPL3L-muscle | -0.71818 | 0.0128 | 0.048396 |
| TMSB10-liver | RPL3L-muscle | -0.77273 | 0.005299 | 0.035326 |
| TPM4-liver | RPL3L-muscle | -0.75455 | 0.007282 | 0.038865 |
| TSPAN14-liver | RPL3L-muscle | -0.72727 | 0.011205 | 0.045908 |
| VCAN-liver | RPL3L-muscle | -0.70909 | 0.014552 | 0.050131 |
| YWHAZ-liver | RPL3L-muscle | -0.80909 | 0.002559 | 0.029155 |
| APP-liver | SDHD-muscle | -0.72727 | 0.011205 | 0.045908 |
| ARID5B-liver | SDHD-muscle | -0.70909 | 0.014552 | 0.050131 |
| DSG2-liver | SDHD-muscle | -0.7 | 0.016471 | 0.052234 |
| PLPP5-liver | SDHD-muscle | -0.70909 | 0.014552 | 0.050131 |
| YWHAZ-liver | SDHD-muscle | -0.73636 | 0.00976 | 0.043199 |
| ANXA5-liver | SLC25A11-muscle | -0.7 | 0.016471 | 0.052234 |
| APP-liver | SLC25A11-muscle | -0.84545 | 0.001045 | 0.024446 |
| ARHGAP18-liver | SLC25A11-muscle | -0.77273 | 0.005299 | 0.035326 |
| ARID5B-liver | SLC25A11-muscle | -0.76364 | 0.006233 | 0.036383 |
| BMPR2-liver | SLC25A11-muscle | -0.74545 | 0.008455 | 0.041717 |
| CA5B-liver | SLC25A11-muscle | -0.73636 | 0.00976 | 0.043199 |
| CCN4-liver | SLC25A11-muscle | -0.73636 | 0.00976 | 0.043199 |
| CD34-liver | SLC25A11-muscle | -0.75455 | 0.007282 | 0.038865 |
| CLIC1-liver | SLC25A11-muscle | -0.77273 | 0.005299 | 0.035326 |
| DSG2-liver | SLC25A11-muscle | -0.83636 | 0.001333 | 0.025685 |
| EPS8-liver | SLC25A11-muscle | -0.76364 | 0.006233 | 0.036383 |
| EZR-liver | SLC25A11-muscle | -0.74545 | 0.008455 | 0.041717 |
| FNBP1L-liver | SLC25A11-muscle | -0.80909 | 0.002559 | 0.029155 |
| HEG1-liver | SLC25A11-muscle | -0.75455 | 0.007282 | 0.038865 |
| ITGB1-liver | SLC25A11-muscle | -0.81818 | 0.002083 | 0.027717 |
| KHDRBS1-liver | SLC25A11-muscle | -0.72727 | 0.011205 | 0.045908 |
| KIF3A-liver | SLC25A11-muscle | -0.71818 | 0.0128 | 0.048396 |
| MFGE8-liver | SLC25A11-muscle | -0.76364 | 0.006233 | 0.036383 |
| MMGT1-liver | SLC25A11-muscle | -0.8 | 0.00311 | 0.031511 |
| NCKAP1-liver | SLC25A11-muscle | -0.79091 | 0.003746 | 0.032221 |
| PLPP5-liver | SLC25A11-muscle | -0.80909 | 0.002559 | 0.029155 |
| PRKX-liver | SLC25A11-muscle | -0.73636 | 0.00976 | 0.043199 |
| PRRC2C-liver | SLC25A11-muscle | -0.70909 | 0.014552 | 0.050131 |
| RAP2A-liver | SLC25A11-muscle | -0.79091 | 0.003746 | 0.032221 |
| SLC25A6-liver | SLC25A11-muscle | -0.79091 | 0.003746 | 0.032221 |
| SMIM10-liver | SLC25A11-muscle | -0.75455 | 0.007282 | 0.038865 |
| STX7-liver | SLC25A11-muscle | -0.75455 | 0.007282 | 0.038865 |
| SUCO-liver | SLC25A11-muscle | -0.79091 | 0.003746 | 0.032221 |
| SULF1-liver | SLC25A11-muscle | -0.77273 | 0.005299 | 0.035326 |
| TES-liver | SLC25A11-muscle | -0.73636 | 0.00976 | 0.043199 |
| TIMP1-liver | SLC25A11-muscle | -0.70909 | 0.014552 | 0.050131 |
| TMSB10-liver | SLC25A11-muscle | -0.73636 | 0.00976 | 0.043199 |
| TPM4-liver | SLC25A11-muscle | -0.74545 | 0.008455 | 0.041717 |
| TTF2-liver | SLC25A11-muscle | -0.73636 | 0.00976 | 0.043199 |
| VCAN-liver | SLC25A11-muscle | -0.75455 | 0.007282 | 0.038865 |
| YWHAZ-liver | SLC25A11-muscle | -0.81818 | 0.002083 | 0.027717 |
| ANXA5-liver | SLC25A12-muscle | -0.72727 | 0.011205 | 0.045908 |
| APP-liver | SLC25A12-muscle | -0.77273 | 0.005299 | 0.035326 |
| ARID5B-liver | SLC25A12-muscle | -0.77273 | 0.005299 | 0.035326 |
| CD34-liver | SLC25A12-muscle | -0.8 | 0.00311 | 0.031511 |
| CLIC1-liver | SLC25A12-muscle | -0.78182 | 0.004473 | 0.033769 |
| DSG2-liver | SLC25A12-muscle | -0.7 | 0.016471 | 0.052234 |
| HEG1-liver | SLC25A12-muscle | -0.72727 | 0.011205 | 0.045908 |
| MFGE8-liver | SLC25A12-muscle | -0.74545 | 0.008455 | 0.041717 |
| RAP2A-liver | SLC25A12-muscle | -0.75455 | 0.007282 | 0.038865 |
| SMIM10-liver | SLC25A12-muscle | -0.7 | 0.016471 | 0.052234 |
| STX7-liver | SLC25A12-muscle | -0.76364 | 0.006233 | 0.036383 |
| SULF1-liver | SLC25A12-muscle | -0.78182 | 0.004473 | 0.033769 |
| TIMP1-liver | SLC25A12-muscle | -0.76364 | 0.006233 | 0.036383 |
| TMSB10-liver | SLC25A12-muscle | -0.71818 | 0.0128 | 0.048396 |
| YWHAZ-liver | SLC25A12-muscle | -0.83636 | 0.001333 | 0.025685 |
| ABI2-liver | SLC25A4-muscle | -0.72727 | 0.011205 | 0.045908 |
| ADGRL4-liver | SLC25A4-muscle | -0.85455 | 0.000807 | 0.022594 |
| ALCAM-liver | SLC25A4-muscle | -0.8 | 0.00311 | 0.031511 |
| ANXA5-liver | SLC25A4-muscle | -0.81818 | 0.002083 | 0.027717 |
| AP2B1-liver | SLC25A4-muscle | -0.7 | 0.016471 | 0.052234 |
| APP-liver | SLC25A4-muscle | -0.89091 | 0.000233 | 0.018341 |
| ARHGAP18-liver | SLC25A4-muscle | -0.87273 | 0.000455 | 0.020203 |
| ARID5B-liver | SLC25A4-muscle | -0.78182 | 0.004473 | 0.033769 |
| B4GALT5-liver | SLC25A4-muscle | -0.74545 | 0.008455 | 0.041717 |
| BMPR2-liver | SLC25A4-muscle | -0.77273 | 0.005299 | 0.035326 |
| CA5B-liver | SLC25A4-muscle | -0.72727 | 0.011205 | 0.045908 |
| CAMSAP2-liver | SLC25A4-muscle | -0.71818 | 0.0128 | 0.048396 |
| CCN4-liver | SLC25A4-muscle | -0.82727 | 0.001677 | 0.02618 |
| CD34-liver | SLC25A4-muscle | -0.77273 | 0.005299 | 0.035326 |
| CDH5-liver | SLC25A4-muscle | -0.83636 | 0.001333 | 0.025685 |
| CLIC1-liver | SLC25A4-muscle | -0.78182 | 0.004473 | 0.033769 |
| DEFB1-liver | SLC25A4-muscle | -0.89091 | 0.000233 | 0.018341 |
| DSG2-liver | SLC25A4-muscle | -0.89091 | 0.000233 | 0.018341 |
| EPS8-liver | SLC25A4-muscle | -0.79091 | 0.003746 | 0.032221 |
| EZR-liver | SLC25A4-muscle | -0.72727 | 0.011205 | 0.045908 |
| FNBP1L-liver | SLC25A4-muscle | -0.85455 | 0.000807 | 0.022594 |
| HDAC2-liver | SLC25A4-muscle | -0.78182 | 0.004473 | 0.033769 |
| HEG1-liver | SLC25A4-muscle | -0.76364 | 0.006233 | 0.036383 |
| IFT57-liver | SLC25A4-muscle | -0.80909 | 0.002559 | 0.029155 |
| IPO7-liver | SLC25A4-muscle | -0.77273 | 0.005299 | 0.035326 |
| ITGB1-liver | SLC25A4-muscle | -0.85455 | 0.000807 | 0.022594 |
| KHDRBS1-liver | SLC25A4-muscle | -0.86364 | 0.000612 | 0.022041 |
| KIF3A-liver | SLC25A4-muscle | -0.84545 | 0.001045 | 0.024446 |
| MFGE8-liver | SLC25A4-muscle | -0.82727 | 0.001677 | 0.02618 |
| MFSD6-liver | SLC25A4-muscle | -0.79091 | 0.003746 | 0.032221 |
| MMGT1-liver | SLC25A4-muscle | -0.90909 | 0.000106 | 0.016759 |
| MTMR6-liver | SLC25A4-muscle | -0.72727 | 0.011205 | 0.045908 |
| MYH9-liver | SLC25A4-muscle | -0.84545 | 0.001045 | 0.024446 |
| NCKAP1-liver | SLC25A4-muscle | -0.87273 | 0.000455 | 0.020203 |
| PLPP5-liver | SLC25A4-muscle | -0.76364 | 0.006233 | 0.036383 |
| PRKX-liver | SLC25A4-muscle | -0.80909 | 0.002559 | 0.029155 |
| PRRC2C-liver | SLC25A4-muscle | -0.8 | 0.00311 | 0.031511 |
| RALB-liver | SLC25A4-muscle | -0.82727 | 0.001677 | 0.02618 |
| RAP2A-liver | SLC25A4-muscle | -0.81818 | 0.002083 | 0.027717 |
| RPS6KA2-liver | SLC25A4-muscle | -0.79091 | 0.003746 | 0.032221 |
| SLC25A6-liver | SLC25A4-muscle | -0.9 | 0.00016 | 0.016927 |
| SMIM10-liver | SLC25A4-muscle | -0.76364 | 0.006233 | 0.036383 |
| STX7-liver | SLC25A4-muscle | -0.84545 | 0.001045 | 0.024446 |
| SUCO-liver | SLC25A4-muscle | -0.89091 | 0.000233 | 0.018341 |
| SULF1-liver | SLC25A4-muscle | -0.78182 | 0.004473 | 0.033769 |
| TES-liver | SLC25A4-muscle | -0.86364 | 0.000612 | 0.022041 |
| TIMP1-liver | SLC25A4-muscle | -0.78182 | 0.004473 | 0.033769 |
| TMSB10-liver | SLC25A4-muscle | -0.79091 | 0.003746 | 0.032221 |
| TPM4-liver | SLC25A4-muscle | -0.80909 | 0.002559 | 0.029155 |
| TSPAN14-liver | SLC25A4-muscle | -0.78182 | 0.004473 | 0.033769 |
| TTF2-liver | SLC25A4-muscle | -0.82727 | 0.001677 | 0.02618 |
| VCAN-liver | SLC25A4-muscle | -0.79091 | 0.003746 | 0.032221 |
| WDR1-liver | SLC25A4-muscle | -0.72727 | 0.011205 | 0.045908 |
| YWHAZ-liver | SLC25A4-muscle | -0.79091 | 0.003746 | 0.032221 |
| ADGRL4-liver | SMCO1-muscle | -0.78182 | 0.004473 | 0.033769 |
| ANXA5-liver | SMCO1-muscle | -0.85455 | 0.000807 | 0.022594 |
| APP-liver | SMCO1-muscle | -0.85455 | 0.000807 | 0.022594 |
| ARHGAP18-liver | SMCO1-muscle | -0.8 | 0.00311 | 0.031511 |
| ARID5B-liver | SMCO1-muscle | -0.81818 | 0.002083 | 0.027717 |
| CAMSAP2-liver | SMCO1-muscle | -0.75455 | 0.007282 | 0.038865 |
| CAPNS1-liver | SMCO1-muscle | -0.7 | 0.016471 | 0.052234 |
| CCN4-liver | SMCO1-muscle | -0.79091 | 0.003746 | 0.032221 |
| CD34-liver | SMCO1-muscle | -0.80909 | 0.002559 | 0.029155 |
| CDH5-liver | SMCO1-muscle | -0.76364 | 0.006233 | 0.036383 |
| CLIC1-liver | SMCO1-muscle | -0.81818 | 0.002083 | 0.027717 |
| DEFB1-liver | SMCO1-muscle | -0.81818 | 0.002083 | 0.027717 |
| DSG2-liver | SMCO1-muscle | -0.81818 | 0.002083 | 0.027717 |
| EPS8-liver | SMCO1-muscle | -0.75455 | 0.007282 | 0.038865 |
| FNBP1L-liver | SMCO1-muscle | -0.78182 | 0.004473 | 0.033769 |
| HDAC2-liver | SMCO1-muscle | -0.70909 | 0.014552 | 0.050131 |
| HEG1-liver | SMCO1-muscle | -0.83636 | 0.001333 | 0.025685 |
| IFT57-liver | SMCO1-muscle | -0.88182 | 0.00033 | 0.018341 |
| ITGB1-liver | SMCO1-muscle | -0.78182 | 0.004473 | 0.033769 |
| KHDRBS1-liver | SMCO1-muscle | -0.82727 | 0.001677 | 0.02618 |
| KIF3A-liver | SMCO1-muscle | -0.80909 | 0.002559 | 0.029155 |
| MFGE8-liver | SMCO1-muscle | -0.86364 | 0.000612 | 0.022041 |
| MMGT1-liver | SMCO1-muscle | -0.83636 | 0.001333 | 0.025685 |
| MYH9-liver | SMCO1-muscle | -0.88182 | 0.00033 | 0.018341 |
| NCKAP1-liver | SMCO1-muscle | -0.76364 | 0.006233 | 0.036383 |
| PLPP5-liver | SMCO1-muscle | -0.72727 | 0.011205 | 0.045908 |
| PRKX-liver | SMCO1-muscle | -0.77273 | 0.005299 | 0.035326 |
| RALB-liver | SMCO1-muscle | -0.75455 | 0.007282 | 0.038865 |
| RAP2A-liver | SMCO1-muscle | -0.78182 | 0.004473 | 0.033769 |
| RPS6KA2-liver | SMCO1-muscle | -0.71818 | 0.0128 | 0.048396 |
| SLC25A6-liver | SMCO1-muscle | -0.82727 | 0.001677 | 0.02618 |
| SMIM10-liver | SMCO1-muscle | -0.72727 | 0.011205 | 0.045908 |
| STX7-liver | SMCO1-muscle | -0.80909 | 0.002559 | 0.029155 |
| SUCO-liver | SMCO1-muscle | -0.81818 | 0.002083 | 0.027717 |
| SULF1-liver | SMCO1-muscle | -0.81818 | 0.002083 | 0.027717 |
| TES-liver | SMCO1-muscle | -0.82727 | 0.001677 | 0.02618 |
| TIMP1-liver | SMCO1-muscle | -0.81818 | 0.002083 | 0.027717 |
| TMSB10-liver | SMCO1-muscle | -0.82727 | 0.001677 | 0.02618 |
| TPM4-liver | SMCO1-muscle | -0.77273 | 0.005299 | 0.035326 |
| TSPAN14-liver | SMCO1-muscle | -0.81818 | 0.002083 | 0.027717 |
| VCAN-liver | SMCO1-muscle | -0.82727 | 0.001677 | 0.02618 |
| YWHAZ-liver | SMCO1-muscle | -0.82727 | 0.001677 | 0.02618 |
| APP-liver | SPAG7-muscle | -0.73636 | 0.00976 | 0.043199 |
| DSG2-liver | SPAG7-muscle | -0.70909 | 0.014552 | 0.050131 |
| PLPP5-liver | SPAG7-muscle | -0.7 | 0.016471 | 0.052234 |
| TTF2-liver | SPAG7-muscle | -0.72727 | 0.011205 | 0.045908 |
| APP-liver | SUPV3L1-muscle | -0.71818 | 0.0128 | 0.048396 |
| DSG2-liver | SUPV3L1-muscle | -0.71818 | 0.0128 | 0.048396 |
| MMGT1-liver | SUPV3L1-muscle | -0.72727 | 0.011205 | 0.045908 |
| SLC25A6-liver | SUPV3L1-muscle | -0.7 | 0.016471 | 0.052234 |
| ADGRL4-liver | TACO1-muscle | -0.73636 | 0.00976 | 0.043199 |
| ANXA5-liver | TACO1-muscle | -0.72727 | 0.011205 | 0.045908 |
| APP-liver | TACO1-muscle | -0.84545 | 0.001045 | 0.024446 |
| ARHGAP18-liver | TACO1-muscle | -0.81818 | 0.002083 | 0.027717 |
| ARID5B-liver | TACO1-muscle | -0.78182 | 0.004473 | 0.033769 |
| BMPR2-liver | TACO1-muscle | -0.78182 | 0.004473 | 0.033769 |
| CCN4-liver | TACO1-muscle | -0.78182 | 0.004473 | 0.033769 |
| CD34-liver | TACO1-muscle | -0.73636 | 0.00976 | 0.043199 |
| CDH5-liver | TACO1-muscle | -0.76364 | 0.006233 | 0.036383 |
| CLIC1-liver | TACO1-muscle | -0.76364 | 0.006233 | 0.036383 |
| DEFB1-liver | TACO1-muscle | -0.73636 | 0.00976 | 0.043199 |
| DSG2-liver | TACO1-muscle | -0.85455 | 0.000807 | 0.022594 |
| EPS8-liver | TACO1-muscle | -0.8 | 0.00311 | 0.031511 |
| EZR-liver | TACO1-muscle | -0.77273 | 0.005299 | 0.035326 |
| FNBP1L-liver | TACO1-muscle | -0.81818 | 0.002083 | 0.027717 |
| HEG1-liver | TACO1-muscle | -0.79091 | 0.003746 | 0.032221 |
| IFT57-liver | TACO1-muscle | -0.76364 | 0.006233 | 0.036383 |
| IPO7-liver | TACO1-muscle | -0.7 | 0.016471 | 0.052234 |
| ITGB1-liver | TACO1-muscle | -0.82727 | 0.001677 | 0.02618 |
| KHDRBS1-liver | TACO1-muscle | -0.77273 | 0.005299 | 0.035326 |
| KIF3A-liver | TACO1-muscle | -0.75455 | 0.007282 | 0.038865 |
| KIF5B-liver | TACO1-muscle | -0.71818 | 0.0128 | 0.048396 |
| MFGE8-liver | TACO1-muscle | -0.8 | 0.00311 | 0.031511 |
| MMGT1-liver | TACO1-muscle | -0.85455 | 0.000807 | 0.022594 |
| MYH9-liver | TACO1-muscle | -0.74545 | 0.008455 | 0.041717 |
| NCKAP1-liver | TACO1-muscle | -0.81818 | 0.002083 | 0.027717 |
| PLPP5-liver | TACO1-muscle | -0.83636 | 0.001333 | 0.025685 |
| PRKX-liver | TACO1-muscle | -0.75455 | 0.007282 | 0.038865 |
| PRRC2C-liver | TACO1-muscle | -0.72727 | 0.011205 | 0.045908 |
| RAP2A-liver | TACO1-muscle | -0.79091 | 0.003746 | 0.032221 |
| RPS6KA2-liver | TACO1-muscle | -0.7 | 0.016471 | 0.052234 |
| SLC25A6-liver | TACO1-muscle | -0.82727 | 0.001677 | 0.02618 |
| SMIM10-liver | TACO1-muscle | -0.75455 | 0.007282 | 0.038865 |
| STX7-liver | TACO1-muscle | -0.75455 | 0.007282 | 0.038865 |
| SUCO-liver | TACO1-muscle | -0.82727 | 0.001677 | 0.02618 |
| SULF1-liver | TACO1-muscle | -0.76364 | 0.006233 | 0.036383 |
| TES-liver | TACO1-muscle | -0.76364 | 0.006233 | 0.036383 |
| TIMP1-liver | TACO1-muscle | -0.70909 | 0.014552 | 0.050131 |
| TMSB10-liver | TACO1-muscle | -0.76364 | 0.006233 | 0.036383 |
| TPM4-liver | TACO1-muscle | -0.77273 | 0.005299 | 0.035326 |
| TSPAN14-liver | TACO1-muscle | -0.70909 | 0.014552 | 0.050131 |
| TTF2-liver | TACO1-muscle | -0.70909 | 0.014552 | 0.050131 |
| VCAN-liver | TACO1-muscle | -0.80909 | 0.002559 | 0.029155 |
| YWHAZ-liver | TACO1-muscle | -0.79091 | 0.003746 | 0.032221 |
| TTF2-liver | TADA2B-muscle | -0.73636 | 0.00976 | 0.043199 |
| APP-liver | TBC1D4-muscle | -0.75455 | 0.007282 | 0.038865 |
| ARID5B-liver | TBC1D4-muscle | -0.73636 | 0.00976 | 0.043199 |
| CD34-liver | TBC1D4-muscle | -0.70909 | 0.014552 | 0.050131 |
| DSG2-liver | TBC1D4-muscle | -0.72727 | 0.011205 | 0.045908 |
| FNBP1L-liver | TBC1D4-muscle | -0.70909 | 0.014552 | 0.050131 |
| MFGE8-liver | TBC1D4-muscle | -0.72727 | 0.011205 | 0.045908 |
| MMGT1-liver | TBC1D4-muscle | -0.70909 | 0.014552 | 0.050131 |
| RAP2A-liver | TBC1D4-muscle | -0.70909 | 0.014552 | 0.050131 |
| SLC25A6-liver | TBC1D4-muscle | -0.70909 | 0.014552 | 0.050131 |
| STX7-liver | TBC1D4-muscle | -0.75455 | 0.007282 | 0.038865 |
| SUCO-liver | TBC1D4-muscle | -0.71818 | 0.0128 | 0.048396 |
| YWHAZ-liver | TBC1D4-muscle | -0.73636 | 0.00976 | 0.043199 |
| ARHGAP18-liver | TBX15-muscle | -0.7 | 0.016471 | 0.052234 |
| BCL2L11-liver | TBX15-muscle | -0.70909 | 0.014552 | 0.050131 |
| CCN4-liver | TBX15-muscle | -0.72727 | 0.011205 | 0.045908 |
| CDH5-liver | TBX15-muscle | -0.7 | 0.016471 | 0.052234 |
| DSG2-liver | TBX15-muscle | -0.72727 | 0.011205 | 0.045908 |
| EPS8-liver | TBX15-muscle | -0.77273 | 0.005299 | 0.035326 |
| KHDRBS1-liver | TBX15-muscle | -0.7 | 0.016471 | 0.052234 |
| MFGE8-liver | TBX15-muscle | -0.72727 | 0.011205 | 0.045908 |
| MMGT1-liver | TBX15-muscle | -0.73636 | 0.00976 | 0.043199 |
| NCKAP1-liver | TBX15-muscle | -0.74545 | 0.008455 | 0.041717 |
| PLPP5-liver | TBX15-muscle | -0.8 | 0.00311 | 0.031511 |
| PRRC2C-liver | TBX15-muscle | -0.73636 | 0.00976 | 0.043199 |
| SLC25A6-liver | TBX15-muscle | -0.76364 | 0.006233 | 0.036383 |
| SUCO-liver | TBX15-muscle | -0.72727 | 0.011205 | 0.045908 |
| TTF2-liver | TBX15-muscle | -0.78182 | 0.004473 | 0.033769 |
| VCAN-liver | TBX15-muscle | -0.7 | 0.016471 | 0.052234 |
| ARHGAP18-liver | TECRL-muscle | -0.7 | 0.016471 | 0.052234 |
| BCL2L11-liver | TECRL-muscle | -0.70909 | 0.014552 | 0.050131 |
| CCN4-liver | TECRL-muscle | -0.73636 | 0.00976 | 0.043199 |
| DSG2-liver | TECRL-muscle | -0.73636 | 0.00976 | 0.043199 |
| EPS8-liver | TECRL-muscle | -0.76364 | 0.006233 | 0.036383 |
| FNBP1L-liver | TECRL-muscle | -0.7 | 0.016471 | 0.052234 |
| ITGB1-liver | TECRL-muscle | -0.70909 | 0.014552 | 0.050131 |
| MFGE8-liver | TECRL-muscle | -0.73636 | 0.00976 | 0.043199 |
| MMGT1-liver | TECRL-muscle | -0.73636 | 0.00976 | 0.043199 |
| NCKAP1-liver | TECRL-muscle | -0.7 | 0.016471 | 0.052234 |
| PLPP5-liver | TECRL-muscle | -0.80909 | 0.002559 | 0.029155 |
| SLC25A6-liver | TECRL-muscle | -0.73636 | 0.00976 | 0.043199 |
| SUCO-liver | TECRL-muscle | -0.72727 | 0.011205 | 0.045908 |
| TTF2-liver | TECRL-muscle | -0.74545 | 0.008455 | 0.041717 |
| VCAN-liver | TECRL-muscle | -0.70909 | 0.014552 | 0.050131 |
| ANKRD27-liver | TIMM17A-muscle | -0.70909 | 0.014552 | 0.050131 |
| APP-liver | TIMM17A-muscle | -0.8 | 0.00311 | 0.031511 |
| ARHGAP18-liver | TIMM17A-muscle | -0.76364 | 0.006233 | 0.036383 |
| ARID5B-liver | TIMM17A-muscle | -0.72727 | 0.011205 | 0.045908 |
| BMPR2-liver | TIMM17A-muscle | -0.78182 | 0.004473 | 0.033769 |
| CCN4-liver | TIMM17A-muscle | -0.71818 | 0.0128 | 0.048396 |
| CD34-liver | TIMM17A-muscle | -0.73636 | 0.00976 | 0.043199 |
| CDH5-liver | TIMM17A-muscle | -0.71818 | 0.0128 | 0.048396 |
| CLIC1-liver | TIMM17A-muscle | -0.77273 | 0.005299 | 0.035326 |
| DSG2-liver | TIMM17A-muscle | -0.8 | 0.00311 | 0.031511 |
| EPS8-liver | TIMM17A-muscle | -0.75455 | 0.007282 | 0.038865 |
| EZR-liver | TIMM17A-muscle | -0.76364 | 0.006233 | 0.036383 |
| FNBP1L-liver | TIMM17A-muscle | -0.78182 | 0.004473 | 0.033769 |
| HEG1-liver | TIMM17A-muscle | -0.79091 | 0.003746 | 0.032221 |
| ITGB1-liver | TIMM17A-muscle | -0.79091 | 0.003746 | 0.032221 |
| KHDRBS1-liver | TIMM17A-muscle | -0.7 | 0.016471 | 0.052234 |
| KIF3A-liver | TIMM17A-muscle | -0.7 | 0.016471 | 0.052234 |
| KIF5B-liver | TIMM17A-muscle | -0.71818 | 0.0128 | 0.048396 |
| MFGE8-liver | TIMM17A-muscle | -0.72727 | 0.011205 | 0.045908 |
| MMGT1-liver | TIMM17A-muscle | -0.78182 | 0.004473 | 0.033769 |
| NCKAP1-liver | TIMM17A-muscle | -0.74545 | 0.008455 | 0.041717 |
| PLPP5-liver | TIMM17A-muscle | -0.80909 | 0.002559 | 0.029155 |
| PRKX-liver | TIMM17A-muscle | -0.72727 | 0.011205 | 0.045908 |
| RAP2A-liver | TIMM17A-muscle | -0.79091 | 0.003746 | 0.032221 |
| SLC25A6-liver | TIMM17A-muscle | -0.74545 | 0.008455 | 0.041717 |
| SMIM10-liver | TIMM17A-muscle | -0.77273 | 0.005299 | 0.035326 |
| STX7-liver | TIMM17A-muscle | -0.71818 | 0.0128 | 0.048396 |
| SUCO-liver | TIMM17A-muscle | -0.77273 | 0.005299 | 0.035326 |
| SULF1-liver | TIMM17A-muscle | -0.77273 | 0.005299 | 0.035326 |
| TES-liver | TIMM17A-muscle | -0.70909 | 0.014552 | 0.050131 |
| TMSB10-liver | TIMM17A-muscle | -0.75455 | 0.007282 | 0.038865 |
| TPM4-liver | TIMM17A-muscle | -0.74545 | 0.008455 | 0.041717 |
| VCAN-liver | TIMM17A-muscle | -0.77273 | 0.005299 | 0.035326 |
| YWHAZ-liver | TIMM17A-muscle | -0.8 | 0.00311 | 0.031511 |
| ADGRL4-liver | TMEM143-muscle | -0.75455 | 0.007282 | 0.038865 |
| ANKRD27-liver | TMEM143-muscle | -0.7 | 0.016471 | 0.052234 |
| ANXA5-liver | TMEM143-muscle | -0.76364 | 0.006233 | 0.036383 |
| APP-liver | TMEM143-muscle | -0.88182 | 0.00033 | 0.018341 |
| ARHGAP18-liver | TMEM143-muscle | -0.82727 | 0.001677 | 0.02618 |
| ARID5B-liver | TMEM143-muscle | -0.83636 | 0.001333 | 0.025685 |
| B4GALT5-liver | TMEM143-muscle | -0.73636 | 0.00976 | 0.043199 |
| BMPR2-liver | TMEM143-muscle | -0.78182 | 0.004473 | 0.033769 |
| CA5B-liver | TMEM143-muscle | -0.74545 | 0.008455 | 0.041717 |
| CCN4-liver | TMEM143-muscle | -0.80909 | 0.002559 | 0.029155 |
| CD34-liver | TMEM143-muscle | -0.8 | 0.00311 | 0.031511 |
| CLIC1-liver | TMEM143-muscle | -0.81818 | 0.002083 | 0.027717 |
| DEFB1-liver | TMEM143-muscle | -0.74545 | 0.008455 | 0.041717 |
| DSG2-liver | TMEM143-muscle | -0.88182 | 0.00033 | 0.018341 |
| EPS8-liver | TMEM143-muscle | -0.81818 | 0.002083 | 0.027717 |
| EZR-liver | TMEM143-muscle | -0.79091 | 0.003746 | 0.032221 |
| FNBP1L-liver | TMEM143-muscle | -0.85455 | 0.000807 | 0.022594 |
| HDAC2-liver | TMEM143-muscle | -0.71818 | 0.0128 | 0.048396 |
| HEG1-liver | TMEM143-muscle | -0.82727 | 0.001677 | 0.02618 |
| IFT57-liver | TMEM143-muscle | -0.78182 | 0.004473 | 0.033769 |
| ITGB1-liver | TMEM143-muscle | -0.86364 | 0.000612 | 0.022041 |
| KHDRBS1-liver | TMEM143-muscle | -0.77273 | 0.005299 | 0.035326 |
| KIF3A-liver | TMEM143-muscle | -0.78182 | 0.004473 | 0.033769 |
| KIF5B-liver | TMEM143-muscle | -0.75455 | 0.007282 | 0.038865 |
| MFGE8-liver | TMEM143-muscle | -0.83636 | 0.001333 | 0.025685 |
| MFSD6-liver | TMEM143-muscle | -0.70909 | 0.014552 | 0.050131 |
| MMGT1-liver | TMEM143-muscle | -0.85455 | 0.000807 | 0.022594 |
| MYH9-liver | TMEM143-muscle | -0.76364 | 0.006233 | 0.036383 |
| NCKAP1-liver | TMEM143-muscle | -0.81818 | 0.002083 | 0.027717 |
| PLPP5-liver | TMEM143-muscle | -0.85455 | 0.000807 | 0.022594 |
| PRKX-liver | TMEM143-muscle | -0.77273 | 0.005299 | 0.035326 |
| PRRC2C-liver | TMEM143-muscle | -0.74545 | 0.008455 | 0.041717 |
| RALB-liver | TMEM143-muscle | -0.7 | 0.016471 | 0.052234 |
| RAP2A-liver | TMEM143-muscle | -0.82727 | 0.001677 | 0.02618 |
| RPS6KA2-liver | TMEM143-muscle | -0.73636 | 0.00976 | 0.043199 |
| SLC25A6-liver | TMEM143-muscle | -0.83636 | 0.001333 | 0.025685 |
| SMIM10-liver | TMEM143-muscle | -0.8 | 0.00311 | 0.031511 |
| STX7-liver | TMEM143-muscle | -0.79091 | 0.003746 | 0.032221 |
| SUCO-liver | TMEM143-muscle | -0.83636 | 0.001333 | 0.025685 |
| SULF1-liver | TMEM143-muscle | -0.81818 | 0.002083 | 0.027717 |
| TES-liver | TMEM143-muscle | -0.79091 | 0.003746 | 0.032221 |
| TIMP1-liver | TMEM143-muscle | -0.75455 | 0.007282 | 0.038865 |
| TMSB10-liver | TMEM143-muscle | -0.8 | 0.00311 | 0.031511 |
| TPM4-liver | TMEM143-muscle | -0.80909 | 0.002559 | 0.029155 |
| TSPAN14-liver | TMEM143-muscle | -0.71818 | 0.0128 | 0.048396 |
| TTF2-liver | TMEM143-muscle | -0.71818 | 0.0128 | 0.048396 |
| VCAN-liver | TMEM143-muscle | -0.83636 | 0.001333 | 0.025685 |
| YWHAZ-liver | TMEM143-muscle | -0.85455 | 0.000807 | 0.022594 |
| ANXA5-liver | TOB1-muscle | -0.81818 | 0.002083 | 0.027717 |
| APP-liver | TOB1-muscle | -0.81818 | 0.002083 | 0.027717 |
| ARHGAP18-liver | TOB1-muscle | -0.71818 | 0.0128 | 0.048396 |
| ARID5B-liver | TOB1-muscle | -0.82727 | 0.001677 | 0.02618 |
| CCN4-liver | TOB1-muscle | -0.7 | 0.016471 | 0.052234 |
| CD34-liver | TOB1-muscle | -0.85455 | 0.000807 | 0.022594 |
| CLIC1-liver | TOB1-muscle | -0.83636 | 0.001333 | 0.025685 |
| DSG2-liver | TOB1-muscle | -0.75455 | 0.007282 | 0.038865 |
| EPS8-liver | TOB1-muscle | -0.70909 | 0.014552 | 0.050131 |
| FNBP1L-liver | TOB1-muscle | -0.74545 | 0.008455 | 0.041717 |
| HEG1-liver | TOB1-muscle | -0.79091 | 0.003746 | 0.032221 |
| IFT57-liver | TOB1-muscle | -0.74545 | 0.008455 | 0.041717 |
| ITGB1-liver | TOB1-muscle | -0.71818 | 0.0128 | 0.048396 |
| KHDRBS1-liver | TOB1-muscle | -0.76364 | 0.006233 | 0.036383 |
| KIF3A-liver | TOB1-muscle | -0.73636 | 0.00976 | 0.043199 |
| MFGE8-liver | TOB1-muscle | -0.82727 | 0.001677 | 0.02618 |
| MMGT1-liver | TOB1-muscle | -0.72727 | 0.011205 | 0.045908 |
| MYH9-liver | TOB1-muscle | -0.76364 | 0.006233 | 0.036383 |
| NCKAP1-liver | TOB1-muscle | -0.7 | 0.016471 | 0.052234 |
| PRKX-liver | TOB1-muscle | -0.76364 | 0.006233 | 0.036383 |
| RAP2A-liver | TOB1-muscle | -0.78182 | 0.004473 | 0.033769 |
| SLC25A6-liver | TOB1-muscle | -0.75455 | 0.007282 | 0.038865 |
| SMIM10-liver | TOB1-muscle | -0.71818 | 0.0128 | 0.048396 |
| STX7-liver | TOB1-muscle | -0.81818 | 0.002083 | 0.027717 |
| SUCO-liver | TOB1-muscle | -0.74545 | 0.008455 | 0.041717 |
| SULF1-liver | TOB1-muscle | -0.83636 | 0.001333 | 0.025685 |
| TES-liver | TOB1-muscle | -0.76364 | 0.006233 | 0.036383 |
| TIMP1-liver | TOB1-muscle | -0.85455 | 0.000807 | 0.022594 |
| TMSB10-liver | TOB1-muscle | -0.78182 | 0.004473 | 0.033769 |
| VCAN-liver | TOB1-muscle | -0.75455 | 0.007282 | 0.038865 |
| YWHAZ-liver | TOB1-muscle | -0.87273 | 0.000455 | 0.020203 |
| TTF2-liver | TOP2B-muscle | -0.76364 | 0.006233 | 0.036383 |
| APP-liver | TRMT1L-muscle | -0.7 | 0.016471 | 0.052234 |
| ARHGAP18-liver | TRMT1L-muscle | -0.70909 | 0.014552 | 0.050131 |
| ARID5B-liver | TRMT1L-muscle | -0.70909 | 0.014552 | 0.050131 |
| CLIC1-liver | TRMT1L-muscle | -0.7 | 0.016471 | 0.052234 |
| DSG2-liver | TRMT1L-muscle | -0.73636 | 0.00976 | 0.043199 |
| EPS8-liver | TRMT1L-muscle | -0.78182 | 0.004473 | 0.033769 |
| EZR-liver | TRMT1L-muscle | -0.74545 | 0.008455 | 0.041717 |
| FNBP1L-liver | TRMT1L-muscle | -0.74545 | 0.008455 | 0.041717 |
| ITGB1-liver | TRMT1L-muscle | -0.70909 | 0.014552 | 0.050131 |
| MFGE8-liver | TRMT1L-muscle | -0.7 | 0.016471 | 0.052234 |
| MMGT1-liver | TRMT1L-muscle | -0.7 | 0.016471 | 0.052234 |
| NCKAP1-liver | TRMT1L-muscle | -0.71818 | 0.0128 | 0.048396 |
| PLPP5-liver | TRMT1L-muscle | -0.83636 | 0.001333 | 0.025685 |
| PRRC2C-liver | TRMT1L-muscle | -0.73636 | 0.00976 | 0.043199 |
| RAP2A-liver | TRMT1L-muscle | -0.71818 | 0.0128 | 0.048396 |
| SLC25A6-liver | TRMT1L-muscle | -0.70909 | 0.014552 | 0.050131 |
| SUCO-liver | TRMT1L-muscle | -0.72727 | 0.011205 | 0.045908 |
| SULF1-liver | TRMT1L-muscle | -0.7 | 0.016471 | 0.052234 |
| TTF2-liver | TRMT1L-muscle | -0.7 | 0.016471 | 0.052234 |
| VCAN-liver | TRMT1L-muscle | -0.70909 | 0.014552 | 0.050131 |
| YWHAZ-liver | TRMT1L-muscle | -0.71818 | 0.0128 | 0.048396 |
| APP-liver | UBE2D1-muscle | -0.8 | 0.00311 | 0.031511 |
| BMPR2-liver | UBE2D1-muscle | -0.7 | 0.016471 | 0.052234 |
| DEFB1-liver | UBE2D1-muscle | -0.73636 | 0.00976 | 0.043199 |
| DSG2-liver | UBE2D1-muscle | -0.74545 | 0.008455 | 0.041717 |
| ITGB1-liver | UBE2D1-muscle | -0.70909 | 0.014552 | 0.050131 |
| KHDRBS1-liver | UBE2D1-muscle | -0.71818 | 0.0128 | 0.048396 |
| MMGT1-liver | UBE2D1-muscle | -0.74545 | 0.008455 | 0.041717 |
| NCKAP1-liver | UBE2D1-muscle | -0.76364 | 0.006233 | 0.036383 |
| RAP2A-liver | UBE2D1-muscle | -0.70909 | 0.014552 | 0.050131 |
| SLC25A6-liver | UBE2D1-muscle | -0.71818 | 0.0128 | 0.048396 |
| SUCO-liver | UBE2D1-muscle | -0.7 | 0.016471 | 0.052234 |
| TPM4-liver | UBE2D1-muscle | -0.70909 | 0.014552 | 0.050131 |
| TTF2-liver | UBE2D1-muscle | -0.78182 | 0.004473 | 0.033769 |
| YWHAZ-liver | UBE2D1-muscle | -0.70909 | 0.014552 | 0.050131 |
| ABI2-liver | UQCR11-muscle | -0.7 | 0.016471 | 0.052234 |
| ADGRL4-liver | UQCR11-muscle | -0.83636 | 0.001333 | 0.025685 |
| ANXA5-liver | UQCR11-muscle | -0.87273 | 0.000455 | 0.020203 |
| APP-liver | UQCR11-muscle | -0.95455 | 4.99E-06 | 0.016628 |
| ARHGAP18-liver | UQCR11-muscle | -0.87273 | 0.000455 | 0.020203 |
| ARID5B-liver | UQCR11-muscle | -0.81818 | 0.002083 | 0.027717 |
| ATL3-liver | UQCR11-muscle | -0.70909 | 0.014552 | 0.050131 |
| B4GALT5-liver | UQCR11-muscle | -0.78182 | 0.004473 | 0.033769 |
| BMPR2-liver | UQCR11-muscle | -0.80909 | 0.002559 | 0.029155 |
| CA5B-liver | UQCR11-muscle | -0.76364 | 0.006233 | 0.036383 |
| CAMSAP2-liver | UQCR11-muscle | -0.75455 | 0.007282 | 0.038865 |
| CAPNS1-liver | UQCR11-muscle | -0.71818 | 0.0128 | 0.048396 |
| CCN4-liver | UQCR11-muscle | -0.84545 | 0.001045 | 0.024446 |
| CD34-liver | UQCR11-muscle | -0.87273 | 0.000455 | 0.020203 |
| CDH5-liver | UQCR11-muscle | -0.85455 | 0.000807 | 0.022594 |
| CLIC1-liver | UQCR11-muscle | -0.89091 | 0.000233 | 0.018341 |
| DEFB1-liver | UQCR11-muscle | -0.84545 | 0.001045 | 0.024446 |
| DSG2-liver | UQCR11-muscle | -0.92727 | 3.97E-05 | 0.016759 |
| EPS8-liver | UQCR11-muscle | -0.82727 | 0.001677 | 0.02618 |
| EZR-liver | UQCR11-muscle | -0.83636 | 0.001333 | 0.025685 |
| FNBP1L-liver | UQCR11-muscle | -0.9 | 0.00016 | 0.016927 |
| HDAC2-liver | UQCR11-muscle | -0.76364 | 0.006233 | 0.036383 |
| HEG1-liver | UQCR11-muscle | -0.85455 | 0.000807 | 0.022594 |
| IFT57-liver | UQCR11-muscle | -0.82727 | 0.001677 | 0.02618 |
| IPO7-liver | UQCR11-muscle | -0.77273 | 0.005299 | 0.035326 |
| ITGB1-liver | UQCR11-muscle | -0.90909 | 0.000106 | 0.016759 |
| KHDRBS1-liver | UQCR11-muscle | -0.91818 | 6.66E-05 | 0.016759 |
| KIF3A-liver | UQCR11-muscle | -0.87273 | 0.000455 | 0.020203 |
| KIF5B-liver | UQCR11-muscle | -0.78182 | 0.004473 | 0.033769 |
| MFGE8-liver | UQCR11-muscle | -0.85455 | 0.000807 | 0.022594 |
| MFSD6-liver | UQCR11-muscle | -0.8 | 0.00311 | 0.031511 |
| MMGT1-liver | UQCR11-muscle | -0.9 | 0.00016 | 0.016927 |
| MTMR6-liver | UQCR11-muscle | -0.71818 | 0.0128 | 0.048396 |
| MYH9-liver | UQCR11-muscle | -0.89091 | 0.000233 | 0.018341 |
| NCKAP1-liver | UQCR11-muscle | -0.90909 | 0.000106 | 0.016759 |
| PLPP5-liver | UQCR11-muscle | -0.80909 | 0.002559 | 0.029155 |
| PRKX-liver | UQCR11-muscle | -0.9 | 0.00016 | 0.016927 |
| PRRC2C-liver | UQCR11-muscle | -0.80909 | 0.002559 | 0.029155 |
| RALB-liver | UQCR11-muscle | -0.83636 | 0.001333 | 0.025685 |
| RAP2A-liver | UQCR11-muscle | -0.88182 | 0.00033 | 0.018341 |
| RPS6KA2-liver | UQCR11-muscle | -0.81818 | 0.002083 | 0.027717 |
| SLC25A6-liver | UQCR11-muscle | -0.92727 | 3.97E-05 | 0.016759 |
| SMIM10-liver | UQCR11-muscle | -0.83636 | 0.001333 | 0.025685 |
| STX7-liver | UQCR11-muscle | -0.88182 | 0.00033 | 0.018341 |
| SUCO-liver | UQCR11-muscle | -0.9 | 0.00016 | 0.016927 |
| SULF1-liver | UQCR11-muscle | -0.89091 | 0.000233 | 0.018341 |
| TES-liver | UQCR11-muscle | -0.9 | 0.00016 | 0.016927 |
| TIMP1-liver | UQCR11-muscle | -0.87273 | 0.000455 | 0.020203 |
| TMSB10-liver | UQCR11-muscle | -0.87273 | 0.000455 | 0.020203 |
| TPM4-liver | UQCR11-muscle | -0.86364 | 0.000612 | 0.022041 |
| TSPAN14-liver | UQCR11-muscle | -0.81818 | 0.002083 | 0.027717 |
| TTF2-liver | UQCR11-muscle | -0.71818 | 0.0128 | 0.048396 |
| VCAN-liver | UQCR11-muscle | -0.83636 | 0.001333 | 0.025685 |
| WDR1-liver | UQCR11-muscle | -0.76364 | 0.006233 | 0.036383 |
| YWHAB-liver | UQCR11-muscle | -0.7 | 0.016471 | 0.052234 |
| YWHAZ-liver | UQCR11-muscle | -0.90909 | 0.000106 | 0.016759 |
| ABI2-liver | VDAC1-muscle | -0.83636 | 0.001333 | 0.025685 |
| ADGRL4-liver | VDAC1-muscle | -0.9 | 0.00016 | 0.016927 |
| ALCAM-liver | VDAC1-muscle | -0.71818 | 0.0128 | 0.048396 |
| ANXA5-liver | VDAC1-muscle | -0.90909 | 0.000106 | 0.016759 |
| AP2B1-liver | VDAC1-muscle | -0.75455 | 0.007282 | 0.038865 |
| APP-liver | VDAC1-muscle | -0.88182 | 0.00033 | 0.018341 |
| ARHGAP18-liver | VDAC1-muscle | -0.92727 | 3.97E-05 | 0.016759 |
| ARID5B-liver | VDAC1-muscle | -0.91818 | 6.66E-05 | 0.016759 |
| ATL3-liver | VDAC1-muscle | -0.73636 | 0.00976 | 0.043199 |
| B4GALT5-liver | VDAC1-muscle | -0.78182 | 0.004473 | 0.033769 |
| BMPR2-liver | VDAC1-muscle | -0.74545 | 0.008455 | 0.041717 |
| CAMSAP2-liver | VDAC1-muscle | -0.84545 | 0.001045 | 0.024446 |
| CAPNS1-liver | VDAC1-muscle | -0.75455 | 0.007282 | 0.038865 |
| CCN4-liver | VDAC1-muscle | -0.92727 | 3.97E-05 | 0.016759 |
| CD34-liver | VDAC1-muscle | -0.86364 | 0.000612 | 0.022041 |
| CDH5-liver | VDAC1-muscle | -0.90909 | 0.000106 | 0.016759 |
| CLIC1-liver | VDAC1-muscle | -0.85455 | 0.000807 | 0.022594 |
| DEFB1-liver | VDAC1-muscle | -0.86364 | 0.000612 | 0.022041 |
| DSG2-liver | VDAC1-muscle | -0.90909 | 0.000106 | 0.016759 |
| EPS8-liver | VDAC1-muscle | -0.9 | 0.00016 | 0.016927 |
| EZR-liver | VDAC1-muscle | -0.80909 | 0.002559 | 0.029155 |
| FNBP1L-liver | VDAC1-muscle | -0.9 | 0.00016 | 0.016927 |
| HDAC2-liver | VDAC1-muscle | -0.82727 | 0.001677 | 0.02618 |
| HEG1-liver | VDAC1-muscle | -0.86364 | 0.000612 | 0.022041 |
| IFT57-liver | VDAC1-muscle | -0.89091 | 0.000233 | 0.018341 |
| IPO7-liver | VDAC1-muscle | -0.7 | 0.016471 | 0.052234 |
| ITGB1-liver | VDAC1-muscle | -0.87273 | 0.000455 | 0.020203 |
| KHDRBS1-liver | VDAC1-muscle | -0.88182 | 0.00033 | 0.018341 |
| KIF3A-liver | VDAC1-muscle | -0.89091 | 0.000233 | 0.018341 |
| KIF5B-liver | VDAC1-muscle | -0.70909 | 0.014552 | 0.050131 |
| MFGE8-liver | VDAC1-muscle | -0.95455 | 4.99E-06 | 0.016628 |
| MFSD6-liver | VDAC1-muscle | -0.84545 | 0.001045 | 0.024446 |
| MMGT1-liver | VDAC1-muscle | -0.93636 | 2.21E-05 | 0.016759 |
| MTMR6-liver | VDAC1-muscle | -0.79091 | 0.003746 | 0.032221 |
| MYH9-liver | VDAC1-muscle | -0.9 | 0.00016 | 0.016927 |
| NCKAP1-liver | VDAC1-muscle | -0.84545 | 0.001045 | 0.024446 |
| PLPP5-liver | VDAC1-muscle | -0.85455 | 0.000807 | 0.022594 |
| PRKX-liver | VDAC1-muscle | -0.84545 | 0.001045 | 0.024446 |
| PRRC2C-liver | VDAC1-muscle | -0.85455 | 0.000807 | 0.022594 |
| RALB-liver | VDAC1-muscle | -0.86364 | 0.000612 | 0.022041 |
| RAP2A-liver | VDAC1-muscle | -0.86364 | 0.000612 | 0.022041 |
| RPS6KA2-liver | VDAC1-muscle | -0.87273 | 0.000455 | 0.020203 |
| SLC25A6-liver | VDAC1-muscle | -0.92727 | 3.97E-05 | 0.016759 |
| SMIM10-liver | VDAC1-muscle | -0.80909 | 0.002559 | 0.029155 |
| STX7-liver | VDAC1-muscle | -0.91818 | 6.66E-05 | 0.016759 |
| SUCO-liver | VDAC1-muscle | -0.94545 | 1.12E-05 | 0.016759 |
| SULF1-liver | VDAC1-muscle | -0.85455 | 0.000807 | 0.022594 |
| TES-liver | VDAC1-muscle | -0.90909 | 0.000106 | 0.016759 |
| TIMP1-liver | VDAC1-muscle | -0.88182 | 0.00033 | 0.018341 |
| TMSB10-liver | VDAC1-muscle | -0.85455 | 0.000807 | 0.022594 |
| TPM4-liver | VDAC1-muscle | -0.81818 | 0.002083 | 0.027717 |
| TSPAN14-liver | VDAC1-muscle | -0.83636 | 0.001333 | 0.025685 |
| TTF2-liver | VDAC1-muscle | -0.7 | 0.016471 | 0.052234 |
| VCAN-liver | VDAC1-muscle | -0.90909 | 0.000106 | 0.016759 |
| WDR1-liver | VDAC1-muscle | -0.8 | 0.00311 | 0.031511 |
| YWHAB-liver | VDAC1-muscle | -0.7 | 0.016471 | 0.052234 |
| YWHAZ-liver | VDAC1-muscle | -0.84545 | 0.001045 | 0.024446 |
| ZEB1-liver | VDAC1-muscle | -0.70909 | 0.014552 | 0.050131 |

**Supplement Table 1 represents the top 5% in Liver-1 gene centrality and in Muscle-5, with calculated Spearman correlations between the two groups, focusing only on strong correlations of <-0.7 with a p<0.05. There were 2102 pairs (65 liver genes and 83 muscle genes).**

**Supplement Table 2**

| **Liver-1** | **Muscle-35** | **Correlation** | **p_value** | **adjusted_p_value** |
| --- | --- | --- | --- | --- |
| ANXA5-liver | GEMIN7-muscle | -0.74545 | 0.008455 | 0.075779 |
| APP-liver | GEMIN7-muscle | -0.71818 | 0.0128 | 0.084477 |
| ARHGAP18-liver | GEMIN7-muscle | -0.74545 | 0.008455 | 0.075779 |
| ARID5B-liver | GEMIN7-muscle | -0.8 | 0.00311 | 0.061032 |
| CCN4-liver | GEMIN7-muscle | -0.7 | 0.016471 | 0.097219 |
| CD34-liver | GEMIN7-muscle | -0.80909 | 0.002559 | 0.061032 |
| CDH5-liver | GEMIN7-muscle | -0.76364 | 0.006233 | 0.068564 |
| CLIC1-liver | GEMIN7-muscle | -0.78182 | 0.004473 | 0.068564 |
| DSG2-liver | GEMIN7-muscle | -0.72727 | 0.011205 | 0.08217 |
| EPS8-liver | GEMIN7-muscle | -0.76364 | 0.006233 | 0.068564 |
| EZR-liver | GEMIN7-muscle | -0.8 | 0.00311 | 0.061032 |
| FNBP1L-liver | GEMIN7-muscle | -0.76364 | 0.006233 | 0.068564 |
| HEG1-liver | GEMIN7-muscle | -0.73636 | 0.00976 | 0.077015 |
| KHDRBS1-liver | GEMIN7-muscle | -0.73636 | 0.00976 | 0.077015 |
| KIF3A-liver | GEMIN7-muscle | -0.71818 | 0.0128 | 0.084477 |
| MFGE8-liver | GEMIN7-muscle | -0.76364 | 0.006233 | 0.068564 |
| MFSD6-liver | GEMIN7-muscle | -0.78182 | 0.004473 | 0.068564 |
| MMGT1-liver | GEMIN7-muscle | -0.70909 | 0.014552 | 0.091076 |
| NCKAP1-liver | GEMIN7-muscle | -0.70909 | 0.014552 | 0.091076 |
| PLPP5-liver | GEMIN7-muscle | -0.72727 | 0.011205 | 0.08217 |
| PRKX-liver | GEMIN7-muscle | -0.79091 | 0.003746 | 0.063248 |
| PRRC2C-liver | GEMIN7-muscle | -0.72727 | 0.011205 | 0.08217 |
| RAP2A-liver | GEMIN7-muscle | -0.79091 | 0.003746 | 0.063248 |
| SLC25A6-liver | GEMIN7-muscle | -0.73636 | 0.00976 | 0.077015 |
| SMIM10-liver | GEMIN7-muscle | -0.74545 | 0.008455 | 0.075779 |
| STX7-liver | GEMIN7-muscle | -0.80909 | 0.002559 | 0.061032 |
| SUCO-liver | GEMIN7-muscle | -0.75455 | 0.007282 | 0.072421 |
| SULF1-liver | GEMIN7-muscle | -0.78182 | 0.004473 | 0.068564 |
| TES-liver | GEMIN7-muscle | -0.72727 | 0.011205 | 0.08217 |
| TIMP1-liver | GEMIN7-muscle | -0.81818 | 0.002083 | 0.061032 |
| TMSB10-liver | GEMIN7-muscle | -0.72727 | 0.011205 | 0.08217 |
| VCAN-liver | GEMIN7-muscle | -0.73636 | 0.00976 | 0.077015 |
| YWHAZ-liver | GEMIN7-muscle | -0.79091 | 0.003746 | 0.063248 |
| ANKRD27-liver | ISCU-muscle | -0.72727 | 0.011205 | 0.08217 |
| TMEM138-liver | ISCU-muscle | -0.71818 | 0.0128 | 0.084477 |
| CDH5-liver | ITPA-muscle | -0.7 | 0.016471 | 0.097219 |
| EZR-liver | ITPA-muscle | -0.76364 | 0.006233 | 0.068564 |
| KHDRBS1-liver | ITPA-muscle | -0.71818 | 0.0128 | 0.084477 |
| NCKAP1-liver | ITPA-muscle | -0.71818 | 0.0128 | 0.084477 |
| PRKX-liver | ITPA-muscle | -0.7 | 0.016471 | 0.097219 |
| YWHAB-liver | ITPA-muscle | -0.74545 | 0.008455 | 0.075779 |
| ADGRL4-liver | MYL2-muscle | -0.75455 | 0.007282 | 0.072421 |
| ANKRD27-liver | MYL2-muscle | -0.77273 | 0.005299 | 0.068564 |
| ANXA5-liver | MYL2-muscle | -0.74545 | 0.008455 | 0.075779 |
| APP-liver | MYL2-muscle | -0.88182 | 0.00033 | 0.034243 |
| ARHGAP18-liver | MYL2-muscle | -0.8 | 0.00311 | 0.061032 |
| B4GALT5-liver | MYL2-muscle | -0.73636 | 0.00976 | 0.077015 |
| BMPR2-liver | MYL2-muscle | -0.80909 | 0.002559 | 0.061032 |
| CA5B-liver | MYL2-muscle | -0.77273 | 0.005299 | 0.068564 |
| CCN4-liver | MYL2-muscle | -0.76364 | 0.006233 | 0.068564 |
| CD34-liver | MYL2-muscle | -0.75455 | 0.007282 | 0.072421 |
| CDH5-liver | MYL2-muscle | -0.79091 | 0.003746 | 0.063248 |
| CLIC1-liver | MYL2-muscle | -0.8 | 0.00311 | 0.061032 |
| DEFB1-liver | MYL2-muscle | -0.77273 | 0.005299 | 0.068564 |
| DSG2-liver | MYL2-muscle | -0.88182 | 0.00033 | 0.034243 |
| EPS8-liver | MYL2-muscle | -0.80909 | 0.002559 | 0.061032 |
| EZR-liver | MYL2-muscle | -0.90909 | 0.000106 | 0.019165 |
| FNBP1L-liver | MYL2-muscle | -0.85455 | 0.000807 | 0.05324 |
| HDAC2-liver | MYL2-muscle | -0.71818 | 0.0128 | 0.084477 |
| HEG1-liver | MYL2-muscle | -0.79091 | 0.003746 | 0.063248 |
| IFT57-liver | MYL2-muscle | -0.71818 | 0.0128 | 0.084477 |
| IPO7-liver | MYL2-muscle | -0.77273 | 0.005299 | 0.068564 |
| ITGB1-liver | MYL2-muscle | -0.86364 | 0.000612 | 0.044409 |
| KHDRBS1-liver | MYL2-muscle | -0.90909 | 0.000106 | 0.019165 |
| KIF3A-liver | MYL2-muscle | -0.80909 | 0.002559 | 0.061032 |
| KIF5B-liver | MYL2-muscle | -0.86364 | 0.000612 | 0.044409 |
| MFGE8-liver | MYL2-muscle | -0.71818 | 0.0128 | 0.084477 |
| MFSD6-liver | MYL2-muscle | -0.81818 | 0.002083 | 0.061032 |
| MMGT1-liver | MYL2-muscle | -0.82727 | 0.001677 | 0.061032 |
| MTMR6-liver | MYL2-muscle | -0.75455 | 0.007282 | 0.072421 |
| MYH9-liver | MYL2-muscle | -0.77273 | 0.005299 | 0.068564 |
| NCKAP1-liver | MYL2-muscle | -0.95455 | 4.99E-06 | 0.003622 |
| PLPP5-liver | MYL2-muscle | -0.82727 | 0.001677 | 0.061032 |
| PRKX-liver | MYL2-muscle | -0.90909 | 0.000106 | 0.019165 |
| PRRC2C-liver | MYL2-muscle | -0.80909 | 0.002559 | 0.061032 |
| RALB-liver | MYL2-muscle | -0.75455 | 0.007282 | 0.072421 |
| RAP2A-liver | MYL2-muscle | -0.8 | 0.00311 | 0.061032 |
| SLC25A6-liver | MYL2-muscle | -0.89091 | 0.000233 | 0.033898 |
| SMIM10-liver | MYL2-muscle | -0.77273 | 0.005299 | 0.068564 |
| STX7-liver | MYL2-muscle | -0.73636 | 0.00976 | 0.077015 |
| SUCO-liver | MYL2-muscle | -0.80909 | 0.002559 | 0.061032 |
| SULF1-liver | MYL2-muscle | -0.8 | 0.00311 | 0.061032 |
| TES-liver | MYL2-muscle | -0.81818 | 0.002083 | 0.061032 |
| TIMP1-liver | MYL2-muscle | -0.76364 | 0.006233 | 0.068564 |
| TMSB10-liver | MYL2-muscle | -0.80909 | 0.002559 | 0.061032 |
| TPM4-liver | MYL2-muscle | -0.83636 | 0.001333 | 0.061032 |
| TSPAN14-liver | MYL2-muscle | -0.78182 | 0.004473 | 0.068564 |
| TTF2-liver | MYL2-muscle | -0.70909 | 0.014552 | 0.091076 |
| VCAN-liver | MYL2-muscle | -0.77273 | 0.005299 | 0.068564 |
| VEZF1-liver | MYL2-muscle | -0.81818 | 0.002083 | 0.061032 |
| WDR1-liver | MYL2-muscle | -0.76364 | 0.006233 | 0.068564 |
| YWHAB-liver | MYL2-muscle | -0.73636 | 0.00976 | 0.077015 |
| YWHAZ-liver | MYL2-muscle | -0.80909 | 0.002559 | 0.061032 |
| IPO7-liver | RNF212B-muscle | -0.71818 | 0.0128 | 0.084477 |
| NCKAP1-liver | RNF212B-muscle | -0.7 | 0.016471 | 0.097219 |
| TTF2-liver | RNF212B-muscle | -0.81818 | 0.002083 | 0.061032 |
| ANKRD27-liver | SEC14L5-muscle | -0.7 | 0.016471 | 0.097219 |
| ARHGAP18-liver | SEC14L5-muscle | -0.74545 | 0.008455 | 0.075779 |
| BMPR2-liver | SEC14L5-muscle | -0.73636 | 0.00976 | 0.077015 |
| CA5B-liver | SEC14L5-muscle | -0.71818 | 0.0128 | 0.084477 |
| CCN4-liver | SEC14L5-muscle | -0.70909 | 0.014552 | 0.091076 |
| CDH5-liver | SEC14L5-muscle | -0.77273 | 0.005299 | 0.068564 |
| DSG2-liver | SEC14L5-muscle | -0.75455 | 0.007282 | 0.072421 |
| EPS8-liver | SEC14L5-muscle | -0.80909 | 0.002559 | 0.061032 |
| EZR-liver | SEC14L5-muscle | -0.8 | 0.00311 | 0.061032 |
| FNBP1L-liver | SEC14L5-muscle | -0.76364 | 0.006233 | 0.068564 |
| HEG1-liver | SEC14L5-muscle | -0.7 | 0.016471 | 0.097219 |
| IPO7-liver | SEC14L5-muscle | -0.77273 | 0.005299 | 0.068564 |
| ITGB1-liver | SEC14L5-muscle | -0.73636 | 0.00976 | 0.077015 |
| KHDRBS1-liver | SEC14L5-muscle | -0.70909 | 0.014552 | 0.091076 |
| KIF5B-liver | SEC14L5-muscle | -0.7 | 0.016471 | 0.097219 |
| MFSD6-liver | SEC14L5-muscle | -0.72727 | 0.011205 | 0.08217 |
| MMGT1-liver | SEC14L5-muscle | -0.73636 | 0.00976 | 0.077015 |
| NCKAP1-liver | SEC14L5-muscle | -0.79091 | 0.003746 | 0.063248 |
| PLPP5-liver | SEC14L5-muscle | -0.86364 | 0.000612 | 0.044409 |
| PRKX-liver | SEC14L5-muscle | -0.74545 | 0.008455 | 0.075779 |
| PRRC2C-liver | SEC14L5-muscle | -0.80909 | 0.002559 | 0.061032 |
| RAP2A-liver | SEC14L5-muscle | -0.70909 | 0.014552 | 0.091076 |
| SLC25A6-liver | SEC14L5-muscle | -0.74545 | 0.008455 | 0.075779 |
| SUCO-liver | SEC14L5-muscle | -0.75455 | 0.007282 | 0.072421 |
| TMEM138-liver | SEC14L5-muscle | -0.73636 | 0.00976 | 0.077015 |
| TTF2-liver | SEC14L5-muscle | -0.76364 | 0.006233 | 0.068564 |
| VCAN-liver | SEC14L5-muscle | -0.71818 | 0.0128 | 0.084477 |

**Supplement Table 2 represents the top 5% in Liver-1 gene centrality and in Muscle-35, with calculated Spearman correlations between the two groups, focusing only on strong correlations of <-0.7 with a p<0.05.**

**Supplement Table 3**

| **Liver-2** | **Muscle-35** | **Correlation** | **p_value** | **adjusted_p_value** |
| --- | --- | --- | --- | --- |
| ARCN1-liver | ITPA-muscle | -0.7 | 0.016471 | 0.32938 |
| HNRNPUL1-liver | ITPA-muscle | -0.72727 | 0.011205 | 0.308137 |
| KDM7A-liver | ITPA-muscle | -0.71818 | 0.0128 | 0.31999 |
| POMP-liver | ITPA-muscle | -0.70909 | 0.014552 | 0.32938 |
| RAB22A-liver | ITPA-muscle | -0.74545 | 0.008455 | 0.308137 |
| RABGGTB-liver | ITPA-muscle | -0.82727 | 0.001677 | 0.307445 |
| RIOK1-liver | ITPA-muscle | -0.84545 | 0.001045 | 0.287425 |
| RRAGA-liver | ITPA-muscle | -0.73636 | 0.00976 | 0.308137 |
| RRP36-liver | ITPA-muscle | -0.7 | 0.016471 | 0.32938 |
| SERINC1-liver | ITPA-muscle | -0.72727 | 0.011205 | 0.308137 |
| TIPRL-liver | ITPA-muscle | -0.74545 | 0.008455 | 0.308137 |
| DDX1-liver | KIR2DS4-muscle | -0.76364 | 0.006233 | 0.308137 |
| LAMTOR2-liver | KIR2DS4-muscle | -0.87273 | 0.000455 | 0.250038 |
| MRPS10-liver | KIR2DS4-muscle | -0.73636 | 0.00976 | 0.308137 |
| MRPS15-liver | KIR2DS4-muscle | -0.76364 | 0.006233 | 0.308137 |
| MYNN-liver | KIR2DS4-muscle | -0.72727 | 0.011205 | 0.308137 |
| PSMB6-liver | KIR2DS4-muscle | -0.73636 | 0.00976 | 0.308137 |
| TXNL4A-liver | KIR2DS4-muscle | -0.73636 | 0.00976 | 0.308137 |
| UBE2V2-liver | KIR2DS4-muscle | -0.75627 | 0.007074 | 0.308137 |
| YES1-liver | KIR2DS4-muscle | -0.73636 | 0.00976 | 0.308137 |
| EIF3M-liver | MEN1-muscle | -0.73636 | 0.00976 | 0.308137 |
| HNRNPUL1-liver | MEN1-muscle | -0.76364 | 0.006233 | 0.308137 |
| KDM7A-liver | MEN1-muscle | -0.71818 | 0.0128 | 0.31999 |
| LAMTOR2-liver | MEN1-muscle | -0.78182 | 0.004473 | 0.308137 |
| RSL1D1-liver | MEN1-muscle | -0.7 | 0.016471 | 0.32938 |
| UBE2V2-liver | MEN1-muscle | -0.72893 | 0.01093 | 0.308137 |

**Supplement Table 3 represents the top 5% in Liver-2 gene centrality and in Muscle-35, with calculated Spearman correlations between the two groups, focusing only on strong correlations of <-0.7 with a p<0.05. There were 2102 pairs (65 liver genes and 83 muscle genes).**

**Supplement Table-4**

| **Liver-1** | **Fat-1** | **Correlation** | **p_value** | **adjusted_p_value** |
| --- | --- | --- | --- | --- |
| ABI2-liver | BRIX1-fat | 0.736364 | 0.00976 | 0.377593 |
| ANXA5-liver | BRIX1-fat | 0.763636 | 0.006233 | 0.377593 |
| ARHGAP18-liver | BRIX1-fat | 0.727273 | 0.011205 | 0.388748 |
| ARID5B-liver | BRIX1-fat | 0.709091 | 0.014552 | 0.388748 |
| CAMSAP2-liver | BRIX1-fat | 0.790909 | 0.003746 | 0.377593 |
| CCN4-liver | BRIX1-fat | 0.754545 | 0.007282 | 0.377593 |
| CD34-liver | BRIX1-fat | 0.754545 | 0.007282 | 0.377593 |
| CDH5-liver | BRIX1-fat | 0.845455 | 0.001045 | 0.377593 |
| CLIC1-liver | BRIX1-fat | 0.709091 | 0.014552 | 0.388748 |
| EPS8-liver | BRIX1-fat | 0.745455 | 0.008455 | 0.377593 |
| FNBP1L-liver | BRIX1-fat | 0.727273 | 0.011205 | 0.388748 |
| KIF3A-liver | BRIX1-fat | 0.7 | 0.016471 | 0.390432 |
| MFGE8-liver | BRIX1-fat | 0.736364 | 0.00976 | 0.377593 |
| MFSD6-liver | BRIX1-fat | 0.754545 | 0.007282 | 0.377593 |
| PRKX-liver | BRIX1-fat | 0.709091 | 0.014552 | 0.388748 |
| PRRC2C-liver | BRIX1-fat | 0.772727 | 0.005299 | 0.377593 |
| RALB-liver | BRIX1-fat | 0.772727 | 0.005299 | 0.377593 |
| RAP2A-liver | BRIX1-fat | 0.709091 | 0.014552 | 0.388748 |
| RPS6KA2-liver | BRIX1-fat | 0.818182 | 0.002083 | 0.377593 |
| SLC25A6-liver | BRIX1-fat | 0.709091 | 0.014552 | 0.388748 |
| STX7-liver | BRIX1-fat | 0.827273 | 0.001677 | 0.377593 |
| SUCO-liver | BRIX1-fat | 0.772727 | 0.005299 | 0.377593 |
| SULF1-liver | BRIX1-fat | 0.709091 | 0.014552 | 0.388748 |
| TES-liver | BRIX1-fat | 0.745455 | 0.008455 | 0.377593 |
| TIMP1-liver | BRIX1-fat | 0.8 | 0.00311 | 0.377593 |
| WDR1-liver | BRIX1-fat | 0.718182 | 0.0128 | 0.388748 |
| ZEB1-liver | BRIX1-fat | 0.790909 | 0.003746 | 0.377593 |
| BCL2L11-liver | CASK-fat | 0.8 | 0.00311 | 0.377593 |
| ABI2-liver | CLEC2B-fat | 0.709091 | 0.014552 | 0.388748 |
| CAMSAP2-liver | CLEC2B-fat | 0.7 | 0.016471 | 0.390432 |
| ZEB1-liver | CLEC2B-fat | 0.772727 | 0.005299 | 0.377593 |
| AGPAT5-liver | CSNK2A2-fat | 0.7 | 0.016471 | 0.390432 |
| ATL3-liver | CSNK2A2-fat | 0.709091 | 0.014552 | 0.388748 |
| B4GALT5-liver | CSNK2A2-fat | 0.754545 | 0.007282 | 0.377593 |
| HDAC2-liver | CSNK2A2-fat | 0.7 | 0.016471 | 0.390432 |
| MKKS-liver | CSNK2A2-fat | 0.754545 | 0.007282 | 0.377593 |
| YWHAQ-liver | CSNK2A2-fat | 0.763636 | 0.006233 | 0.377593 |
| ARID5B-liver | EIF2B2-fat | 0.779045 | 0.004714 | 0.377593 |
| CD34-liver | EIF2B2-fat | 0.71982 | 0.012501 | 0.388748 |
| CDH5-liver | EIF2B2-fat | 0.710708 | 0.014228 | 0.388748 |
| EPS8-liver | EIF2B2-fat | 0.733487 | 0.010201 | 0.388748 |
| MFGE8-liver | EIF2B2-fat | 0.747155 | 0.008226 | 0.377593 |
| MFSD6-liver | EIF2B2-fat | 0.728931 | 0.01093 | 0.388748 |
| STX7-liver | EIF2B2-fat | 0.760822 | 0.006545 | 0.377593 |
| TIMP1-liver | EIF2B2-fat | 0.75171 | 0.007634 | 0.377593 |
| ZEB1-liver | EIF2B2-fat | 0.75171 | 0.007634 | 0.377593 |
| ATL3-liver | ERAL1-fat | 0.709091 | 0.014552 | 0.388748 |
| B4GALT5-liver | ERAL1-fat | 0.736364 | 0.00976 | 0.377593 |
| CAPNS1-liver | ERAL1-fat | 0.7 | 0.016471 | 0.390432 |
| MKKS-liver | ERAL1-fat | 0.736364 | 0.00976 | 0.377593 |
| ZEB1-liver | ERAL1-fat | 0.763636 | 0.006233 | 0.377593 |
| ALCAM-liver | MFSD9-fat | 0.781818 | 0.004473 | 0.377593 |
| ARHGAP18-liver | MFSD9-fat | 0.7 | 0.016471 | 0.390432 |
| BCL2L11-liver | MFSD9-fat | 0.7 | 0.016471 | 0.390432 |
| CDH5-liver | MFSD9-fat | 0.736364 | 0.00976 | 0.377593 |
| MMGT1-liver | MFSD9-fat | 0.718182 | 0.0128 | 0.388748 |
| ABI2-liver | NUP50-fat | 0.790909 | 0.003746 | 0.377593 |
| ADGRL4-liver | NUP50-fat | 0.718182 | 0.0128 | 0.388748 |
| AGPAT5-liver | NUP50-fat | 0.754545 | 0.007282 | 0.377593 |
| ANKRD27-liver | NUP50-fat | 0.827273 | 0.001677 | 0.377593 |
| AP2B1-liver | NUP50-fat | 0.745455 | 0.008455 | 0.377593 |
| APP-liver | NUP50-fat | 0.727273 | 0.011205 | 0.388748 |
| ARHGAP18-liver | NUP50-fat | 0.754545 | 0.007282 | 0.377593 |
| ATL3-liver | NUP50-fat | 0.845455 | 0.001045 | 0.377593 |
| B4GALT5-liver | NUP50-fat | 0.836364 | 0.001333 | 0.377593 |
| BMPR2-liver | NUP50-fat | 0.818182 | 0.002083 | 0.377593 |
| CA5B-liver | NUP50-fat | 0.8 | 0.00311 | 0.377593 |
| CAPNS1-liver | NUP50-fat | 0.827273 | 0.001677 | 0.377593 |
| CD34-liver | NUP50-fat | 0.709091 | 0.014552 | 0.388748 |
| CLIC1-liver | NUP50-fat | 0.736364 | 0.00976 | 0.377593 |
| DEFB1-liver | NUP50-fat | 0.7 | 0.016471 | 0.390432 |
| DSG2-liver | NUP50-fat | 0.745455 | 0.008455 | 0.377593 |
| FNBP1L-liver | NUP50-fat | 0.763636 | 0.006233 | 0.377593 |
| HDAC2-liver | NUP50-fat | 0.827273 | 0.001677 | 0.377593 |
| HEG1-liver | NUP50-fat | 0.754545 | 0.007282 | 0.377593 |
| ITGB1-liver | NUP50-fat | 0.763636 | 0.006233 | 0.377593 |
| KIF3A-liver | NUP50-fat | 0.763636 | 0.006233 | 0.377593 |
| KIF5B-liver | NUP50-fat | 0.872727 | 0.000455 | 0.377593 |
| MMGT1-liver | NUP50-fat | 0.736364 | 0.00976 | 0.377593 |
| RAP2A-liver | NUP50-fat | 0.790909 | 0.003746 | 0.377593 |
| RPS6KA2-liver | NUP50-fat | 0.7 | 0.016471 | 0.390432 |
| SMIM10-liver | NUP50-fat | 0.854545 | 0.000807 | 0.377593 |
| SUCO-liver | NUP50-fat | 0.709091 | 0.014552 | 0.388748 |
| SULF1-liver | NUP50-fat | 0.736364 | 0.00976 | 0.377593 |
| TES-liver | NUP50-fat | 0.718182 | 0.0128 | 0.388748 |
| TMSB10-liver | NUP50-fat | 0.772727 | 0.005299 | 0.377593 |
| TPM4-liver | NUP50-fat | 0.845455 | 0.001045 | 0.377593 |
| TSPAN14-liver | NUP50-fat | 0.736364 | 0.00976 | 0.377593 |
| VCAN-liver | NUP50-fat | 0.727273 | 0.011205 | 0.388748 |
| VEZF1-liver | NUP50-fat | 0.827273 | 0.001677 | 0.377593 |
| YWHAZ-liver | NUP50-fat | 0.727273 | 0.011205 | 0.388748 |
| ADGRL4-liver | PDE9A-fat | 0.7 | 0.016471 | 0.390432 |
| AGPAT5-liver | PDE9A-fat | 0.709091 | 0.014552 | 0.388748 |
| B4GALT5-liver | PDE9A-fat | 0.7 | 0.016471 | 0.390432 |
| IPO7-liver | PDE9A-fat | 0.809091 | 0.002559 | 0.377593 |
| MKKS-liver | PDE9A-fat | 0.845455 | 0.001045 | 0.377593 |
| YWHAB-liver | PDE9A-fat | 0.836364 | 0.001333 | 0.377593 |
| YWHAQ-liver | PDE9A-fat | 0.790909 | 0.003746 | 0.377593 |
| ABI2-liver | RPL30-fat | 0.727273 | 0.011205 | 0.388748 |
| ADGRL4-liver | RPL30-fat | 0.718182 | 0.0128 | 0.388748 |
| ALCAM-liver | RPL30-fat | 0.727273 | 0.011205 | 0.388748 |
| ANKRD27-liver | RPL30-fat | 0.781818 | 0.004473 | 0.377593 |
| AP2B1-liver | RPL30-fat | 0.8 | 0.00311 | 0.377593 |
| ARHGAP18-liver | RPL30-fat | 0.718182 | 0.0128 | 0.388748 |
| ATL3-liver | RPL30-fat | 0.754545 | 0.007282 | 0.377593 |
| B4GALT5-liver | RPL30-fat | 0.736364 | 0.00976 | 0.377593 |
| BMPR2-liver | RPL30-fat | 0.745455 | 0.008455 | 0.377593 |
| CAPNS1-liver | RPL30-fat | 0.745455 | 0.008455 | 0.377593 |
| HDAC2-liver | RPL30-fat | 0.818182 | 0.002083 | 0.377593 |
| IFT57-liver | RPL30-fat | 0.727273 | 0.011205 | 0.388748 |
| MKKS-liver | RPL30-fat | 0.736364 | 0.00976 | 0.377593 |
| MMGT1-liver | RPL30-fat | 0.736364 | 0.00976 | 0.377593 |
| MTMR6-liver | RPL30-fat | 0.7 | 0.016471 | 0.390432 |
| TMEM138-liver | RPL30-fat | 0.772727 | 0.005299 | 0.377593 |
| TSPAN14-liver | RPL30-fat | 0.7 | 0.016471 | 0.390432 |
| VCAN-liver | RPL30-fat | 0.7 | 0.016471 | 0.390432 |
| VEZF1-liver | RPL30-fat | 0.718182 | 0.0128 | 0.388748 |
| YWHAQ-liver | RPL30-fat | 0.772727 | 0.005299 | 0.377593 |
| ABI2-liver | RPP25L-fat | 0.854545 | 0.000807 | 0.377593 |
| ADGRL4-liver | RPP25L-fat | 0.736364 | 0.00976 | 0.377593 |
| AP2B1-liver | RPP25L-fat | 0.809091 | 0.002559 | 0.377593 |
| ARHGAP18-liver | RPP25L-fat | 0.763636 | 0.006233 | 0.377593 |
| ARID5B-liver | RPP25L-fat | 0.745455 | 0.008455 | 0.377593 |
| ATL3-liver | RPP25L-fat | 0.790909 | 0.003746 | 0.377593 |
| B4GALT5-liver | RPP25L-fat | 0.7 | 0.016471 | 0.390432 |
| CAMSAP2-liver | RPP25L-fat | 0.736364 | 0.00976 | 0.377593 |
| CAPNS1-liver | RPP25L-fat | 0.8 | 0.00311 | 0.377593 |
| CCN4-liver | RPP25L-fat | 0.718182 | 0.0128 | 0.388748 |
| DEFB1-liver | RPP25L-fat | 0.7 | 0.016471 | 0.390432 |
| HDAC2-liver | RPP25L-fat | 0.818182 | 0.002083 | 0.377593 |
| HEG1-liver | RPP25L-fat | 0.718182 | 0.0128 | 0.388748 |
| IFT57-liver | RPP25L-fat | 0.790909 | 0.003746 | 0.377593 |
| KIF3A-liver | RPP25L-fat | 0.718182 | 0.0128 | 0.388748 |
| MFGE8-liver | RPP25L-fat | 0.718182 | 0.0128 | 0.388748 |
| MMGT1-liver | RPP25L-fat | 0.745455 | 0.008455 | 0.377593 |
| MTMR6-liver | RPP25L-fat | 0.709091 | 0.014552 | 0.388748 |
| SMIM10-liver | RPP25L-fat | 0.709091 | 0.014552 | 0.388748 |
| SUCO-liver | RPP25L-fat | 0.709091 | 0.014552 | 0.388748 |
| TSPAN14-liver | RPP25L-fat | 0.754545 | 0.007282 | 0.377593 |
| VCAN-liver | RPP25L-fat | 0.772727 | 0.005299 | 0.377593 |
| YWHAQ-liver | TMEM126A-fat | 0.709091 | 0.014552 | 0.388748 |
| CDH5-liver | ZNF280C-fat | 0.754545 | 0.007282 | 0.377593 |

**Supplement Table 4 represents the top 5% in Liver-1 gene centrality and in Fat-1, with calculated Spearman correlations between the two groups, focusing only on strong correlations of >0.7 with a p<0.05.**

**Supplement Table-5**

| **Liver-2** | **Fat-1** | **Correlation** | **p_value** | **adjusted_p_value** |
| --- | --- | --- | --- | --- |
| ARCN1-liver | AKT1-fat | 0.681818 | 0.020843 | 0.222382 |
| CHCHD2-liver | AKT1-fat | 0.681818 | 0.020843 | 0.222382 |
| COX8A-liver | AKT1-fat | 0.736364 | 0.00976 | 0.211941 |
| DDX1-liver | AKT1-fat | 0.681818 | 0.020843 | 0.222382 |
| DERL1-liver | AKT1-fat | 0.690909 | 0.018565 | 0.218161 |
| EGLN1-liver | AKT1-fat | 0.681818 | 0.020843 | 0.222382 |
| EIF3M-liver | AKT1-fat | 0.790909 | 0.003746 | 0.173682 |
| HNRNPUL1-liver | AKT1-fat | 0.718182 | 0.0128 | 0.211941 |
| KDM7A-liver | AKT1-fat | 0.709091 | 0.014552 | 0.213203 |
| MYNN-liver | AKT1-fat | 0.709091 | 0.014552 | 0.213203 |
| NDUFA1-liver | AKT1-fat | 0.618182 | 0.042646 | 0.276708 |
| PIGK-liver | AKT1-fat | 0.763636 | 0.006233 | 0.198679 |
| PSMB1-liver | AKT1-fat | 0.663636 | 0.025984 | 0.237489 |
| PSMC1-liver | AKT1-fat | 0.718182 | 0.0128 | 0.211941 |
| RAB22A-liver | AKT1-fat | 0.645455 | 0.031963 | 0.251559 |
| RAB7A-liver | AKT1-fat | 0.654545 | 0.028865 | 0.250356 |
| RRAGA-liver | AKT1-fat | 0.618182 | 0.042646 | 0.276708 |
| RRP36-liver | AKT1-fat | 0.736364 | 0.00976 | 0.211941 |
| RSL1D1-liver | AKT1-fat | 0.681818 | 0.020843 | 0.222382 |
| SELENOT-liver | AKT1-fat | 0.609091 | 0.046696 | 0.28351 |
| SERINC1-liver | AKT1-fat | 0.672727 | 0.023313 | 0.231316 |
| SLC35F5-liver | AKT1-fat | 0.727273 | 0.011205 | 0.211941 |
| TIMM9-liver | AKT1-fat | 0.709091 | 0.014552 | 0.213203 |
| TMTC3-liver | AKT1-fat | 0.672727 | 0.023313 | 0.231316 |
| UBE2V2-liver | AKT1-fat | 0.701596 | 0.016122 | 0.213203 |
| UBL5-liver | AKT1-fat | 0.627273 | 0.038845 | 0.267717 |
| YES1-liver | AKT1-fat | 0.636364 | 0.035287 | 0.257828 |
| FBXL5-liver | ANAPC16-fat | 0.609091 | 0.046696 | 0.28351 |
| SLC35F5-liver | ANAPC16-fat | 0.618182 | 0.042646 | 0.276708 |
| CHCHD2-liver | APC-fat | 0.709091 | 0.014552 | 0.213203 |
| CMAS-liver | APC-fat | 0.645455 | 0.031963 | 0.251559 |
| COX8A-liver | APC-fat | 0.772727 | 0.005299 | 0.195851 |
| EIF3M-liver | APC-fat | 0.718182 | 0.0128 | 0.211941 |
| FBXL5-liver | APC-fat | 0.663636 | 0.025984 | 0.237489 |
| LAMTOR2-liver | APC-fat | 0.690909 | 0.018565 | 0.218161 |
| MRPS10-liver | APC-fat | 0.727273 | 0.011205 | 0.211941 |
| NDUFB6-liver | APC-fat | 0.627273 | 0.038845 | 0.267717 |
| PGGT1B-liver | APC-fat | 0.727273 | 0.011205 | 0.211941 |
| PIGY-liver | APC-fat | 0.645455 | 0.031963 | 0.251559 |
| PSMB1-liver | APC-fat | 0.636364 | 0.035287 | 0.257828 |
| PSMB6-liver | APC-fat | 0.727273 | 0.011205 | 0.211941 |
| SLC35F5-liver | APC-fat | 0.790909 | 0.003746 | 0.173682 |
| TXNL4A-liver | APC-fat | 0.727273 | 0.011205 | 0.211941 |
| UBE2V2-liver | APC-fat | 0.605924 | 0.048167 | 0.289018 |
| UBL5-liver | APC-fat | 0.618182 | 0.042646 | 0.276708 |
| YES1-liver | APC-fat | 0.609091 | 0.046696 | 0.28351 |
| RRAGA-liver | ARL8B-fat | 0.681818 | 0.020843 | 0.222382 |
| PIGY-liver | ATG5-fat | 0.718182 | 0.0128 | 0.211941 |
| CHCHD2-liver | ATP5MK-fat | 0.790909 | 0.003746 | 0.173682 |
| CMAS-liver | ATP5MK-fat | 0.763636 | 0.006233 | 0.198679 |
| COPS3-liver | ATP5MK-fat | 0.609091 | 0.046696 | 0.28351 |
| COX8A-liver | ATP5MK-fat | 0.836364 | 0.001333 | 0.165242 |
| DERL1-liver | ATP5MK-fat | 0.681818 | 0.020843 | 0.222382 |
| EGLN1-liver | ATP5MK-fat | 0.645455 | 0.031963 | 0.251559 |
| EIF3M-liver | ATP5MK-fat | 0.727273 | 0.011205 | 0.211941 |
| FBXL5-liver | ATP5MK-fat | 0.745455 | 0.008455 | 0.211941 |
| LAMTOR2-liver | ATP5MK-fat | 0.736364 | 0.00976 | 0.211941 |
| MRPS10-liver | ATP5MK-fat | 0.809091 | 0.002559 | 0.165242 |
| MYNN-liver | ATP5MK-fat | 0.627273 | 0.038845 | 0.267717 |
| NDUFA1-liver | ATP5MK-fat | 0.654545 | 0.028865 | 0.250356 |
| NDUFB6-liver | ATP5MK-fat | 0.772727 | 0.005299 | 0.195851 |
| PGGT1B-liver | ATP5MK-fat | 0.809091 | 0.002559 | 0.165242 |
| PIGK-liver | ATP5MK-fat | 0.645455 | 0.031963 | 0.251559 |
| PIGY-liver | ATP5MK-fat | 0.790909 | 0.003746 | 0.173682 |
| PSMB1-liver | ATP5MK-fat | 0.754545 | 0.007282 | 0.211941 |
| PSMB6-liver | ATP5MK-fat | 0.809091 | 0.002559 | 0.165242 |
| PSMC1-liver | ATP5MK-fat | 0.7 | 0.016471 | 0.213203 |
| SELENOT-liver | ATP5MK-fat | 0.672727 | 0.023313 | 0.231316 |
| SLC35F5-liver | ATP5MK-fat | 0.809091 | 0.002559 | 0.165242 |
| SMIM26-liver | ATP5MK-fat | 0.736364 | 0.00976 | 0.211941 |
| TMTC3-liver | ATP5MK-fat | 0.636364 | 0.035287 | 0.257828 |
| TXNL1-liver | ATP5MK-fat | 0.709091 | 0.014552 | 0.213203 |
| TXNL4A-liver | ATP5MK-fat | 0.781818 | 0.004473 | 0.195851 |
| UBE2V2-liver | ATP5MK-fat | 0.692485 | 0.018189 | 0.218161 |
| UBL5-liver | ATP5MK-fat | 0.727273 | 0.011205 | 0.211941 |
| VTI1B-liver | ATP5MK-fat | 0.736364 | 0.00976 | 0.211941 |
| YES1-liver | ATP5MK-fat | 0.709091 | 0.014552 | 0.213203 |
| AATF-liver | BBS2-fat | 0.672727 | 0.023313 | 0.231316 |
| ARCN1-liver | BBS2-fat | 0.727273 | 0.011205 | 0.211941 |
| CHCHD2-liver | BBS2-fat | 0.636364 | 0.035287 | 0.257828 |
| COX8A-liver | BBS2-fat | 0.654545 | 0.028865 | 0.250356 |
| DDX1-liver | BBS2-fat | 0.809091 | 0.002559 | 0.165242 |
| DERL1-liver | BBS2-fat | 0.772727 | 0.005299 | 0.195851 |
| EGLN1-liver | BBS2-fat | 0.609091 | 0.046696 | 0.28351 |
| HNRNPUL1-liver | BBS2-fat | 0.709091 | 0.014552 | 0.213203 |
| KDM7A-liver | BBS2-fat | 0.663636 | 0.025984 | 0.237489 |
| LAMTOR2-liver | BBS2-fat | 0.636364 | 0.035287 | 0.257828 |
| MRPS10-liver | BBS2-fat | 0.618182 | 0.042646 | 0.276708 |
| MRPS15-liver | BBS2-fat | 0.609091 | 0.046696 | 0.28351 |
| MYNN-liver | BBS2-fat | 0.809091 | 0.002559 | 0.165242 |
| NDUFA1-liver | BBS2-fat | 0.727273 | 0.011205 | 0.211941 |
| PIGK-liver | BBS2-fat | 0.709091 | 0.014552 | 0.213203 |
| POMP-liver | BBS2-fat | 0.681818 | 0.020843 | 0.222382 |
| PSMB1-liver | BBS2-fat | 0.727273 | 0.011205 | 0.211941 |
| PSMB6-liver | BBS2-fat | 0.618182 | 0.042646 | 0.276708 |
| PSMC1-liver | BBS2-fat | 0.645455 | 0.031963 | 0.251559 |
| RAB22A-liver | BBS2-fat | 0.745455 | 0.008455 | 0.211941 |
| RAB7A-liver | BBS2-fat | 0.763636 | 0.006233 | 0.198679 |
| RABGGTB-liver | BBS2-fat | 0.718182 | 0.0128 | 0.211941 |
| RRAGA-liver | BBS2-fat | 0.627273 | 0.038845 | 0.267717 |
| RRP36-liver | BBS2-fat | 0.736364 | 0.00976 | 0.211941 |
| RSL1D1-liver | BBS2-fat | 0.609091 | 0.046696 | 0.28351 |
| SELENOT-liver | BBS2-fat | 0.663636 | 0.025984 | 0.237489 |
| SERINC1-liver | BBS2-fat | 0.745455 | 0.008455 | 0.211941 |
| SMIM26-liver | BBS2-fat | 0.627273 | 0.038845 | 0.267717 |
| TAF13-liver | BBS2-fat | 0.763636 | 0.006233 | 0.198679 |
| TIMM9-liver | BBS2-fat | 0.763636 | 0.006233 | 0.198679 |
| TIPRL-liver | BBS2-fat | 0.645455 | 0.031963 | 0.251559 |
| TMTC3-liver | BBS2-fat | 0.736364 | 0.00976 | 0.211941 |
| TXNL1-liver | BBS2-fat | 0.645455 | 0.031963 | 0.251559 |
| UBE2V2-liver | BBS2-fat | 0.760822 | 0.006545 | 0.206049 |
| UBL5-liver | BBS2-fat | 0.663636 | 0.025984 | 0.237489 |
| UFSP2-liver | BBS2-fat | 0.727273 | 0.011205 | 0.211941 |
| YES1-liver | BBS2-fat | 0.718182 | 0.0128 | 0.211941 |
| COPS3-liver | BLMH-fat | 0.618182 | 0.042646 | 0.276708 |
| NRAS-liver | BLMH-fat | 0.772727 | 0.005299 | 0.195851 |
| RAB22A-liver | BLMH-fat | 0.645455 | 0.031963 | 0.251559 |
| RRAGA-liver | BLMH-fat | 0.663636 | 0.025984 | 0.237489 |
| RRP36-liver | BLMH-fat | 0.645455 | 0.031963 | 0.251559 |
| UFSP2-liver | BLMH-fat | 0.609091 | 0.046696 | 0.28351 |
| EGLN1-liver | BRCC3-fat | 0.636364 | 0.035287 | 0.257828 |
| EIF3M-liver | BRCC3-fat | 0.663636 | 0.025984 | 0.237489 |
| PIGY-liver | BRCC3-fat | 0.809091 | 0.002559 | 0.165242 |
| PSMC1-liver | BRCC3-fat | 0.609091 | 0.046696 | 0.28351 |
| RRAGA-liver | BRIX1-fat | 0.663636 | 0.025984 | 0.237489 |
| CHCHD2-liver | CEBPZOS-fat | 0.645455 | 0.031963 | 0.251559 |
| COPS3-liver | CEBPZOS-fat | 0.736364 | 0.00976 | 0.211941 |
| COX8A-liver | CEBPZOS-fat | 0.672727 | 0.023313 | 0.231316 |
| DERL1-liver | CEBPZOS-fat | 0.627273 | 0.038845 | 0.267717 |
| EGLN1-liver | CEBPZOS-fat | 0.690909 | 0.018565 | 0.218161 |
| MRPS10-liver | CEBPZOS-fat | 0.627273 | 0.038845 | 0.267717 |
| NDUFB6-liver | CEBPZOS-fat | 0.690909 | 0.018565 | 0.218161 |
| PGGT1B-liver | CEBPZOS-fat | 0.645455 | 0.031963 | 0.251559 |
| PIGY-liver | CEBPZOS-fat | 0.927273 | 3.97E-05 | 0.050666 |
| PSMB1-liver | CEBPZOS-fat | 0.7 | 0.016471 | 0.213203 |
| PSMB6-liver | CEBPZOS-fat | 0.627273 | 0.038845 | 0.267717 |
| PSMC1-liver | CEBPZOS-fat | 0.663636 | 0.025984 | 0.237489 |
| RAB7A-liver | CEBPZOS-fat | 0.618182 | 0.042646 | 0.276708 |
| RIOK1-liver | CEBPZOS-fat | 0.618182 | 0.042646 | 0.276708 |
| RSL1D1-liver | CEBPZOS-fat | 0.681818 | 0.020843 | 0.222382 |
| SELENOT-liver | CEBPZOS-fat | 0.763636 | 0.006233 | 0.198679 |
| SLC35F5-liver | CEBPZOS-fat | 0.618182 | 0.042646 | 0.276708 |
| SMIM26-liver | CEBPZOS-fat | 0.827273 | 0.001677 | 0.165242 |
| TXNL1-liver | CEBPZOS-fat | 0.690909 | 0.018565 | 0.218161 |
| UBE2V2-liver | CEBPZOS-fat | 0.637815 | 0.034741 | 0.257828 |
| UBL5-liver | CEBPZOS-fat | 0.690909 | 0.018565 | 0.218161 |
| UFSP2-liver | CEBPZOS-fat | 0.609091 | 0.046696 | 0.28351 |
| VTI1B-liver | CEBPZOS-fat | 0.654545 | 0.028865 | 0.250356 |
| YES1-liver | CEBPZOS-fat | 0.627273 | 0.038845 | 0.267717 |
| EIF3M-liver | CHMP2B-fat | 0.681818 | 0.020843 | 0.222382 |
| RRAGA-liver | CHMP2B-fat | 0.618182 | 0.042646 | 0.276708 |
| RSL1D1-liver | CHMP2B-fat | 0.636364 | 0.035287 | 0.257828 |
| NRAS-liver | CSNK2A2-fat | 0.636364 | 0.035287 | 0.257828 |
| PIGY-liver | CSNK2A2-fat | 0.672727 | 0.023313 | 0.231316 |
| RRAGA-liver | CSNK2A2-fat | 0.654545 | 0.028865 | 0.250356 |
| RRP36-liver | CSNK2A2-fat | 0.7 | 0.016471 | 0.213203 |
| EIF3M-liver | DNAJC7-fat | 0.618182 | 0.042646 | 0.276708 |
| PIGK-liver | DNAJC7-fat | 0.609091 | 0.046696 | 0.28351 |
| RRAGA-liver | DNAJC7-fat | 0.663636 | 0.025984 | 0.237489 |
| RRP36-liver | DNAJC7-fat | 0.718182 | 0.0128 | 0.211941 |
| HNRNPUL1-liver | DPM1-fat | 0.636364 | 0.035287 | 0.257828 |
| KDM7A-liver | DPM1-fat | 0.618182 | 0.042646 | 0.276708 |
| RRAGA-liver | DPM1-fat | 0.672727 | 0.023313 | 0.231316 |
| FBXL5-liver | KIAA1143-fat | 0.636364 | 0.035287 | 0.257828 |
| PGGT1B-liver | KIAA1143-fat | 0.645455 | 0.031963 | 0.251559 |
| SLC35F5-liver | KIAA1143-fat | 0.654545 | 0.028865 | 0.250356 |
| ARCN1-liver | NEMP2-fat | 0.627273 | 0.038845 | 0.267717 |
| COPS3-liver | NEMP2-fat | 0.781818 | 0.004473 | 0.195851 |
| EGLN1-liver | NEMP2-fat | 0.736364 | 0.00976 | 0.211941 |
| EIF3M-liver | NEMP2-fat | 0.618182 | 0.042646 | 0.276708 |
| PIGK-liver | NEMP2-fat | 0.609091 | 0.046696 | 0.28351 |
| PIGY-liver | NEMP2-fat | 0.863636 | 0.000612 | 0.155982 |
| PSMC1-liver | NEMP2-fat | 0.681818 | 0.020843 | 0.222382 |
| RIOK1-liver | NEMP2-fat | 0.690909 | 0.018565 | 0.218161 |
| RRAGA-liver | NEMP2-fat | 0.654545 | 0.028865 | 0.250356 |
| RSL1D1-liver | NEMP2-fat | 0.7 | 0.016471 | 0.213203 |
| SELENOT-liver | NEMP2-fat | 0.727273 | 0.011205 | 0.211941 |
| SMIM26-liver | NEMP2-fat | 0.709091 | 0.014552 | 0.213203 |
| CHCHD2-liver | NET1-fat | 0.681818 | 0.020843 | 0.222382 |
| CMAS-liver | NET1-fat | 0.7 | 0.016471 | 0.213203 |
| COX8A-liver | NET1-fat | 0.709091 | 0.014552 | 0.213203 |
| DERL1-liver | NET1-fat | 0.645455 | 0.031963 | 0.251559 |
| FBXL5-liver | NET1-fat | 0.709091 | 0.014552 | 0.213203 |
| LAMTOR2-liver | NET1-fat | 0.718182 | 0.0128 | 0.211941 |
| MRPS10-liver | NET1-fat | 0.772727 | 0.005299 | 0.195851 |
| MYNN-liver | NET1-fat | 0.636364 | 0.035287 | 0.257828 |
| NDUFA1-liver | NET1-fat | 0.609091 | 0.046696 | 0.28351 |
| NDUFB6-liver | NET1-fat | 0.745455 | 0.008455 | 0.211941 |
| PGGT1B-liver | NET1-fat | 0.727273 | 0.011205 | 0.211941 |
| PIGY-liver | NET1-fat | 0.672727 | 0.023313 | 0.231316 |
| PSMB1-liver | NET1-fat | 0.7 | 0.016471 | 0.213203 |
| PSMB6-liver | NET1-fat | 0.772727 | 0.005299 | 0.195851 |
| SLC35F5-liver | NET1-fat | 0.609091 | 0.046696 | 0.28351 |
| SMIM26-liver | NET1-fat | 0.718182 | 0.0128 | 0.211941 |
| TMTC3-liver | NET1-fat | 0.636364 | 0.035287 | 0.257828 |
| TXNL1-liver | NET1-fat | 0.645455 | 0.031963 | 0.251559 |
| TXNL4A-liver | NET1-fat | 0.7 | 0.016471 | 0.213203 |
| UBE2V2-liver | NET1-fat | 0.642371 | 0.033065 | 0.257828 |
| UBL5-liver | NET1-fat | 0.636364 | 0.035287 | 0.257828 |
| VTI1B-liver | NET1-fat | 0.690909 | 0.018565 | 0.218161 |
| YES1-liver | NET1-fat | 0.736364 | 0.00976 | 0.211941 |
| MYNN-liver | NSMCE3-fat | 0.618182 | 0.042646 | 0.276708 |
| SLC35F5-liver | NSMCE3-fat | 0.609091 | 0.046696 | 0.28351 |
| EIF3M-liver | NUDT19-fat | 0.636364 | 0.035287 | 0.257828 |
| KDM7A-liver | NUDT19-fat | 0.618182 | 0.042646 | 0.276708 |
| ARCN1-liver | OSTF1-fat | 0.645455 | 0.031963 | 0.251559 |
| COPS3-liver | OSTF1-fat | 0.636364 | 0.035287 | 0.257828 |
| EGLN1-liver | OSTF1-fat | 0.681818 | 0.020843 | 0.222382 |
| EIF3M-liver | OSTF1-fat | 0.745455 | 0.008455 | 0.211941 |
| HNRNPUL1-liver | OSTF1-fat | 0.690909 | 0.018565 | 0.218161 |
| KDM7A-liver | OSTF1-fat | 0.718182 | 0.0128 | 0.211941 |
| PIGK-liver | OSTF1-fat | 0.718182 | 0.0128 | 0.211941 |
| PSMC1-liver | OSTF1-fat | 0.718182 | 0.0128 | 0.211941 |
| RAB22A-liver | OSTF1-fat | 0.663636 | 0.025984 | 0.237489 |
| RRAGA-liver | OSTF1-fat | 0.745455 | 0.008455 | 0.211941 |
| RRP36-liver | OSTF1-fat | 0.754545 | 0.007282 | 0.211941 |
| RSL1D1-liver | OSTF1-fat | 0.681818 | 0.020843 | 0.222382 |
| SERINC1-liver | OSTF1-fat | 0.645455 | 0.031963 | 0.251559 |
| SLC35F5-liver | OSTF1-fat | 0.636364 | 0.035287 | 0.257828 |
| TIMM9-liver | OSTF1-fat | 0.645455 | 0.031963 | 0.251559 |
| AATF-liver | PDE9A-fat | 0.627273 | 0.038845 | 0.267717 |
| RAB7A-liver | PDE9A-fat | 0.690909 | 0.018565 | 0.218161 |
| REEP3-liver | PDE9A-fat | 0.609091 | 0.046696 | 0.28351 |
| RIOK1-liver | PDE9A-fat | 0.609091 | 0.046696 | 0.28351 |
| RRAGA-liver | PDE9A-fat | 0.663636 | 0.025984 | 0.237489 |
| RSL1D1-liver | PDE9A-fat | 0.645455 | 0.031963 | 0.251559 |
| SMIM26-liver | PDE9A-fat | 0.618182 | 0.042646 | 0.276708 |
| UBE2V2-liver | PDE9A-fat | 0.615036 | 0.044019 | 0.28351 |
| YES1-liver | PDE9A-fat | 0.672727 | 0.023313 | 0.231316 |
| EGLN1-liver | RPL30-fat | 0.654545 | 0.028865 | 0.250356 |
| EIF3M-liver | RPL30-fat | 0.7 | 0.016471 | 0.213203 |
| KDM7A-liver | RPL30-fat | 0.618182 | 0.042646 | 0.276708 |
| NRAS-liver | RPL30-fat | 0.618182 | 0.042646 | 0.276708 |
| PIGK-liver | RPL30-fat | 0.609091 | 0.046696 | 0.28351 |
| RRAGA-liver | RPL30-fat | 0.645455 | 0.031963 | 0.251559 |
| RRP36-liver | RPL30-fat | 0.690909 | 0.018565 | 0.218161 |
| RSL1D1-liver | RPL30-fat | 0.618182 | 0.042646 | 0.276708 |
| MRPS15-liver | SEC22A-fat | 0.609091 | 0.046696 | 0.28351 |
| CHCHD2-liver | SGCD-fat | 0.636364 | 0.035287 | 0.257828 |
| CMAS-liver | SGCD-fat | 0.709091 | 0.014552 | 0.213203 |
| COX8A-liver | SGCD-fat | 0.663636 | 0.025984 | 0.237489 |
| FBXL5-liver | SGCD-fat | 0.627273 | 0.038845 | 0.267717 |
| LAMTOR2-liver | SGCD-fat | 0.654545 | 0.028865 | 0.250356 |
| MRPS10-liver | SGCD-fat | 0.7 | 0.016471 | 0.213203 |
| NDUFB6-liver | SGCD-fat | 0.7 | 0.016471 | 0.213203 |
| PGGT1B-liver | SGCD-fat | 0.7 | 0.016471 | 0.213203 |
| PSMB6-liver | SGCD-fat | 0.7 | 0.016471 | 0.213203 |
| SLC35F5-liver | SGCD-fat | 0.663636 | 0.025984 | 0.237489 |
| TXNL4A-liver | SGCD-fat | 0.727273 | 0.011205 | 0.211941 |
| VTI1B-liver | SGCD-fat | 0.654545 | 0.028865 | 0.250356 |
| ARCN1-liver | SNX3-fat | 0.818182 | 0.002083 | 0.165242 |
| CHCHD2-liver | SNX3-fat | 0.690909 | 0.018565 | 0.218161 |
| COPS3-liver | SNX3-fat | 0.763636 | 0.006233 | 0.198679 |
| COX8A-liver | SNX3-fat | 0.627273 | 0.038845 | 0.267717 |
| DERL1-liver | SNX3-fat | 0.727273 | 0.011205 | 0.211941 |
| EGLN1-liver | SNX3-fat | 0.809091 | 0.002559 | 0.165242 |
| EIF3M-liver | SNX3-fat | 0.663636 | 0.025984 | 0.237489 |
| HNRNPUL1-liver | SNX3-fat | 0.745455 | 0.008455 | 0.211941 |
| KDM7A-liver | SNX3-fat | 0.8 | 0.00311 | 0.165242 |
| NDUFA1-liver | SNX3-fat | 0.636364 | 0.035287 | 0.257828 |
| NRAS-liver | SNX3-fat | 0.818182 | 0.002083 | 0.165242 |
| PIGK-liver | SNX3-fat | 0.790909 | 0.003746 | 0.173682 |
| PIGY-liver | SNX3-fat | 0.636364 | 0.035287 | 0.257828 |
| POMP-liver | SNX3-fat | 0.790909 | 0.003746 | 0.173682 |
| PSMB1-liver | SNX3-fat | 0.672727 | 0.023313 | 0.231316 |
| PSMC1-liver | SNX3-fat | 0.763636 | 0.006233 | 0.198679 |
| RAB22A-liver | SNX3-fat | 0.890909 | 0.000233 | 0.119064 |
| RAB7A-liver | SNX3-fat | 0.718182 | 0.0128 | 0.211941 |
| RABGGTB-liver | SNX3-fat | 0.818182 | 0.002083 | 0.165242 |
| RIOK1-liver | SNX3-fat | 0.709091 | 0.014552 | 0.213203 |
| RRAGA-liver | SNX3-fat | 0.763636 | 0.006233 | 0.198679 |
| RRP36-liver | SNX3-fat | 0.872727 | 0.000455 | 0.155982 |
| RSL1D1-liver | SNX3-fat | 0.7 | 0.016471 | 0.213203 |
| SELENOT-liver | SNX3-fat | 0.718182 | 0.0128 | 0.211941 |
| SERINC1-liver | SNX3-fat | 0.763636 | 0.006233 | 0.198679 |
| SMIM26-liver | SNX3-fat | 0.609091 | 0.046696 | 0.28351 |
| TAF13-liver | SNX3-fat | 0.7 | 0.016471 | 0.213203 |
| TIMM9-liver | SNX3-fat | 0.8 | 0.00311 | 0.165242 |
| TIPRL-liver | SNX3-fat | 0.663636 | 0.025984 | 0.237489 |
| TMTC3-liver | SNX3-fat | 0.709091 | 0.014552 | 0.213203 |
| TXNL1-liver | SNX3-fat | 0.645455 | 0.031963 | 0.251559 |
| UBL5-liver | SNX3-fat | 0.681818 | 0.020843 | 0.222382 |
| UFSP2-liver | SNX3-fat | 0.736364 | 0.00976 | 0.211941 |
| ARCN1-liver | SRM-fat | 0.7 | 0.016471 | 0.213203 |
| CHCHD2-liver | SRM-fat | 0.772727 | 0.005299 | 0.195851 |
| COPS3-liver | SRM-fat | 0.727273 | 0.011205 | 0.211941 |
| COX8A-liver | SRM-fat | 0.827273 | 0.001677 | 0.165242 |
| DDX1-liver | SRM-fat | 0.690909 | 0.018565 | 0.218161 |
| DERL1-liver | SRM-fat | 0.690909 | 0.018565 | 0.218161 |
| EGLN1-liver | SRM-fat | 0.790909 | 0.003746 | 0.173682 |
| EIF3M-liver | SRM-fat | 0.845455 | 0.001045 | 0.165242 |
| FBXL5-liver | SRM-fat | 0.745455 | 0.008455 | 0.211941 |
| HNRNPUL1-liver | SRM-fat | 0.645455 | 0.031963 | 0.251559 |
| KDM7A-liver | SRM-fat | 0.690909 | 0.018565 | 0.218161 |
| LAMTOR2-liver | SRM-fat | 0.627273 | 0.038845 | 0.267717 |
| MRPS10-liver | SRM-fat | 0.7 | 0.016471 | 0.213203 |
| MYNN-liver | SRM-fat | 0.772727 | 0.005299 | 0.195851 |
| NDUFA1-liver | SRM-fat | 0.763636 | 0.006233 | 0.198679 |
| NRAS-liver | SRM-fat | 0.7 | 0.016471 | 0.213203 |
| PGGT1B-liver | SRM-fat | 0.736364 | 0.00976 | 0.211941 |
| PIGK-liver | SRM-fat | 0.890909 | 0.000233 | 0.119064 |
| PIGY-liver | SRM-fat | 0.681818 | 0.020843 | 0.222382 |
| POMP-liver | SRM-fat | 0.718182 | 0.0128 | 0.211941 |
| PSMB1-liver | SRM-fat | 0.754545 | 0.007282 | 0.211941 |
| PSMB6-liver | SRM-fat | 0.7 | 0.016471 | 0.213203 |
| PSMC1-liver | SRM-fat | 0.854545 | 0.000807 | 0.158232 |
| RAB22A-liver | SRM-fat | 0.690909 | 0.018565 | 0.218161 |
| RAB7A-liver | SRM-fat | 0.727273 | 0.011205 | 0.211941 |
| RABGGTB-liver | SRM-fat | 0.609091 | 0.046696 | 0.28351 |
| RRP36-liver | SRM-fat | 0.772727 | 0.005299 | 0.195851 |
| RSL1D1-liver | SRM-fat | 0.645455 | 0.031963 | 0.251559 |
| SELENOT-liver | SRM-fat | 0.709091 | 0.014552 | 0.213203 |
| SERINC1-liver | SRM-fat | 0.663636 | 0.025984 | 0.237489 |
| SLC35F5-liver | SRM-fat | 0.854545 | 0.000807 | 0.158232 |
| SMIM26-liver | SRM-fat | 0.627273 | 0.038845 | 0.267717 |
| TAF13-liver | SRM-fat | 0.7 | 0.016471 | 0.213203 |
| TIMM9-liver | SRM-fat | 0.845455 | 0.001045 | 0.165242 |
| TMTC3-liver | SRM-fat | 0.836364 | 0.001333 | 0.165242 |
| TXNL1-liver | SRM-fat | 0.709091 | 0.014552 | 0.213203 |
| TXNL4A-liver | SRM-fat | 0.672727 | 0.023313 | 0.231316 |
| UBE2V2-liver | SRM-fat | 0.71982 | 0.012501 | 0.211941 |
| UBL5-liver | SRM-fat | 0.8 | 0.00311 | 0.165242 |
| UFSP2-liver | SRM-fat | 0.727273 | 0.011205 | 0.211941 |
| VTI1B-liver | SRM-fat | 0.645455 | 0.031963 | 0.251559 |
| YES1-liver | SRM-fat | 0.709091 | 0.014552 | 0.213203 |
| ARCN1-liver | TMEM126A-fat | 0.718182 | 0.0128 | 0.211941 |
| CHCHD2-liver | TMEM126A-fat | 0.618182 | 0.042646 | 0.276708 |
| COPS3-liver | TMEM126A-fat | 0.681818 | 0.020843 | 0.222382 |
| COX8A-liver | TMEM126A-fat | 0.645455 | 0.031963 | 0.251559 |
| DDX1-liver | TMEM126A-fat | 0.609091 | 0.046696 | 0.28351 |
| DERL1-liver | TMEM126A-fat | 0.627273 | 0.038845 | 0.267717 |
| EGLN1-liver | TMEM126A-fat | 0.781818 | 0.004473 | 0.195851 |
| EIF3M-liver | TMEM126A-fat | 0.845455 | 0.001045 | 0.165242 |
| HNRNPUL1-liver | TMEM126A-fat | 0.8 | 0.00311 | 0.165242 |
| KDM7A-liver | TMEM126A-fat | 0.745455 | 0.008455 | 0.211941 |
| PIGK-liver | TMEM126A-fat | 0.672727 | 0.023313 | 0.231316 |
| PIGY-liver | TMEM126A-fat | 0.636364 | 0.035287 | 0.257828 |
| POMP-liver | TMEM126A-fat | 0.609091 | 0.046696 | 0.28351 |
| PSMC1-liver | TMEM126A-fat | 0.663636 | 0.025984 | 0.237489 |
| RAB22A-liver | TMEM126A-fat | 0.663636 | 0.025984 | 0.237489 |
| RAB7A-liver | TMEM126A-fat | 0.672727 | 0.023313 | 0.231316 |
| RABGGTB-liver | TMEM126A-fat | 0.636364 | 0.035287 | 0.257828 |
| RIOK1-liver | TMEM126A-fat | 0.754545 | 0.007282 | 0.211941 |
| RRAGA-liver | TMEM126A-fat | 0.827273 | 0.001677 | 0.165242 |
| RRP36-liver | TMEM126A-fat | 0.818182 | 0.002083 | 0.165242 |
| RSL1D1-liver | TMEM126A-fat | 0.909091 | 0.000106 | 0.089754 |
| SELENOT-liver | TMEM126A-fat | 0.672727 | 0.023313 | 0.231316 |
| SERINC1-liver | TMEM126A-fat | 0.681818 | 0.020843 | 0.222382 |
| SLC35F5-liver | TMEM126A-fat | 0.654545 | 0.028865 | 0.250356 |
| TIMM9-liver | TMEM126A-fat | 0.636364 | 0.035287 | 0.257828 |
| UBE2V2-liver | TMEM126A-fat | 0.683373 | 0.02044 | 0.222382 |
| ARCN1-liver | TMEM184C-fat | 0.654545 | 0.028865 | 0.250356 |
| CHCHD2-liver | TMEM184C-fat | 0.718182 | 0.0128 | 0.211941 |
| COPS3-liver | TMEM184C-fat | 0.709091 | 0.014552 | 0.213203 |
| COX8A-liver | TMEM184C-fat | 0.736364 | 0.00976 | 0.211941 |
| EGLN1-liver | TMEM184C-fat | 0.8 | 0.00311 | 0.165242 |
| EIF3M-liver | TMEM184C-fat | 0.863636 | 0.000612 | 0.155982 |
| HNRNPUL1-liver | TMEM184C-fat | 0.627273 | 0.038845 | 0.267717 |
| KDM7A-liver | TMEM184C-fat | 0.718182 | 0.0128 | 0.211941 |
| NRAS-liver | TMEM184C-fat | 0.654545 | 0.028865 | 0.250356 |
| PIGK-liver | TMEM184C-fat | 0.818182 | 0.002083 | 0.165242 |
| PIGY-liver | TMEM184C-fat | 0.681818 | 0.020843 | 0.222382 |
| POMP-liver | TMEM184C-fat | 0.618182 | 0.042646 | 0.276708 |
| PSMB1-liver | TMEM184C-fat | 0.609091 | 0.046696 | 0.28351 |
| PSMC1-liver | TMEM184C-fat | 0.809091 | 0.002559 | 0.165242 |
| RAB22A-liver | TMEM184C-fat | 0.636364 | 0.035287 | 0.257828 |
| RRAGA-liver | TMEM184C-fat | 0.609091 | 0.046696 | 0.28351 |
| RRP36-liver | TMEM184C-fat | 0.736364 | 0.00976 | 0.211941 |
| RSL1D1-liver | TMEM184C-fat | 0.663636 | 0.025984 | 0.237489 |
| SELENOT-liver | TMEM184C-fat | 0.627273 | 0.038845 | 0.267717 |
| SLC35F5-liver | TMEM184C-fat | 0.754545 | 0.007282 | 0.211941 |
| TIMM9-liver | TMEM184C-fat | 0.7 | 0.016471 | 0.213203 |
| TMTC3-liver | TMEM184C-fat | 0.672727 | 0.023313 | 0.231316 |
| UBL5-liver | TMEM184C-fat | 0.663636 | 0.025984 | 0.237489 |
| AATF-liver | TOMM6-fat | 0.709091 | 0.014552 | 0.213203 |
| ARCN1-liver | TOMM6-fat | 0.618182 | 0.042646 | 0.276708 |
| CHCHD2-liver | TOMM6-fat | 0.772727 | 0.005299 | 0.195851 |
| CMAS-liver | TOMM6-fat | 0.672727 | 0.023313 | 0.231316 |
| COPS3-liver | TOMM6-fat | 0.672727 | 0.023313 | 0.231316 |
| COX8A-liver | TOMM6-fat | 0.854545 | 0.000807 | 0.158232 |
| DDX1-liver | TOMM6-fat | 0.645455 | 0.031963 | 0.251559 |
| DERL1-liver | TOMM6-fat | 0.7 | 0.016471 | 0.213203 |
| EGLN1-liver | TOMM6-fat | 0.681818 | 0.020843 | 0.222382 |
| EIF3M-liver | TOMM6-fat | 0.836364 | 0.001333 | 0.165242 |
| FBXL5-liver | TOMM6-fat | 0.863636 | 0.000612 | 0.155982 |
| HNRNPUL1-liver | TOMM6-fat | 0.645455 | 0.031963 | 0.251559 |
| KDM7A-liver | TOMM6-fat | 0.681818 | 0.020843 | 0.222382 |
| LAMTOR2-liver | TOMM6-fat | 0.727273 | 0.011205 | 0.211941 |
| MRPS10-liver | TOMM6-fat | 0.827273 | 0.001677 | 0.165242 |
| MYNN-liver | TOMM6-fat | 0.745455 | 0.008455 | 0.211941 |
| NDUFA1-liver | TOMM6-fat | 0.709091 | 0.014552 | 0.213203 |
| PGGT1B-liver | TOMM6-fat | 0.872727 | 0.000455 | 0.155982 |
| PIGK-liver | TOMM6-fat | 0.8 | 0.00311 | 0.165242 |
| PIGY-liver | TOMM6-fat | 0.627273 | 0.038845 | 0.267717 |
| PSMB1-liver | TOMM6-fat | 0.718182 | 0.0128 | 0.211941 |
| PSMB6-liver | TOMM6-fat | 0.827273 | 0.001677 | 0.165242 |
| PSMC1-liver | TOMM6-fat | 0.818182 | 0.002083 | 0.165242 |
| RAB7A-liver | TOMM6-fat | 0.645455 | 0.031963 | 0.251559 |
| SELENOT-liver | TOMM6-fat | 0.690909 | 0.018565 | 0.218161 |
| SERINC1-liver | TOMM6-fat | 0.609091 | 0.046696 | 0.28351 |
| SLC35F5-liver | TOMM6-fat | 0.945455 | 1.12E-05 | 0.028517 |
| SMIM26-liver | TOMM6-fat | 0.645455 | 0.031963 | 0.251559 |
| TIMM9-liver | TOMM6-fat | 0.663636 | 0.025984 | 0.237489 |
| TMTC3-liver | TOMM6-fat | 0.736364 | 0.00976 | 0.211941 |
| TXNL1-liver | TOMM6-fat | 0.7 | 0.016471 | 0.213203 |
| TXNL4A-liver | TOMM6-fat | 0.8 | 0.00311 | 0.165242 |
| UBE2V2-liver | TOMM6-fat | 0.692485 | 0.018189 | 0.218161 |
| UBL5-liver | TOMM6-fat | 0.727273 | 0.011205 | 0.211941 |
| VTI1B-liver | TOMM6-fat | 0.772727 | 0.005299 | 0.195851 |
| YES1-liver | TOMM6-fat | 0.745455 | 0.008455 | 0.211941 |
| AATF-liver | ZNF280C-fat | 0.8 | 0.00311 | 0.165242 |
| COPS3-liver | ZNF280C-fat | 0.627273 | 0.038845 | 0.267717 |
| MRPS15-liver | ZNF280C-fat | 0.645455 | 0.031963 | 0.251559 |
| RAB7A-liver | ZNF280C-fat | 0.672727 | 0.023313 | 0.231316 |
| REEP3-liver | ZNF280C-fat | 0.718182 | 0.0128 | 0.211941 |
| RRAGA-liver | ZNF280C-fat | 0.736364 | 0.00976 | 0.211941 |
| SELENOT-liver | ZNF280C-fat | 0.654545 | 0.028865 | 0.250356 |
| SERINC1-liver | ZNF280C-fat | 0.627273 | 0.038845 | 0.267717 |
| TIPRL-liver | ZNF280C-fat | 0.8 | 0.00311 | 0.165242 |
| PIGY-liver | ZNF823-fat | 0.690909 | 0.018565 | 0.218161 |
| SLC35F5-liver | ZNF823-fat | 0.654545 | 0.028865 | 0.250356 |

**Supplement Table 5 represents the top 5% in Liver-2 gene centrality and in Fat-1, with calculated Spearman correlations between the two groups, focusing only on strong correlations of >0.6 with a p<0.05.**

**LIVER INFLAMMATION**

**Supplement Table-6**

| **Liver-4** | **Fat-39** | **Correlation** | **p_value** | **adjusted_p_value** |
| --- | --- | --- | --- | --- |
| SEMA5A-liver | CRYBB2-fat | -0.78182 | 0.004473 | 0.067775 |
| THSD4-liver | CRYBB2-fat | -0.7 | 0.016471 | 0.12478 |
| WDR19-liver | CRYBB2-fat | -0.68182 | 0.020843 | 0.142759 |
| WNK2-liver | CRYBB2-fat | -0.68182 | 0.020843 | 0.142759 |
| RAB3IP-liver | DMTN-fat | -0.69091 | 0.018565 | 0.133561 |
| SEMA5A-liver | DMTN-fat | -0.80909 | 0.002559 | 0.054438 |
| SUPT3H-liver | DMTN-fat | -0.71818 | 0.0128 | 0.105782 |
| THSD4-liver | DMTN-fat | -0.84545 | 0.001045 | 0.03871 |
| WDR19-liver | DMTN-fat | -0.82727 | 0.001677 | 0.042999 |
| WNK2-liver | DMTN-fat | -0.65455 | 0.028865 | 0.178177 |
| ANXA8L1-liver | KRT36-fat | -0.8 | 0.00311 | 0.058687 |
| BACE2-liver | KRT36-fat | -0.73636 | 0.00976 | 0.090366 |
| C12orf75-liver | KRT36-fat | -0.65455 | 0.028865 | 0.178177 |
| C1orf116-liver | KRT36-fat | -0.78182 | 0.004473 | 0.067775 |
| CATSPERB-liver | KRT36-fat | -0.74545 | 0.008455 | 0.085401 |
| CBR3-liver | KRT36-fat | -0.69091 | 0.018565 | 0.133561 |
| CCND2-liver | KRT36-fat | -0.80909 | 0.002559 | 0.054438 |
| CEP112-liver | KRT36-fat | -0.72727 | 0.011205 | 0.098289 |
| CFAP221-liver | KRT36-fat | -0.61818 | 0.042646 | 0.249389 |
| COL4A2-liver | KRT36-fat | -0.87273 | 0.000455 | 0.03871 |
| DMKN-liver | KRT36-fat | -0.65455 | 0.028865 | 0.178177 |
| DOCK1-liver | KRT36-fat | -0.77273 | 0.005299 | 0.068825 |
| DYNC1I2-liver | KRT36-fat | -0.84545 | 0.001045 | 0.03871 |
| EDN1-liver | KRT36-fat | -0.77273 | 0.005299 | 0.068825 |
| EFNA5-liver | KRT36-fat | -0.62727 | 0.038845 | 0.229854 |
| ENTPD1-liver | KRT36-fat | -0.80909 | 0.002559 | 0.054438 |
| FAM227A-liver | KRT36-fat | -0.74715 | 0.008226 | 0.085401 |
| FSTL1-liver | KRT36-fat | -0.79091 | 0.003746 | 0.064588 |
| FUT4-liver | KRT36-fat | -0.70909 | 0.014552 | 0.113688 |
| GALNT7-liver | KRT36-fat | -0.66364 | 0.025984 | 0.166565 |
| GK5-liver | KRT36-fat | -0.7 | 0.016471 | 0.12478 |
| GRAMD1B-liver | KRT36-fat | -0.75455 | 0.007282 | 0.080022 |
| GSTP1-liver | KRT36-fat | -0.84545 | 0.001045 | 0.03871 |
| GUCY1B1-liver | KRT36-fat | -0.72727 | 0.011205 | 0.098289 |
| GULP1-liver | KRT36-fat | -0.69091 | 0.018565 | 0.133561 |
| HDAC7-liver | KRT36-fat | -0.74545 | 0.008455 | 0.085401 |
| HOXB2-liver | KRT36-fat | -0.77273 | 0.005299 | 0.068825 |
| HSPG2-liver | KRT36-fat | -0.70909 | 0.014552 | 0.113688 |
| IQGAP1-liver | KRT36-fat | -0.73636 | 0.00976 | 0.090366 |
| ITGA2-liver | KRT36-fat | -0.73636 | 0.00976 | 0.090366 |
| ITGA3-liver | KRT36-fat | -0.77273 | 0.005299 | 0.068825 |
| ITPR3-liver | KRT36-fat | -0.78182 | 0.004473 | 0.067775 |
| LRRC49-liver | KRT36-fat | -0.73636 | 0.00976 | 0.090366 |
| LXN-liver | KRT36-fat | -0.7 | 0.016471 | 0.12478 |
| MAP3K1-liver | KRT36-fat | -0.71818 | 0.0128 | 0.105782 |
| MAP9-liver | KRT36-fat | -0.73636 | 0.00976 | 0.090366 |
| MCTP2-liver | KRT36-fat | -0.72727 | 0.011205 | 0.098289 |
| MLLT3-liver | KRT36-fat | -0.76364 | 0.006233 | 0.074203 |
| MMP14-liver | KRT36-fat | -0.83636 | 0.001333 | 0.041662 |
| MXRA8-liver | KRT36-fat | -0.84545 | 0.001045 | 0.03871 |
| PCDHB15-liver | KRT36-fat | -0.60909 | 0.046696 | 0.266833 |
| PCNX2-liver | KRT36-fat | -0.86364 | 0.000612 | 0.03871 |
| PDGFRB-liver | KRT36-fat | -0.73636 | 0.00976 | 0.090366 |
| PHLDB1-liver | KRT36-fat | -0.69091 | 0.018565 | 0.133561 |
| PIWIL4-liver | KRT36-fat | -0.81818 | 0.002083 | 0.049599 |
| PKD2-liver | KRT36-fat | -0.77273 | 0.005299 | 0.068825 |
| PKM-liver | KRT36-fat | -0.82727 | 0.001677 | 0.042999 |
| PLPP2-liver | KRT36-fat | -0.60909 | 0.046696 | 0.266833 |
| PLXDC2-liver | KRT36-fat | -0.84545 | 0.001045 | 0.03871 |
| PMEPA1-liver | KRT36-fat | -0.72727 | 0.011205 | 0.098289 |
| PMP22-liver | KRT36-fat | -0.79091 | 0.003746 | 0.064588 |
| PODXL-liver | KRT36-fat | -0.84545 | 0.001045 | 0.03871 |
| PRAG1-liver | KRT36-fat | -0.86364 | 0.000612 | 0.03871 |
| PRDM5-liver | KRT36-fat | -0.89091 | 0.000233 | 0.03871 |
| PROM1-liver | KRT36-fat | -0.66364 | 0.025984 | 0.166565 |
| RARRES1-liver | KRT36-fat | -0.83636 | 0.001333 | 0.041662 |
| RASAL2-liver | KRT36-fat | -0.82727 | 0.001677 | 0.042999 |
| ROBO1-liver | KRT36-fat | -0.77273 | 0.005299 | 0.068825 |
| RUNX1-liver | KRT36-fat | -0.79091 | 0.003746 | 0.064588 |
| S100A11-liver | KRT36-fat | -0.85455 | 0.000807 | 0.03871 |
| S100A6-liver | KRT36-fat | -0.60909 | 0.046696 | 0.266833 |
| SCTR-liver | KRT36-fat | -0.66364 | 0.025984 | 0.166565 |
| SEL1L3-liver | KRT36-fat | -0.64545 | 0.031963 | 0.193714 |
| SMC5-liver | KRT36-fat | -0.70909 | 0.014552 | 0.113688 |
| SOX4-liver | KRT36-fat | -0.71818 | 0.0128 | 0.105782 |
| SPINT1-liver | KRT36-fat | -0.68182 | 0.020843 | 0.142759 |
| SPINT2-liver | KRT36-fat | -0.77273 | 0.005299 | 0.068825 |
| STK39-liver | KRT36-fat | -0.8 | 0.00311 | 0.058687 |
| STXBP1-liver | KRT36-fat | -0.66364 | 0.025984 | 0.166565 |
| SYNE2-liver | KRT36-fat | -0.67273 | 0.023313 | 0.154391 |
| TC2N-liver | KRT36-fat | -0.7 | 0.016471 | 0.12478 |
| TIMP2-liver | KRT36-fat | -0.70909 | 0.014552 | 0.113688 |
| VIM-liver | KRT36-fat | -0.65455 | 0.028865 | 0.178177 |
| ZNF532-liver | KRT36-fat | -0.85455 | 0.000807 | 0.03871 |
| RAB3IP-liver | RILP-fat | -0.68182 | 0.020843 | 0.142759 |
| SEMA5A-liver | RILP-fat | -0.79091 | 0.003746 | 0.064588 |
| SUPT3H-liver | RILP-fat | -0.71818 | 0.0128 | 0.105782 |
| THSD4-liver | RILP-fat | -0.81818 | 0.002083 | 0.049599 |
| WDR19-liver | RILP-fat | -0.75455 | 0.007282 | 0.080022 |
| WNK2-liver | RILP-fat | -0.62727 | 0.038845 | 0.229854 |
| SEMA5A-liver | RPL39L-fat | -0.66364 | 0.025984 | 0.166565 |
| SUPT3H-liver | RPL39L-fat | -0.64545 | 0.031963 | 0.193714 |
| THSD4-liver | RPL39L-fat | -0.79091 | 0.003746 | 0.064588 |
| WDR19-liver | RPL39L-fat | -0.76364 | 0.006233 | 0.074203 |
| ANXA8-liver | THRSP-fat | -0.68182 | 0.020843 | 0.142759 |
| ANXA8L1-liver | THRSP-fat | -0.86364 | 0.000612 | 0.03871 |
| BACE2-liver | THRSP-fat | -0.75455 | 0.007282 | 0.080022 |
| C12orf75-liver | THRSP-fat | -0.73636 | 0.00976 | 0.090366 |
| C1orf116-liver | THRSP-fat | -0.81818 | 0.002083 | 0.049599 |
| CATSPERB-liver | THRSP-fat | -0.69091 | 0.018565 | 0.133561 |
| CBR3-liver | THRSP-fat | -0.74545 | 0.008455 | 0.085401 |
| CCND2-liver | THRSP-fat | -0.84545 | 0.001045 | 0.03871 |
| CEP112-liver | THRSP-fat | -0.87273 | 0.000455 | 0.03871 |
| CFAP221-liver | THRSP-fat | -0.68182 | 0.020843 | 0.142759 |
| COL4A2-liver | THRSP-fat | -0.88182 | 0.00033 | 0.03871 |
| CXCL6-liver | THRSP-fat | -0.61818 | 0.042646 | 0.249389 |
| DMKN-liver | THRSP-fat | -0.8 | 0.00311 | 0.058687 |
| DOCK1-liver | THRSP-fat | -0.8 | 0.00311 | 0.058687 |
| DYNC1I2-liver | THRSP-fat | -0.83636 | 0.001333 | 0.041662 |
| EDN1-liver | THRSP-fat | -0.78182 | 0.004473 | 0.067775 |
| EHF-liver | THRSP-fat | -0.75455 | 0.007282 | 0.080022 |
| ENTPD1-liver | THRSP-fat | -0.85455 | 0.000807 | 0.03871 |
| ESRP1-liver | THRSP-fat | -0.67273 | 0.023313 | 0.154391 |
| FAM227A-liver | THRSP-fat | -0.77449 | 0.005131 | 0.068825 |
| FSTL1-liver | THRSP-fat | -0.82727 | 0.001677 | 0.042999 |
| FUT4-liver | THRSP-fat | -0.84545 | 0.001045 | 0.03871 |
| GALNT7-liver | THRSP-fat | -0.67273 | 0.023313 | 0.154391 |
| GK5-liver | THRSP-fat | -0.63636 | 0.035287 | 0.212572 |
| GRAMD1B-liver | THRSP-fat | -0.89091 | 0.000233 | 0.03871 |
| GSTP1-liver | THRSP-fat | -0.87273 | 0.000455 | 0.03871 |
| GUCY1B1-liver | THRSP-fat | -0.76364 | 0.006233 | 0.074203 |
| GULP1-liver | THRSP-fat | -0.72727 | 0.011205 | 0.098289 |
| HDAC7-liver | THRSP-fat | -0.82727 | 0.001677 | 0.042999 |
| HOXB2-liver | THRSP-fat | -0.84545 | 0.001045 | 0.03871 |
| HSPG2-liver | THRSP-fat | -0.74545 | 0.008455 | 0.085401 |
| IQGAP1-liver | THRSP-fat | -0.74545 | 0.008455 | 0.085401 |
| ITGA2-liver | THRSP-fat | -0.77273 | 0.005299 | 0.068825 |
| ITGA3-liver | THRSP-fat | -0.78182 | 0.004473 | 0.067775 |
| ITGB8-liver | THRSP-fat | -0.65455 | 0.028865 | 0.178177 |
| ITPR3-liver | THRSP-fat | -0.80909 | 0.002559 | 0.054438 |
| LRRC49-liver | THRSP-fat | -0.70909 | 0.014552 | 0.113688 |
| LXN-liver | THRSP-fat | -0.74545 | 0.008455 | 0.085401 |
| MAP3K1-liver | THRSP-fat | -0.72727 | 0.011205 | 0.098289 |
| MAP9-liver | THRSP-fat | -0.78182 | 0.004473 | 0.067775 |
| MCTP2-liver | THRSP-fat | -0.67273 | 0.023313 | 0.154391 |
| MLLT3-liver | THRSP-fat | -0.70909 | 0.014552 | 0.113688 |
| MMP14-liver | THRSP-fat | -0.85455 | 0.000807 | 0.03871 |
| MXRA8-liver | THRSP-fat | -0.8 | 0.00311 | 0.058687 |
| PCDHB15-liver | THRSP-fat | -0.62727 | 0.038845 | 0.229854 |
| PCNX2-liver | THRSP-fat | -0.80909 | 0.002559 | 0.054438 |
| PDGFRB-liver | THRSP-fat | -0.75455 | 0.007282 | 0.080022 |
| PHLDB1-liver | THRSP-fat | -0.67273 | 0.023313 | 0.154391 |
| PIWIL4-liver | THRSP-fat | -0.84545 | 0.001045 | 0.03871 |
| PKD2-liver | THRSP-fat | -0.83636 | 0.001333 | 0.041662 |
| PKM-liver | THRSP-fat | -0.8 | 0.00311 | 0.058687 |
| PLPP2-liver | THRSP-fat | -0.76364 | 0.006233 | 0.074203 |
| PLXDC2-liver | THRSP-fat | -0.77273 | 0.005299 | 0.068825 |
| PMEPA1-liver | THRSP-fat | -0.76364 | 0.006233 | 0.074203 |
| PMP22-liver | THRSP-fat | -0.71818 | 0.0128 | 0.105782 |
| PODXL-liver | THRSP-fat | -0.82727 | 0.001677 | 0.042999 |
| PRAG1-liver | THRSP-fat | -0.85455 | 0.000807 | 0.03871 |
| PRDM5-liver | THRSP-fat | -0.89091 | 0.000233 | 0.03871 |
| PROM1-liver | THRSP-fat | -0.78182 | 0.004473 | 0.067775 |
| RARRES1-liver | THRSP-fat | -0.75455 | 0.007282 | 0.080022 |
| RASAL2-liver | THRSP-fat | -0.83636 | 0.001333 | 0.041662 |
| ROBO1-liver | THRSP-fat | -0.73636 | 0.00976 | 0.090366 |
| RUNX1-liver | THRSP-fat | -0.85455 | 0.000807 | 0.03871 |
| S100A11-liver | THRSP-fat | -0.82727 | 0.001677 | 0.042999 |
| S100A6-liver | THRSP-fat | -0.76364 | 0.006233 | 0.074203 |
| SCTR-liver | THRSP-fat | -0.69091 | 0.018565 | 0.133561 |
| SEL1L3-liver | THRSP-fat | -0.75455 | 0.007282 | 0.080022 |
| SLC34A2-liver | THRSP-fat | -0.68182 | 0.020843 | 0.142759 |
| SMC5-liver | THRSP-fat | -0.74545 | 0.008455 | 0.085401 |
| SOX4-liver | THRSP-fat | -0.78182 | 0.004473 | 0.067775 |
| SPINT1-liver | THRSP-fat | -0.69091 | 0.018565 | 0.133561 |
| SPINT2-liver | THRSP-fat | -0.77273 | 0.005299 | 0.068825 |
| STK33-liver | THRSP-fat | -0.70909 | 0.014552 | 0.113688 |
| STK39-liver | THRSP-fat | -0.86364 | 0.000612 | 0.03871 |
| STXBP1-liver | THRSP-fat | -0.65455 | 0.028865 | 0.178177 |
| SYNE2-liver | THRSP-fat | -0.73636 | 0.00976 | 0.090366 |
| TACC1-liver | THRSP-fat | -0.60909 | 0.046696 | 0.266833 |
| TC2N-liver | THRSP-fat | -0.64545 | 0.031963 | 0.193714 |
| TIMP2-liver | THRSP-fat | -0.71818 | 0.0128 | 0.105782 |
| VIM-liver | THRSP-fat | -0.71818 | 0.0128 | 0.105782 |
| ZNF532-liver | THRSP-fat | -0.76364 | 0.006233 | 0.074203 |

**Supplement Table 6 represents the top 5% in Liver-4 gene centrality and in Fat-39, with calculated Spearman correlations between the two groups, focusing only on strong correlations of <-0.6 with a p<0.05.**

**LIVER STEATOSIS**

**Supplement Table-7**

| **Liver-5** | **Muscle-63** | **Correlation** | **p_value** | **adjusted_p_value** |
| --- | --- | --- | --- | --- |
| ARGLU1-liver | ARHGDIG-muscle | 0.736364 | 0.00976 | 0.042942 |
| DMTF1-liver | ARHGDIG-muscle | 0.718182 | 0.0128 | 0.054152 |
| EZH1-liver | ARHGDIG-muscle | 0.763636 | 0.006233 | 0.034282 |
| H2BC14-liver | ARHGDIG-muscle | 0.790909 | 0.003746 | 0.029434 |
| LENG8-liver | ARHGDIG-muscle | 0.818182 | 0.002083 | 0.022915 |
| LENG9-liver | ARHGDIG-muscle | 0.7 | 0.016471 | 0.060394 |
| PCGF3-liver | ARHGDIG-muscle | 0.745455 | 0.008455 | 0.038751 |
| ZNF841-liver | ARHGDIG-muscle | 0.709091 | 0.014552 | 0.055197 |
| ARGLU1-liver | NECTIN1-muscle | 0.790909 | 0.003746 | 0.029434 |
| CADM2-liver | NECTIN1-muscle | 0.790909 | 0.003746 | 0.029434 |
| CASTOR2-liver | NECTIN1-muscle | 0.618182 | 0.042646 | 0.106614 |
| PCGF3-liver | NECTIN1-muscle | 0.8 | 0.00311 | 0.029434 |
| STRN4-liver | NECTIN1-muscle | 0.681818 | 0.020843 | 0.065506 |
| TSPOAP1-liver | NECTIN1-muscle | 0.672727 | 0.023313 | 0.065755 |
| VSTM4-liver | NECTIN1-muscle | 0.618182 | 0.042646 | 0.106614 |
| ARGLU1-liver | OR4K14-muscle | 0.845455 | 0.001045 | 0.019162 |
| C2orf68-liver | OR4K14-muscle | 0.763636 | 0.006233 | 0.034282 |
| CADM2-liver | OR4K14-muscle | 0.818182 | 0.002083 | 0.022915 |
| CASTOR2-liver | OR4K14-muscle | 0.681818 | 0.020843 | 0.065506 |
| DMTF1-liver | OR4K14-muscle | 0.781818 | 0.004473 | 0.030753 |
| EZH1-liver | OR4K14-muscle | 0.672727 | 0.023313 | 0.065755 |
| H2AC20-liver | OR4K14-muscle | 0.772727 | 0.005299 | 0.032386 |
| LENG8-liver | OR4K14-muscle | 0.690909 | 0.018565 | 0.065506 |
| LENG9-liver | OR4K14-muscle | 0.772727 | 0.005299 | 0.032386 |
| PCGF3-liver | OR4K14-muscle | 0.872727 | 0.000455 | 0.016669 |
| SCARA3-liver | OR4K14-muscle | 0.645455 | 0.031963 | 0.085754 |
| STRN4-liver | OR4K14-muscle | 0.781818 | 0.004473 | 0.030753 |
| TEKT1-liver | OR4K14-muscle | 0.709091 | 0.014552 | 0.055197 |
| TSPOAP1-liver | OR4K14-muscle | 0.818182 | 0.002083 | 0.022915 |
| TSSK4-liver | OR4K14-muscle | 0.745455 | 0.008455 | 0.038751 |
| VSTM4-liver | OR4K14-muscle | 0.709091 | 0.014552 | 0.055197 |
| ZNF841-liver | OR4K14-muscle | 0.672727 | 0.023313 | 0.065755 |
| ARGLU1-liver | SEMA6B-muscle | 0.745455 | 0.008455 | 0.038751 |
| C2orf68-liver | SEMA6B-muscle | 0.681818 | 0.020843 | 0.065506 |
| CASTOR2-liver | SEMA6B-muscle | 0.645455 | 0.031963 | 0.085754 |
| CIZ1-liver | SEMA6B-muscle | 0.745455 | 0.008455 | 0.038751 |
| DMTF1-liver | SEMA6B-muscle | 0.818182 | 0.002083 | 0.022915 |
| GOLGA6L10-liver | SEMA6B-muscle | 0.681818 | 0.020843 | 0.065506 |
| LENG8-liver | SEMA6B-muscle | 0.890909 | 0.000233 | 0.01284 |
| LENG9-liver | SEMA6B-muscle | 0.9 | 0.00016 | 0.01284 |
| PCGF3-liver | SEMA6B-muscle | 0.845455 | 0.001045 | 0.019162 |
| SCARA3-liver | SEMA6B-muscle | 0.636364 | 0.035287 | 0.092418 |
| STRN4-liver | SEMA6B-muscle | 0.672727 | 0.023313 | 0.065755 |
| TSPOAP1-liver | SEMA6B-muscle | 0.609091 | 0.046696 | 0.114145 |
| VSTM4-liver | SEMA6B-muscle | 0.863636 | 0.000612 | 0.016822 |

**Supplement Table 7 represents the top 5% in Liver-5 gene centrality and Muscle-63, with calculated Spearman correlations between the two groups, focusing only on strong correlations of >0.60.6 with a p<0.05. Liver-5 gene cluster correlated with liver histological steatosis (-0.9, p<0.01) and Muscle-63 with liver histological steatosis (-0.69, p<0.05) and the correlation between the two gene groups was 0.7, p<0.05.**

**Supplement Table-8**

| **Liver-39** | **Muscle-54** | **Correlation** | **p_value** | **adjusted_p_value** |
| --- | --- | --- | --- | --- |
| NACC1-liver | CDH1-muscle | -0.83636 | 0.001333 | 0.012793 |
| USP6NL-liver | CDH1-muscle | -0.60909 | 0.046696 | 0.097283 |
| ADRA2B-liver | CTXN2-muscle | -0.81818 | 0.002083 | 0.012793 |
| LAP3-liver | CTXN2-muscle | -0.82727 | 0.001677 | 0.012793 |
| NACC1-liver | CTXN2-muscle | -0.75455 | 0.007282 | 0.026007 |
| STK40-liver | CTXN2-muscle | -0.63636 | 0.035287 | 0.080198 |
| USP6NL-liver | CTXN2-muscle | -0.70909 | 0.014552 | 0.040422 |
| ADRA2B-liver | NES-muscle | -0.68182 | 0.020843 | 0.052107 |
| LAP3-liver | NES-muscle | -0.75455 | 0.007282 | 0.026007 |
| ADRA2B-liver | PWP2-muscle | -0.80909 | 0.002559 | 0.012793 |
| LAP3-liver | PWP2-muscle | -0.71818 | 0.0128 | 0.039999 |
| NACC1-liver | PWP2-muscle | -0.82727 | 0.001677 | 0.012793 |

**Supplement Table 8 represents the top 5% in Liver-39 gene centrality and Muscle-54, with calculated Spearman correlations between the two groups, focusing only on strong correlations of <-0.81 with a p<0.05. Liver-39 gene cluster correlated with liver histological steatosis (0.75, p<0.05) and Muscle-54 with liver histological steatosis (-0.67, p<0.05) and the correlation between the two gene groups was -0.81, p<0.01.**

**Supplement Table 9**

| **Liver-5** | **Fat-49** | **Correlation** | **p_value** | **adjusted_p_value** |
| --- | --- | --- | --- | --- |
| ARGLU1-liver | HPCA-fat | 0.772727 | 0.005299 | 0.048948 |
| C2orf68-liver | HPCA-fat | 0.790909 | 0.003746 | 0.045785 |
| CADM2-liver | HPCA-fat | 0.809091 | 0.002559 | 0.045785 |
| CASTOR2-liver | HPCA-fat | 0.881818 | 0.00033 | 0.033643 |
| CIZ1-liver | HPCA-fat | 0.718182 | 0.0128 | 0.054152 |
| DMTF1-liver | HPCA-fat | 0.736364 | 0.00976 | 0.051121 |
| H2AC20-liver | HPCA-fat | 0.754545 | 0.007282 | 0.048948 |
| LENG9-liver | HPCA-fat | 0.763636 | 0.006233 | 0.048948 |
| PCGF3-liver | HPCA-fat | 0.790909 | 0.003746 | 0.045785 |
| SCARA3-liver | HPCA-fat | 0.609091 | 0.046696 | 0.119369 |
| STRN4-liver | HPCA-fat | 0.863636 | 0.000612 | 0.033643 |
| TSPOAP1-liver | HPCA-fat | 0.845455 | 0.001045 | 0.036893 |
| TSSK4-liver | HPCA-fat | 0.609091 | 0.046696 | 0.119369 |
| VSTM4-liver | HPCA-fat | 0.827273 | 0.001677 | 0.036893 |
| ARGLU1-liver | NOP53-fat | 0.627273 | 0.038845 | 0.109564 |
| C2orf68-liver | NOP53-fat | 0.781818 | 0.004473 | 0.048948 |
| EZH1-liver | NOP53-fat | 0.754545 | 0.007282 | 0.048948 |
| LENG8-liver | NOP53-fat | 0.718182 | 0.0128 | 0.054152 |
| LENG9-liver | NOP53-fat | 0.727273 | 0.011205 | 0.054152 |
| PCGF3-liver | NOP53-fat | 0.627273 | 0.038845 | 0.109564 |
| SCARA3-liver | NOP53-fat | 0.690909 | 0.018565 | 0.065876 |
| TEKT1-liver | NOP53-fat | 0.627273 | 0.038845 | 0.109564 |
| ZNF841-liver | NOP53-fat | 0.7 | 0.016471 | 0.060394 |
| ADAM20-liver | SPDYE2-fat | 0.681818 | 0.020843 | 0.069476 |
| C2orf68-liver | SPDYE2-fat | 0.7 | 0.016471 | 0.060394 |
| EZH1-liver | SPDYE2-fat | 0.763636 | 0.006233 | 0.048948 |
| H2AC20-liver | SPDYE2-fat | 0.672727 | 0.023313 | 0.075425 |
| SCARA3-liver | SPDYE2-fat | 0.654545 | 0.028865 | 0.088197 |
| TEKT1-liver | SPDYE2-fat | 0.718182 | 0.0128 | 0.054152 |
| ZNF841-liver | SPDYE2-fat | 0.827273 | 0.001677 | 0.036893 |
| ADAM20-liver | SPDYE2B-fat | 0.681818 | 0.020843 | 0.069476 |
| C2orf68-liver | SPDYE2B-fat | 0.663636 | 0.025984 | 0.081664 |
| EZH1-liver | SPDYE2B-fat | 0.745455 | 0.008455 | 0.048948 |
| SCARA3-liver | SPDYE2B-fat | 0.609091 | 0.046696 | 0.119369 |
| TEKT1-liver | SPDYE2B-fat | 0.718182 | 0.0128 | 0.054152 |
| ZNF841-liver | SPDYE2B-fat | 0.8 | 0.00311 | 0.045785 |
| C2orf68-liver | UPK3BL1-fat | 0.763636 | 0.006233 | 0.048948 |
| EZH1-liver | UPK3BL1-fat | 0.736364 | 0.00976 | 0.051121 |
| LENG8-liver | UPK3BL1-fat | 0.7 | 0.016471 | 0.060394 |
| LENG9-liver | UPK3BL1-fat | 0.7 | 0.016471 | 0.060394 |
| SCARA3-liver | UPK3BL1-fat | 0.745455 | 0.008455 | 0.048948 |
| TEKT1-liver | UPK3BL1-fat | 0.618182 | 0.042646 | 0.117275 |
| ZNF841-liver | UPK3BL1-fat | 0.745455 | 0.008455 | 0.048948 |

**Supplement Table 9 represents the top 5% in Liver-5 gene centrality and in Fat-49, with calculated Spearman correlations between the two groups, focusing only on strong correlations of >0.6 with a p<0.05.**

**Supplement Table-10**

| **Liver-39** | **Fat-5** | **Correlation** | **p_value** | **adjusted_p_value** |
| --- | --- | --- | --- | --- |
| ADRA2B-liver | FGF10-fat | -0.61818 | 0.042646 | 0.056113 |
| LAP3-liver | FGF10-fat | -0.71818 | 0.0128 | 0.02909 |
| USP6NL-liver | FGF10-fat | -0.70909 | 0.014552 | 0.030317 |
| ADRA2B-liver | NHLH1-fat | -0.63636 | 0.035287 | 0.04901 |
| LAP3-liver | NHLH1-fat | -0.74545 | 0.008455 | 0.026421 |
| NACC1-liver | NHLH1-fat | -0.6 | 0.051003 | 0.063754 |
| STK40-liver | NHLH1-fat | -0.82727 | 0.001677 | 0.008385 |
| USP6NL-liver | NHLH1-fat | -0.83636 | 0.001333 | 0.008332 |
| LAP3-liver | SMC5-fat | -0.66364 | 0.025984 | 0.043307 |
| USP6NL-liver | SMC5-fat | -0.73636 | 0.00976 | 0.02711 |
| LAP3-liver | WSB1-fat | -0.77273 | 0.005299 | 0.022081 |
| STK40-liver | WSB1-fat | -0.72727 | 0.011205 | 0.028012 |
| USP6NL-liver | WSB1-fat | -0.88182 | 0.00033 | 0.005683 |
| ADRA2B-liver | ZKSCAN1-fat | -0.74545 | 0.008455 | 0.026421 |
| STK40-liver | ZKSCAN1-fat | -0.63636 | 0.035287 | 0.04901 |
| USP6NL-liver | ZKSCAN1-fat | -0.69091 | 0.018565 | 0.033152 |

| **Liver-39** | **Fat-43** | **Correlation** | **p_value** | **adjusted_p_value** |
| --- | --- | --- | --- | --- |
| ADRA2B-liver | DMAC1-fat | 0.718182 | 0.0128 | 0.053332 |
| LAP3-liver | DMAC1-fat | 0.763636 | 0.006233 | 0.048798 |
| STK40-liver | DMAC1-fat | 0.809091 | 0.002559 | 0.046826 |
| USP6NL-liver | DMAC1-fat | 0.745455 | 0.008455 | 0.048798 |
| ADRA2B-liver | NDUFAF8-fat | 0.7 | 0.016471 | 0.058825 |
| LAP3-liver | NDUFAF8-fat | 0.790909 | 0.003746 | 0.046826 |
| STK40-liver | NDUFAF8-fat | 0.736364 | 0.00976 | 0.048798 |
| USP6NL-liver | NDUFAF8-fat | 0.663636 | 0.025984 | 0.0812 |

| **Liver-39** | **Fat-19** | **Correlation** | **p_value** | **adjusted_p_value** |
| --- | --- | --- | --- | --- |
| STK40-liver | AP5M1-fat | 0.645455 | 0.031963 | 0.140324 |
| STK40-liver | BMPR2-fat | 0.736364 | 0.00976 | 0.097595 |
| USP6NL-liver | BMPR2-fat | 0.636364 | 0.035287 | 0.15123 |
| ADRA2B-liver | C2CD6-fat | 0.618182 | 0.042646 | 0.159921 |
| LAP3-liver | C2CD6-fat | 0.736364 | 0.00976 | 0.097595 |
| STK40-liver | C2CD6-fat | 0.781818 | 0.004473 | 0.073197 |
| USP6NL-liver | C2CD6-fat | 0.627273 | 0.038845 | 0.152003 |
| LAP3-liver | EDNRA-fat | 0.663636 | 0.025984 | 0.126409 |
| USP6NL-liver | EDNRA-fat | 0.645455 | 0.031963 | 0.140324 |
| LAP3-liver | ELMOD2-fat | 0.681818 | 0.020843 | 0.117241 |
| STK40-liver | ELMOD2-fat | 0.863636 | 0.000612 | 0.036702 |
| USP6NL-liver | ELMOD2-fat | 0.681818 | 0.020843 | 0.117241 |
| LAP3-liver | ERCC8-fat | 0.718182 | 0.0128 | 0.100745 |
| STK40-liver | ERCC8-fat | 0.890909 | 0.000233 | 0.036702 |
| USP6NL-liver | ERCC8-fat | 0.672727 | 0.023313 | 0.123422 |
| STK40-liver | FIGN-fat | 0.709091 | 0.014552 | 0.100745 |
| USP6NL-liver | FIGN-fat | 0.754545 | 0.007282 | 0.093626 |
| ADRA2B-liver | HLTF-fat | 0.818182 | 0.002083 | 0.051172 |
| LAP3-liver | HLTF-fat | 0.872727 | 0.000455 | 0.036702 |
| STK40-liver | HLTF-fat | 0.827273 | 0.001677 | 0.051172 |
| USP6NL-liver | HLTF-fat | 0.790909 | 0.003746 | 0.067429 |
| LAP3-liver | KLHDC10-fat | 0.709091 | 0.014552 | 0.100745 |
| STK40-liver | KLHDC10-fat | 0.7 | 0.016471 | 0.105885 |
| ADRA2B-liver | LXN-fat | 0.609091 | 0.046696 | 0.170011 |
| LAP3-liver | LXN-fat | 0.818182 | 0.002083 | 0.051172 |
| STK40-liver | LXN-fat | 0.672727 | 0.023313 | 0.123422 |
| USP6NL-liver | LXN-fat | 0.627273 | 0.038845 | 0.152003 |
| STK40-liver | MRPS31-fat | 0.809091 | 0.002559 | 0.051172 |
| LAP3-liver | NFIC-fat | 0.745455 | 0.008455 | 0.095116 |
| LAP3-liver | NID2-fat | 0.7 | 0.016471 | 0.105885 |
| STK40-liver | NID2-fat | 0.727273 | 0.011205 | 0.100745 |
| LAP3-liver | NIF3L1-fat | 0.690909 | 0.018565 | 0.115231 |
| STK40-liver | NMB-fat | 0.763636 | 0.006233 | 0.086304 |
| USP6NL-liver | NMB-fat | 0.745455 | 0.008455 | 0.095116 |
| LAP3-liver | NNT-fat | 0.645455 | 0.031963 | 0.140324 |
| LAP3-liver | NR2F6-fat | 0.718182 | 0.0128 | 0.100745 |
| STK40-liver | PDCD10-fat | 0.654545 | 0.028865 | 0.136727 |
| ADRA2B-liver | PON2-fat | 0.709091 | 0.014552 | 0.100745 |
| LAP3-liver | PON2-fat | 0.809091 | 0.002559 | 0.051172 |
| STK40-liver | PON2-fat | 0.627273 | 0.038845 | 0.152003 |
| STK40-liver | PSME3IP1-fat | 0.681818 | 0.020843 | 0.117241 |
| STK40-liver | SLC25A20-fat | 0.618182 | 0.042646 | 0.159921 |
| USP6NL-liver | SLC4A4-fat | 0.663636 | 0.025984 | 0.126409 |
| ADRA2B-liver | SNRNP25-fat | 0.627273 | 0.038845 | 0.152003 |
| LAP3-liver | SNRNP25-fat | 0.709091 | 0.014552 | 0.100745 |
| STK40-liver | SNRNP25-fat | 0.709091 | 0.014552 | 0.100745 |
| USP6NL-liver | SNRNP25-fat | 0.772727 | 0.005299 | 0.079492 |
| STK40-liver | TIMM21-fat | 0.827273 | 0.001677 | 0.051172 |
| STK40-liver | TIMMDC1-fat | 0.663636 | 0.025984 | 0.126409 |

**Supplement Table 10 represents the top 5% in Liver-39 gene centrality and in Fat-5, Liver-39 and Fat-43 and Liver-39 and Fat-19 with calculated Spearman correlations between the two groups, focusing only on strong correlations of over I0.6I with a p<0.05.**

**Supplement Table 11: GO enrichment pathways for key gene clusters of interest.**

**Liver-1 gene cluster**

Angiogenesis

Cell adhesion molecule binding

Cell substrate junction

Focal adhesion

Actin cytoskeleton

Positive regulation of vacuole organisation

Cell substrate adhesion

Cadherin binding

Regulation of vasculature development

**Liver-2 gene cluster**

Peptide biosynthetic process

Translation

Ribonucleoprotein complex

Organelle inner membrane

Endomembrane system organisation

Ribonucleoprotein complex biogenesis

ncRNA metabolic process

Regulation of translation

Ribosome

Ribonucleoprotein complex binding

**Muscle-5 gene cluster**

Cellular respiration

Aerobic respiration

Inner mitochondrial membrane protein complex

Oxidative phosphorylation

Respirasome

Organelle inner membrane

Mitochondrial inner membrane

Mitochondrial matrix

Mitochondrial protein-containing complex

Energy derivation by oxidation of organic compounds

**Muscle-35 gene cluster**

Glucose-sensing genes

**Fat-1 gene cluster**

2 iron 2 sulphur-cluster binding

Organelle inner membrane

Mitochondrial inner membrane

Ribonucleoprotein complex

Mitochondrion organisation

Mitochondrial protein-containing complex

**Supplement Table 11** shows that, using GO enrichment pathways for the key gene clusters of interest, Liver-1 genes were associated with extracellular matrix and fibrosis, whereas Liver-2 genes were associated with ribosome function and proliferation. Muscle-5 genes related to mitochondrial function and respiration, Muscle-35 contained glucose-sensing genes, and Fat-1 genes were related to mitochondrial function
